# Contemporary Developments in Ferrocene Chemistry: Physical, Chemical, Biological and Industrial Aspects

**DOI:** 10.3390/molecules28155765

**Published:** 2023-07-30

**Authors:** Umair Rauf, Ghulam Shabir, Saba Bukhari, Fernando Albericio, Aamer Saeed

**Affiliations:** 1 Department of Chemistry, Quaid-I-Azam University, Islamabad 45320, Pakistan; urauf@chem.qau.edu.pk (U.R.); shabirg@yahoo.com (G.S.); sababukhari021@gmail.com (S.B.); 2 Peptide Science Laboratory, School of Chemistry and Physics, University of KwaZulu-Natal, Westville, Durban 4000, South Africa; 3 CIBER-BBN, Networking Centre on Bioengineering, Biomaterials and Nanomedicine, Department of Organic Chemistry, University of Barcelona, 08028 Barcelona, Spain

**Keywords:** ferrocene, synthesis, applications, asymmetric catalysis, chemosensors, bioreceptors

## Abstract

Ferrocenyl-based compounds have many applications in diverse scientific disciplines, including in polymer chemistry as redox dynamic polymers and dendrimers, in materials science as bioreceptors, and in pharmacology, biochemistry, electrochemistry, and nonlinear optics. Considering the horizon of ferrocene chemistry, we attempted to condense the neoteric advancements in the synthesis and applications of ferrocene derivatives reported in the literature from 2016 to date. This paper presents data on the progression of the synthesis of diverse classes of organic compounds having ferrocene scaffolds and recent developments in applications of ferrocene-based organometallic compounds, with a special focus on their biological, medicinal, bio-sensing, chemosensing, asymmetric catalysis, material, and industrial applications.

## 1. Introduction

Ferrocene is an organometallic compound consisting of two cyclopentadienyl rings bound to a central iron atom. On account of its unique electronic and structural properties, ferrocene has found a wide range of applications in various fields, such as fuel additives to improve their performance and reduce emissions; electrochemical sensors to detect the presence of various molecules and ions in solutions, including glucose, dopamine, and heavy metals; catalysts in various organic reactions, including oxidation, hydrogenation, and cross-coupling reactions; and medicinal chemistry in the development of anticancer, antiviral, and antimicrobial drugs. Ferrocene derivatives have been used to synthesize a wide range of materials, including polymers, liquid crystals, and nanoparticles, with diverse electronic and magnetic properties. Ferrocene is used as a potential electrode material in redox flow batteries due to its reversible redox behavior and high stability, and as a lubricant to improve the performance and longevity of engines and industrial machinery. The discovery of ferrocene and its structural characterization initiated an explosive rebirth of organometallic chemistry [1]. Nowadays, ferrocene derivatives have received much consideration owing to their outstanding properties like thermal and photochemical inertness and their exceptional and stable electrochemical properties due to an electron donor–acceptor conjugated structure and an efficient redox couple of Fe^2+^/Fe^3+^ [2,3,4]. The development of ferrocene unveiled new horizons of chemistry that are deeply rooted in the interpretation of its structure, way of bonding, and chemical inertness, and paved the way for the escalating field of organometallic chemistry itself [5]. Ferrocene and its derivatives are laurel molecules, and the synthesis of this sandwich system—specifically its flavors, utilization in homogeneous catalysis as a novel resource, including its usage in polymer chemistry, and also as a fuel additive—has triggered the hasty advancement of this family of compounds over the past sixty years [6,7,8]. Polymers with pendant ferrocene units are a marvelous family of organometallic polymers elevating substantial attention, chiefly owing to their idiosyncratic properties and the high chemical inertness of ferrocene [9]. These polymeric materials, either as ferrocenyl or ferrocendiyl groups, deliver various effects including conducting, redox, optoelectronic, catalytic, and biological effects with potential for applications such as radiation absorbers, combustion regulators, highly effective non-toxic medicinal substances, components of various redox systems, etc. [10]. Ferrocene-grafted polymers can easily migrate from the propellant system through diffusion as well as by surface migration. This migration leads to further abolishing the propellant homogeneity and allows higher combustion rates only on the surface of the propellant rather than the whole propellant [11]. Recent trends led to the development of established drugs by metallocene-appended organometallic moieties through drug reprofiling strategy as a proxy of the chemotherapy of tachyphylaxis in cancer and many tropical diseases [12]. The substitution of the phenyl or hetero-aryl group with a ferrocene scaffold in organic molecules afforded a momentous alteration not only in the physicochemical properties of drugs, but also ameliorated the potential of biologically active ligands. Moreover, the success of ferrocene owes to its intrinsic solidity in air, heat, and light, as well as low toxicity, low cost, and reversible redox properties. In the presence of oxidizing agents, the oxidation of ferrocene to ferrocenium cation occurs, which is characterized by the naked eye, i.e., red coloration. The well-known application of this oxidized ferrocene comprises sensing, corrosion inhibition, and drug delivery in cancer therapy [13,14,15]. Indeed, based on an extensive database of knowledge regarding ferrocene, numerous articles have been published in several fields concerning applications like water treatment, lithography, information storage, molecular logics, pharmacology, agriculture, biosensors, chemosensors, analytical applications, etc., during 2010–2020. Also, ferrocene derivatives are leading targets primarily for power conversion, and their thermo- and electrochemical applications have been extensively studied due to astonishing features like high thermal stability, higher photoconductivity, and the better chemical stability of ferrocene derivatives [16].

In the past ten years, due to the iron chemistry of ferrocene derivatives, there has been a spate of review articles on the synthesis and applications of ferrocenyl conjugates, which include the research areas of materials science, asymmetric catalysis [17], electrochemistry, and bio-organometallics [18,19]. Saeed et al. reviewed the synthesis, biological activities, and some important applications of ferrocene derivatives [20]. X. Zhai et al. published a review on recent research progress in the synthesis, properties, and applications of ferrocene-based derivatives and polymers with azobenzene [21]. In 2017, a review article with the title “Ferrocene-based redox-responsive polymer gels: Synthesis, structures and applications” was published by Jialiang and co-workers [22]. Similarly, A. Khan and colleagues presented another review on the synthesis and applications of ferrocene-based electro- and photo-responsive materials in 2018 [23]. During 2015–2019, more focused reviews seemed to cover medicinal outlooks including anti-malarial and anticancer activities of ferrocene derivatives [24], antimicrobial activities of ferrocene conjugates [25], tetrazole-based ferrocenyl compounds as anti-tuberculosis agents [26], and the anti-infective potential of ferrocene derivatives [27].

The aim of our work is to provide a comprehensive review of recent trends in synthetic strategies of ferrocene derivatives and their versatile pharmacological, agricultural, biosensing, chemosensing, biological, electrochemical, industrial, and materials science applications, covering the period from 2016 to the start of 2023.

## 2. Synthesis of Different Ferrocene-Based Compounds

### 2.1. Synthesis of Ferrocenyl Ethers

**Scheme 1:**
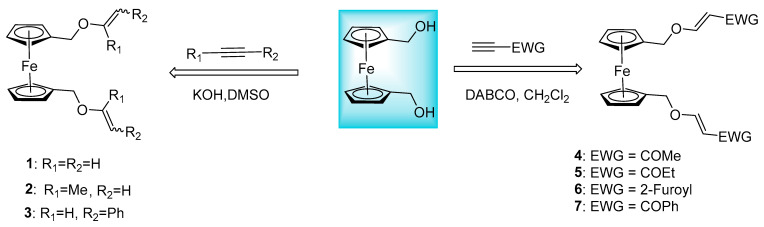
Synthesis of ferrocene-based diethenyl ethers.

Ferrocene-containing aliphatic and aromatic ethers are of special interest due to their beauty and electrochemical properties, but their synthesis has always been difficult. To respond to this challenge, Oparina and co-workers introduced a novel nucleophilic addition reaction of ferrocene 1,1-bis(hydroxymethyl) to different alkyne derivatives (Scheme 1). This base-catalyzed addition could be performed on acetylene, propyne, phenylethyne, alkyl propionates, and acylacetylenes to obtain various divinyl ferrocenyl ethers with a remarkable yield value in the range of 73–98% [28].

**Scheme 2:**
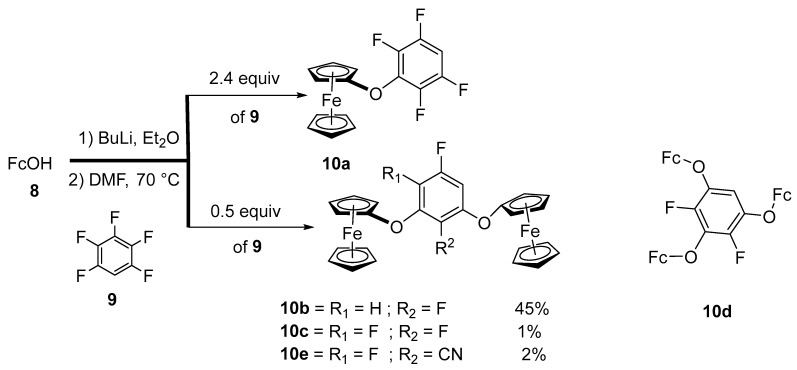
Synthesis of ferrocenyl aryl ethers.

In a relevant work, multi-ferrocenyl aryl ethers of general type C_6_H_0-3_F_1-5_(OFc)_1-5_ were synthesized through base-catalyzed nucleophilic aromatic substitution reaction on aryl fluorides. Preparation of mono-, di-, and trisubstituted fluorobenzene was simply carried out through the replacement of one, two, and three fluorine atoms byFcO, respectively (Scheme 2). Further tetra and penta (FcO)-substituted arenes were also prepared by the same procedure and identified by spectroscopic analysis, and XRD confirmed their substitution patterns [29].

**Scheme 3:**
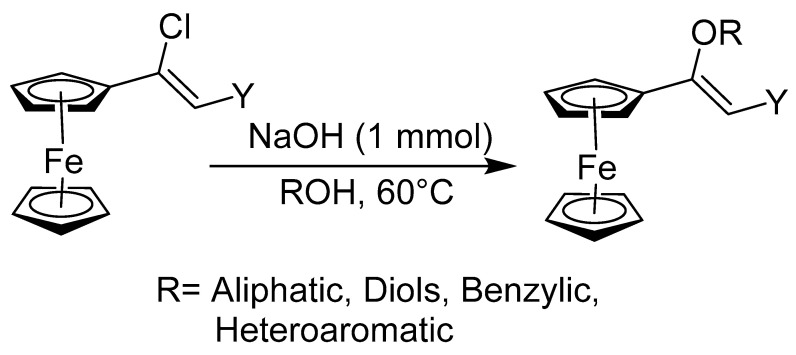
Synthesis of ferrocene-substituted vinyl ethers.

It has been reported that different primary alcohols and ferrocene chlorovinyl derivatives can form C-O bonds easily, leading to the production of ferrocenyl vinyl ethers (Scheme 3). The approach that is being offered has a high yield and provides the products quickly. For a variety of primary alcohols, functionalized ferrocenyl chlorovinyl derivatives (-CHO, -CN, and C(CN)_2_) were shown to be well tolerated. All the ferrocenated vinyl ethers are novel and have not been previously reported. When the optical metal-detecting capabilities of the 1,2- and 1,3-diol-derived vinyl ether derivatives were examined, selectivity for Cu^2+^ ions was found. Among all the other metal ions put to the test, the ferrocenated malenonitrile-containing vinyl ether showed high sensitivity and selectivity toward Cu^2+^ [30].

**Scheme 4:**
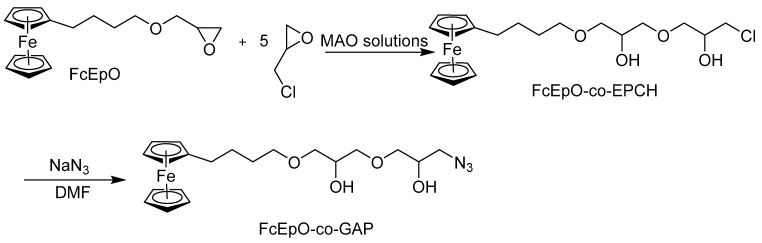
Synthesis of poly (FcEpo-co-EPCH) and poly (FcEpo-co-GAP).

By polymerizing 2-[(4-ferrocenylbutoxy) methyl] oxirane (FcEpo) using toluene solution of methylaluminoxane (MAO) as the catalyst, poly(ferrocenyl glycidyl ether) was synthesized. In order to create another ferrocenyl-based poly, 2-[(4-ferrocenylbutoxy) methyl] oxirane and epichlorohydrin were copolymerized (epichlorohydrin). GAP, a ferrocenyl-based poly (glycidyl azide), was created by treating sodium azide with this copolymer in a room-temperature DMF solvent (Scheme 4). By using FT-IR, ^1^H NMR, UV-VIS, TGA, DSC, and GPC analysis, the synthesized ferrocenyl-based polymers were evaluated for their characteristics [31].

### 2.2. Synthesis of Alkynylated Ferrocenes

**Scheme 5:**
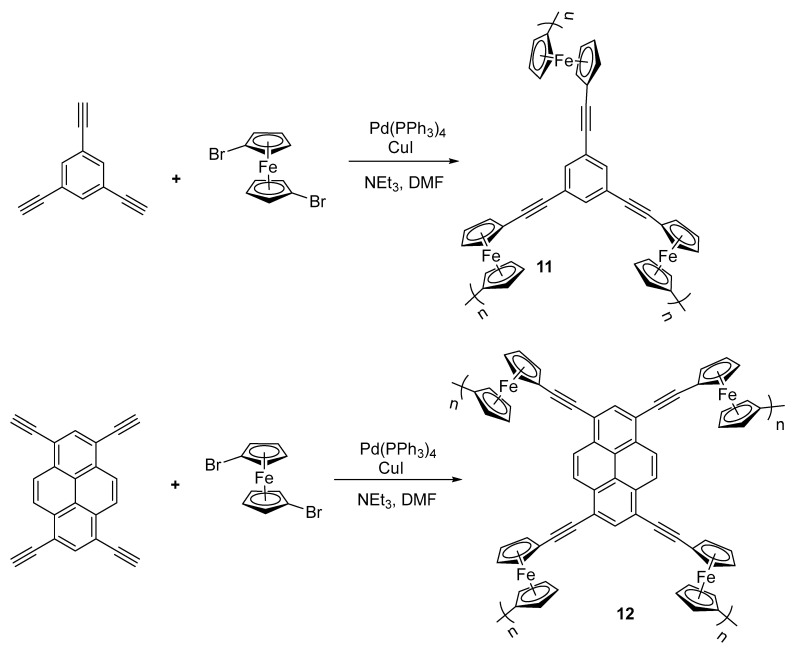
Synthesis of ferrocene-functionalized triethynyl and tetraethynyl benzene.

In a promising work, ferrocene was grafted to microporous polymers of 1,3,5-triethynyl benzene and 1,3,6,8-tetraethynyl analogs using Pd-catalyzed Sonogashira Hegihara cross-coupling chemistry (Scheme 5). The molecular structures of these compounds were confirmed by FTIR and ^13^C NMR spectroscopy, and the presence of iron was confirmed through X-ray photoelectron spectroscopy. These synthesized compounds exhibited higher thermal stability, porosity, and variable ferromagnetic properties [32].

### 2.3. Synthesis of Vinyl and Aryl-Substituted Ferrocene

**Scheme 6:**
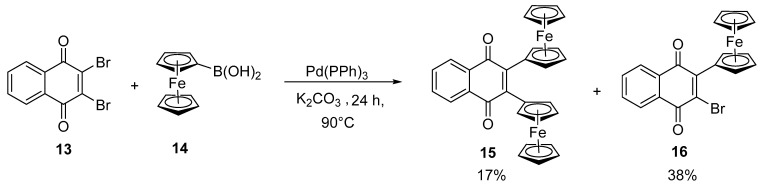
Suzuki–Miyaura coupling reaction.

Ertas and co-workers used the Pd-assisted Suzuki–Miyaura coupling method to synthesize ferrocenyl–naphthoquinone derivatives, and their structure was characterized by ^1^H NMR and ^13^C NMR spectroscopy (Scheme 6). This coupling reaction was also performed through microwave irradiation, but the yield was not as effective as compared with the reported protocol. Furthermore, the reported compound **16** was thought to be the first innovative ferrocene-based H_2_O_2_ chemosensor [33].

**Scheme 7:**
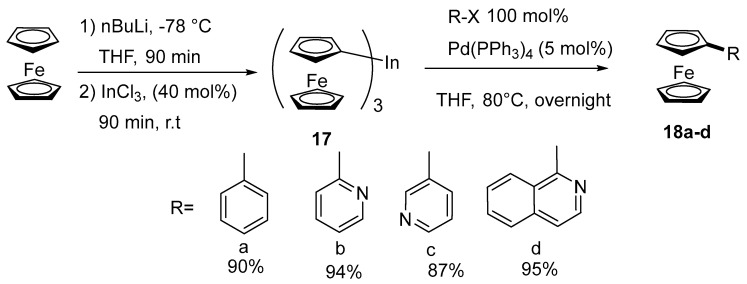
Synthesis of mono-substituted ferrocene derivatives through coupling reaction.

Mato described a procedure for the preparation of mono and planer asymmetric disubstituted ferrocenyl conjugates by using tri-ferrocenylindium intermediate followed by a Pd-assisted cross-coupling reaction [34]. Under mild conditions, a series of organic electrophiles were treated with Fc_3_In (**17**) for the synthesis of monosubstituted ferrocenyl compounds **18_a–d_**. Plane chiral disubstituted ferrocenyl conjugates were also prepared by the same coupling mechanism (Scheme 7).

**Scheme 8:**
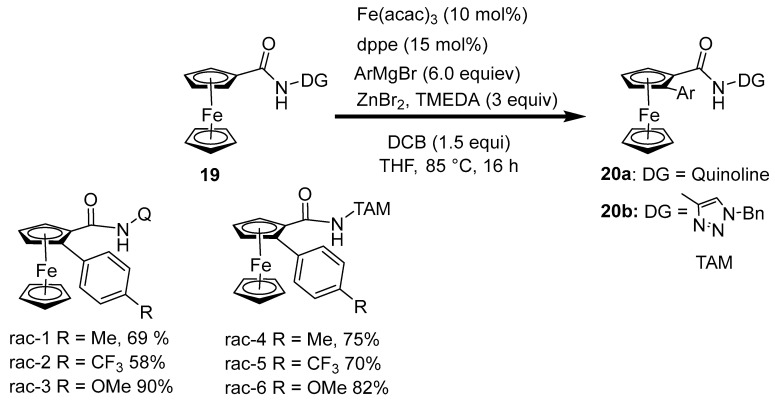
Synthesis of ortho-substituted ferrocenyl conjugates.

In an advanced work, Schmiel and co-authors reported a novel procedure for the ortho-substitution of ferrocenyl units by C–H activation using sp^2^–sp^2^ coupling reactions [35]. The excellent yield of methylation and ethylation was observed, while stereoselective phenylation of **20a** and **20b** with asymmetric ligand resulted in a remarkable yield of a product with 46% of ee (Scheme 8).

**Scheme 9:**
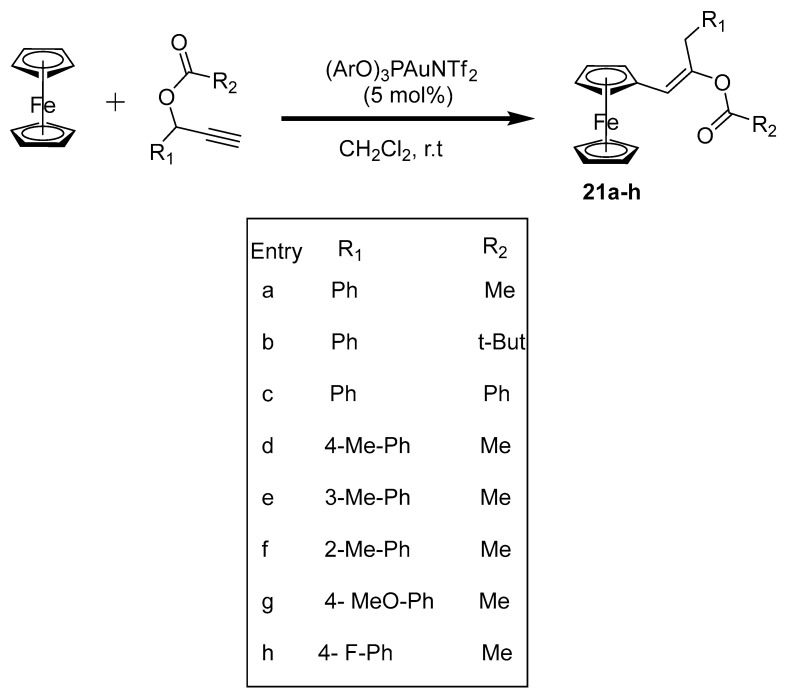
Synthesis of ferrocene-based prop-2-ynoic acid derivatives.

Lopez’s group focused their research on the development of a novel gold-catalyzed C–H activation reaction of ferrocene-appended prop-2-ynoic acid [36]. The reaction proceeded through the substitution of ferrocene by capturing vinyl gold carbenoid, generated from propargylic ester. Isotopic labeling experiments described the mechanism as an electrophilic substitution reaction in which 1–2 rearrangements produced metal vinyl carbene that acts as an electrophile that was attacked by ferrocene derivatives via C–H activation (Scheme 9).

**Scheme 10:**
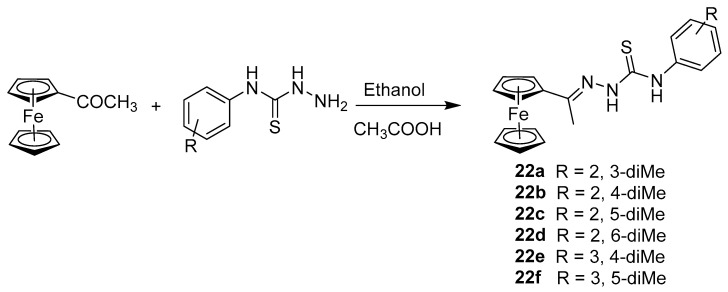
Synthesis of ferrocene-based thiosemicarbazones.

Ferrocene and its derivatives represent a crucial class of organometallic compounds with significant biological activity. By condensing acetyl ferrocene with variously substituted thiosemicarbazides, six new ferrocene-based thiosemicarbazones have been created (Scheme 10). Furthermore, the theoretical features for putative antiviral properties of produced compounds were investigated using cutting-edge computational docking methodology. The Mpro protein of SARS-CoV-2, an essential protein for viral replication, was docked with all six chemicals [37].

**Scheme 11:**
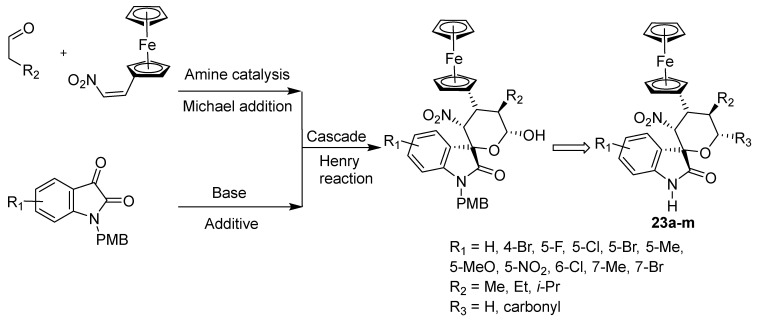
Asymmetric synthesis of chiral spirocyclic oxindole–ferrocene hybrids.

Organocatalysis was used to create several spirooxindole–ferrocene hybrids with four or five contiguous chiral centers. Additionally, mechanistic studies revealed that compound **23d** enhanced oxidative damage, caused apoptosis, and reduced MDM2-mediated p53 degradation (Scheme 11). According to molecular docking research, **23d** attaches to MDM2 by imitating the Trp23 and Leu26 residues of p53. This research may serve as a foundation for the creation of innovative, multipurpose MDM2 inhibitors. The research of other derivatives from this library and the application of organocatalysis in the creation of novel molecules may result in the discovery of new potential lead compounds for cancer-targeted therapy [37].

**Scheme 12:**
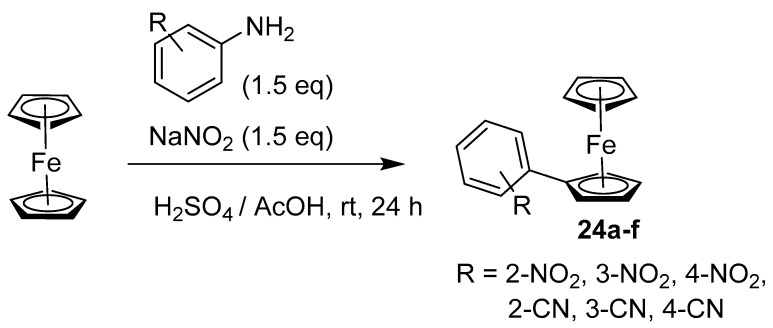
Synthesis of phenyl ferrocene derivatives.

Ferrocene is a typical organometallic compound, which has a sandwich structure with strong hydrophobicity and stability. In this study, the nitro- and cyanophenylferrocene series’ physicochemical characteristics were identified, and their biological activity as androgen receptor (AR) antagonists was assessed (Scheme 12). Inferring that the hydrophobicity of ferrocene is ideal for its use as a hydrophobic core structure of nuclear receptor ligands, ferrocene derivatives showed hydrophobicity parameter values in the range of 2.54 to 3.23, depending on the substituents. The synthetic ferrocene derivatives demonstrated AR-antagonistic action, and 3-nitrophenylferrocene **24a** was the most effective of these, with an IC_50_ value of 0.28 M. As AR antagonists, the discovered compounds might be suitable for further structural development. These results confirm the usefulness of organometallic substances as structural options for drug discovery [38].

**Scheme 13:**
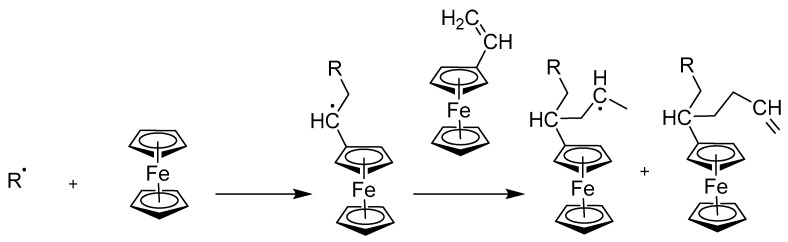
Synthesis of poly (vinyl-Fc) by FRP of vinyl-Fc.

Effective composite solid propellants have low-pressure exponents and a consistent burning rate (BR). The addition of burning rate catalysts (BRCs) to the propellant system is one of the finest options for achieving the optimal performance of propellants (Scheme 13). BRCs are incredibly significant in composite propellants because of their unique ability to increase the operating effectiveness of solid propellants. Numerous beneficial varieties of BRCs were synthesized for this purpose. Ferrocene-based compounds are a well-known type of BRC due to their superior ignition. However, the practical application of this new family of beneficial compounds as an ideal BRC is constrained by their significant migration and diffusion propensity during storage operations of composite solid propellants [39].

### 2.4. Synthesis of Alkyl and N-Heteroatom-Substituted Ferrocene Derivatives

**Scheme 14:**
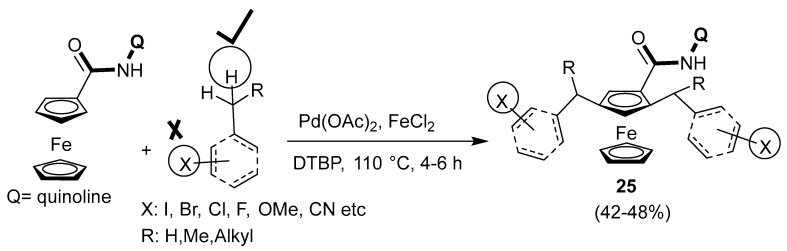
Alkylation of ferrocenyl compounds by means of Pd-Fe hybrid catalyst.

Satter and Kumar reported a new pathway for C-C bond formation between ferrocene and other alkyl or aryl substituents catalyzed by hybrid transition metal catalysts [40]. The reported work indicated an innovative sp^2^–sp^3^ coupling reaction for the first time where the C–H bond was coupled for new C-C bond formation, chiefly directed by ligands (Scheme 14). The reaction could be a useful approach for the synthesis of ferrocene-functionalized biologically active drugs.

**Scheme 15:**
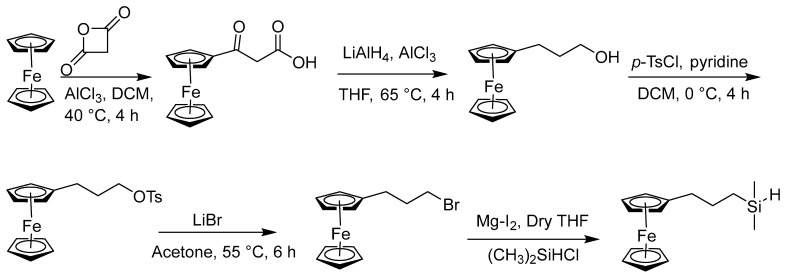
Schematic representation for the synthesis of *n*-(dimethylsilane)alkyl ferrocene.

The synthesis of thiol, silyl, and halogen derivatives of alkyl-ferrocene was made simple and affordable. In this procedure, ferrocene is functionalized with terminal carboxylic acid using cyclic anhydrides as a precursor to be purified using an acid–base workup. With good to exceptional yields, the synthetic technique uses moderate reaction conditions (Scheme 15). To create polymeric binders with a faster burn rate for usage in composite solid propellant for aerospace applications, the ferrocene-alkyl silane and ferrocene-alkyl-thiol are covalently grafted to the pendent vinylic groups of HTPB [41].

**Scheme 16:**
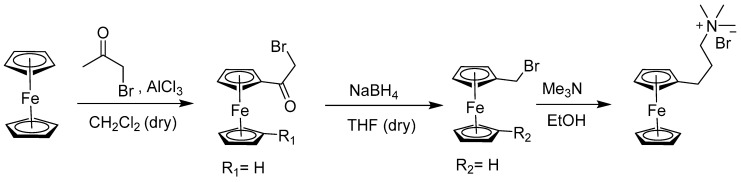
Synthetic route for the ferrocene derivatives.

By utilizing the redox reactions of water-soluble organic molecules, an aqueous organic flow battery (AOFB) holds immense potential as an energy storage solution for fluctuating renewable electricity (Scheme 16). The absence of suitable organic molecules for catholytes is impeding current progress; however, it is yet unknown how catholyte structure affects battery longevity. In water, six ferrocene derivatives with specifically tuned chemical structures go through reversible redox processes, and the redox potentials of these energized forms are inversely related to their LUMO energies. In symmetric and complete flow cells, the stability of the ferrocene derivatives was assessed. Calculations using density function theory (DFT) and actual findings show that the localized LUMO density on Fe causes a rapid capacity fading, with BQHFc having the lowest LUMO density [42].

### 2.5. Synthesis of Ferrocenyl Derivative Directly Bonded with Heteroatom

**Scheme 17:**
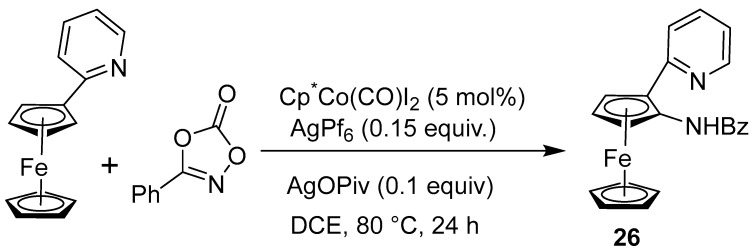
Cyclopentyl cobalt (III)-assisted synthesis of N-substituted ferrocene derivatives.

Recent research reported the synthesis of cyclopentyl cobalt (III)-assisted carbon–heteroatom coupling reaction on a gram scale, using pyridylferrocene and dioxazolones under soft conditions with an impressive yield of 96% (Scheme 17). These ferrocene-based compounds could be used as effective catalysts due to the presence of N, N-bidentate ligands. Further, the replacement of the directing group could increase the efficiency of the reaction column, and the absolute configuration of selected enantiomers was determined by X-ray crystallographic analysis [43].

**Scheme 18:**
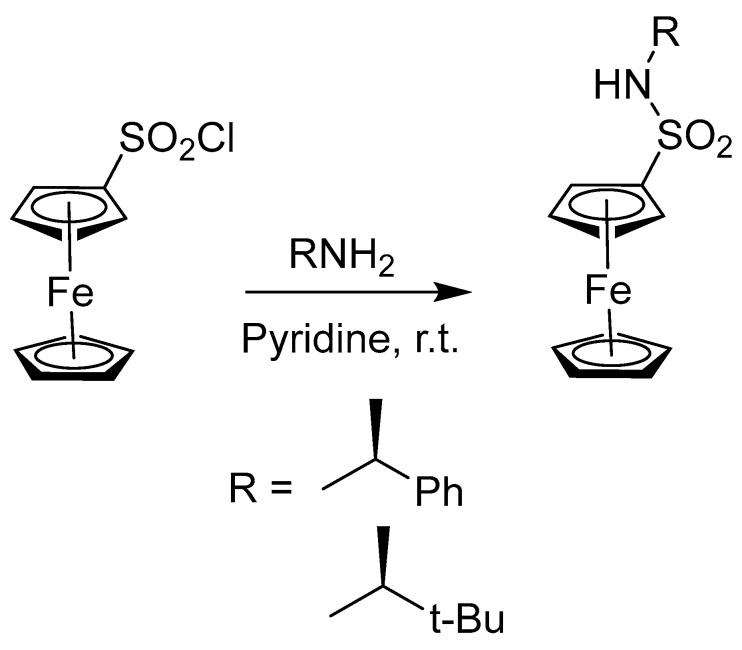
Synthesis of chiral ferrocene sulfonamides.

The *ortho*-directed lithiation of chiral ferrocene sulfonamides, followed by a reaction with electrophilic reactants, was used to synthesize a number of 1,2-disubstituted planar chiral ferrocenes (Scheme 18). The various diastereoisomers could be separated in pure form, and the planar 1,2-functionalized ferrocene sulfonamides were produced in good yields. The planar chirality-relevant relative configurations of the chemical groups in positions 1 and 2 were recovered from NMR measurements, and the absolute configuration was established by X-ray crystallography. Modern quantum chemical algorithms were used to record, assign, and interpret experimental ECD and VCD spectra, allowing for an impartial examination of the several structural elements that influence the overall spectroscopic properties [44].

**Scheme 19:**
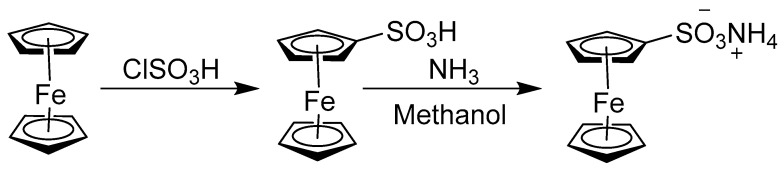
Synthetic route of Fc-SO_3_NH_4_.

The synthesis and application of redox-active organic species in aqueous redox flow batteries offer numerous opportunities for extensive and sustainable energy storage, which is necessary for sustainable development. Here, a stable and simple-to-synthesize sulfonated ferrocene derivative known as ferrocene ammonium sulfonate is disclosed. In zinc hybrid flow batteries, it served as a catholyte. With the electrolyte adjusted, the battery demonstrated good efficiency and capacity retention. After 100 continuous charge/discharge cycles with a current density of 20 mA/cm^2^ and a supporting electrolyte of 0.5 M NaCl + 0.5 M NH_4_Cl, the capacity retention achieved 92.55% (Scheme 19). This battery design proposes a concept for developing low-cost materials and improving capacity retention [45].

### 2.6. Synthesis of Ferrocene-Conjugated Metal Complexes

**Scheme 20:**
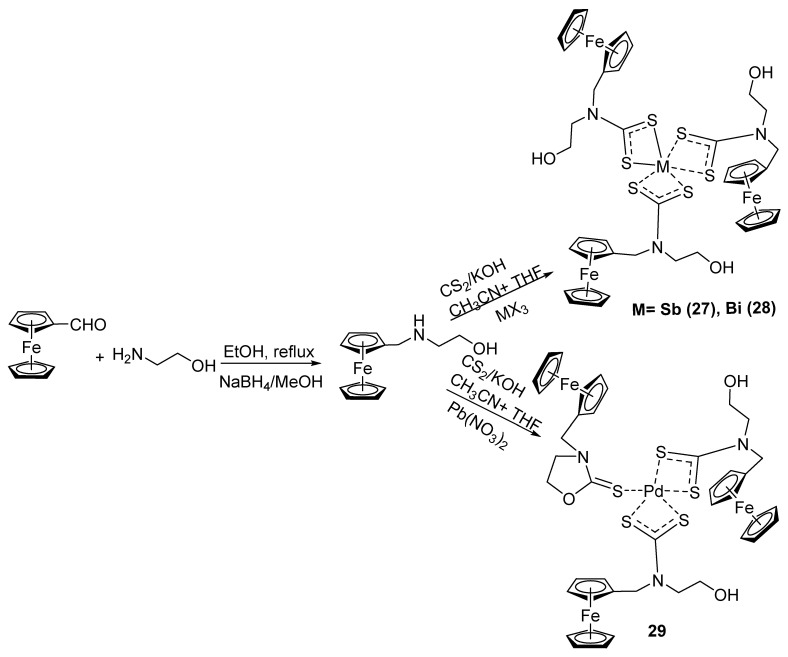
Synthesis of ferrocene-grafted metal complexes.

Three ferrocenyl-functionalized metal complexes (**27**–**29**) were synthesized using a newly reported synthetic procedure in which the ferrocene nucleus was converted to carbamate precursors that further developed into thiazolidine-containing metal complexes (Scheme 20). The geometry of these organometallic complexes was confirmed by spectroscopic studies and the XRD approach. All three complexes found their applications in the biological system, especially in a bacterial metabolic pathway study [46].

**Scheme 21:**
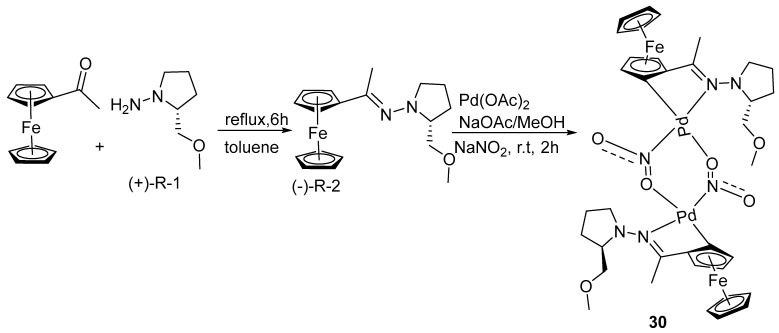
Synthesis of ferrocene-based cyclopalladated complex.

Gao and colleagues reported a rapid and efficient method for the synthesis of an advanced ferrocene-functionalized cyclopalladated complex by the reaction of acetyl ferrocene and chiral amines that produced orange-colored chiral ferrocenyl hydrazones that further reacted with Pd(OAc)_2_ and generated red-colored cyclopalladated product **30** (Scheme 21). Biological screening revealed that the potency of the cyclic organometallic complex was 93-fold greater than the usual anticancer drug cisplatin [47].

**Scheme 22:**
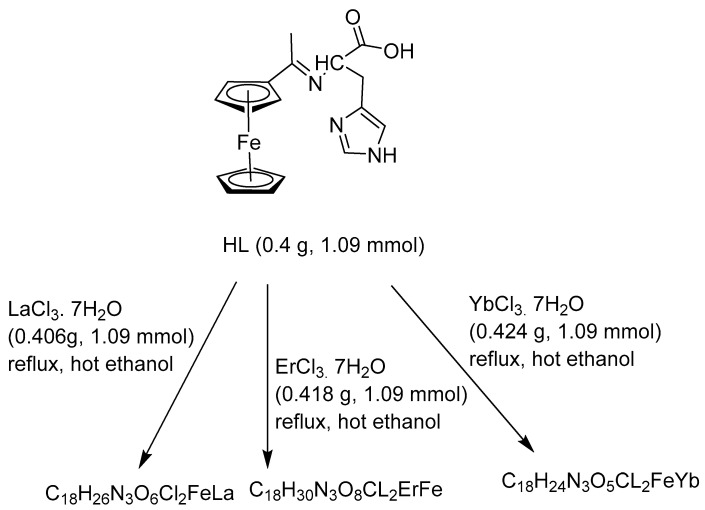
Synthesis of lanthanides metal complexes.

As the primary components of proteins, amino acids are essential to all major biological processes. Schiff bases are biologically active molecules with a variety of pharmacological functions. Their La (III), Er (III), and Yb (III) metal complexes were accomplished as shown in Scheme 22. Different spectroscopic techniques, such as elemental analysis, FT-IR, mass, UV–Vis spectra, thermal analysis (TG), and scanning electron microscopy (SEM), were used to describe these metal complexes. The complexes were discovered to have octahedral geometry using the elemental analysis data, with the formula [M(L)(H_2_O)_2_Cl]Cl.nH_2_O (M = La(III), n = 2; Er (III), n = 4; and Yb (III), n = 1). According to what has been determined, carboxylic oxygen, imidazole nitrogen, and azomethine nitrogen play a role in the ligand’s mono-negative tridentate form of binding to the metal ions [48].

**Scheme 23:**
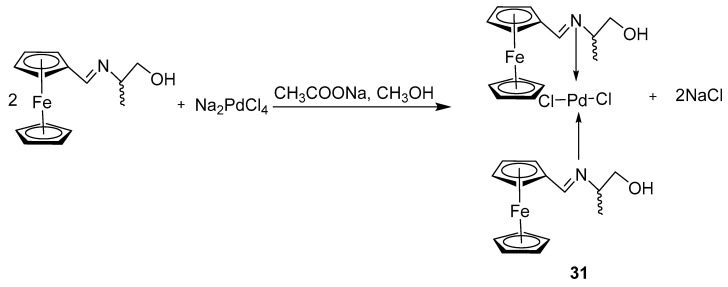
Synthesis of bimetallic complex with cross configuration.

A two-step process was used to produce an organic bimetallic complex with a cross configuration. The combination of ferrocene carboxaldehyde and DL-alaninol produced the ferrocene–palladium (II) complex, which in turn formed the ferrocene–Schiff base (Scheme 23). The crystal structure revealed that two motifs exist as a racemate and have a trans structure opposite to cisplatin. Compound **31** was found to be less hazardous to normal cells and to effectively decrease the viability of many tumor cell lines, with efficacy up to 20-fold better than cisplatin. The induction of caspase-3-dependent apoptosis in vitro served as the mechanism of antiproliferation actions. Molecular docking was used to predict that the biological target of the synthesized compound was XIAP, which showed it was bound to the BIR3 protein [49].

### 2.7. Synthesis of Ferrocene-Based Cyclic Urea

**Scheme 24:**
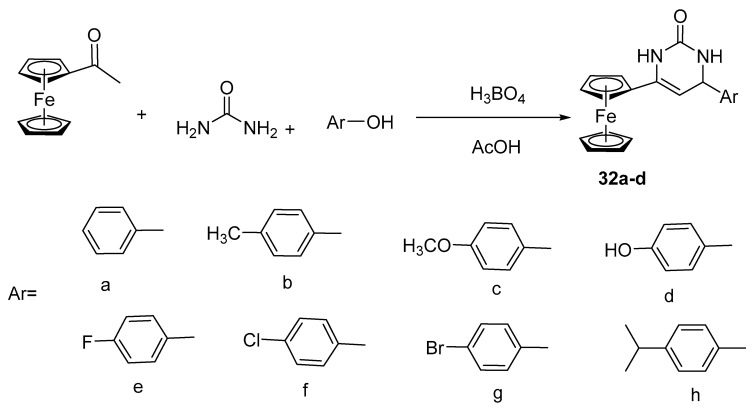
Synthesis of 3,4-dihydropyrimidines via Biginelli reaction.

In a recent study, a highly efficient, cost-effective, and environmentally benign protocol named “Biginelli Reaction” was utilized for the preparation of various para-substituted phenyl-6-ferrocenyl 3,4-dihydropyrimidine derivatives (**32_a–d_**) [50]. In a one-pot reaction, various substituted benzaldehyde units, acylated ferrocene, and urea components were mixed, and different products with different ranges of yield were isolated and characterized by spectroscopic analysis (Scheme 24). Biological assays proved these compounds as promising candidates for drug testing.

**Scheme 25:**
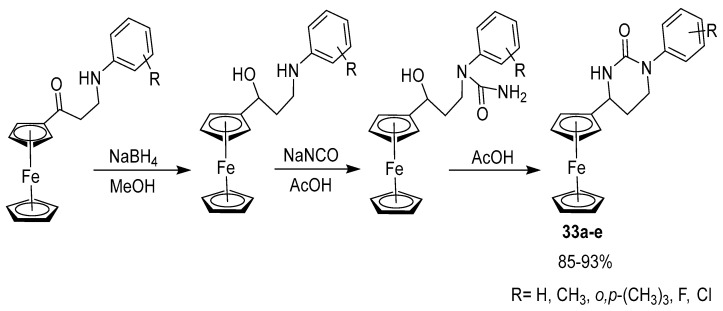
A synthetic strategy to 1-aryl-4-ferrocenyltetrahydropyrimidin-2(1*H*)-ones.

A process for synthesizing tetrahydropyrimidin-2(1*H*)-ones that contain ferrocene by starting with the appropriate 3-arylamino-1-ferrocenylpropan-1-ols and sodium cyanate (NaOCN) in the presence of glacial acetic acid was exercised. An intramolecular cyclization of 1,3-hydroxyurea produced in situ afforded eleven 1,3-amino alcohols containing ferrocene moiety, and the desired 1-aryl-4-ferrocenyltetrahydropyrimidin-2(1*H*)-ones (**33a–e**) were produced in good to high yields (up to 93%) (Scheme 25). Every product has been purified to a high level (>95%). A thorough structural characterization of the novel compounds, carried out using IR and NMR spectroscopy, is also described [51].

**Scheme 26:**
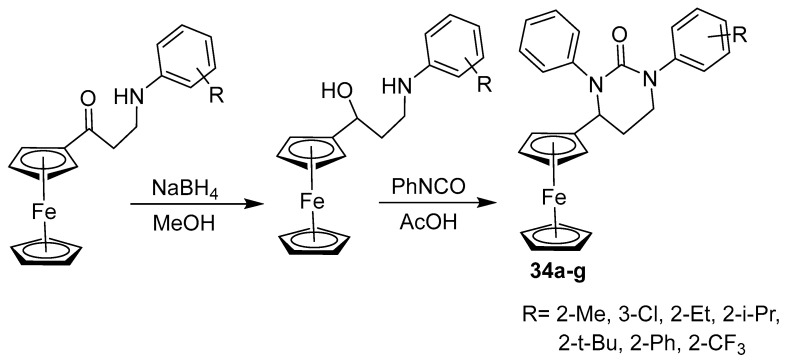
Substrate scope of synthesis of 1-aryl-4-ferrocenyl-3-phenyltetrahydropyrimidin-2(1*H*)-ones.

Atropoisomerism is a term used to describe a stereochemical phenomenon caused by impeded rotation around bonds in nonplanar molecules. An easy-to-perform synthesis and thorough characterization of novel atropoisomeric ferrocene-containing six-membered cyclic ureas (**34a**–**g**) is reported. This work was motivated by the intriguing biological/medical characteristics of six-membered cyclic ureas and “atropoisomers-things that rotate.” Two atropoisomers of the same compound’s crystal structure are also described. These two architectures’ molecular structural characteristics and intermolecular interactions have been compared and thoroughly examined. It is interesting to note that while having conformation and geometrical properties that are relatively comparable, these two isomers create significantly distinct crystal packing (Scheme 26) [52].

### 2.8. Synthesis of Ferrocene-Based Heterocyclic Derivatives

**Scheme 27:**
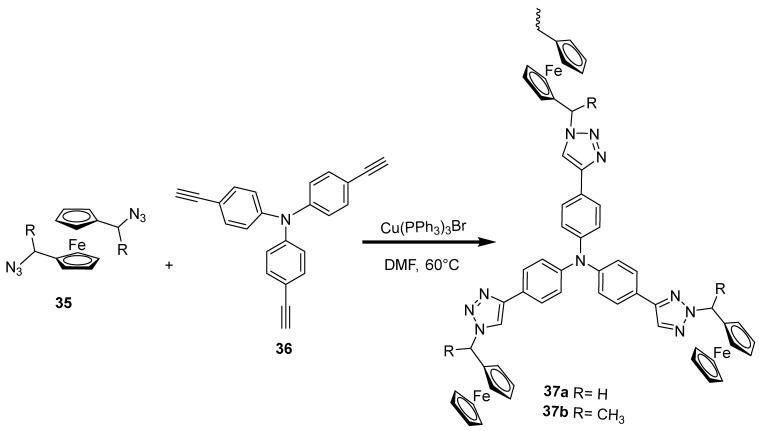
Synthesis of ferrocene-branched polytriazoles.

The group of Li and Chi have developed an elegant method for the construction of two ferrocene-branched poly-triazoles (**37a**–**b**) by treating ferrocene-based diazides (**35**) and tris (4-ethynylphenyl) amine (**36**) in the presence of a copper catalyst (Scheme 27). Owing to the ferrocene scaffold present inside poly-triazole-based polymers, two nanocomposites were prepared using carbonization or destructive distillation. Both polymeric materials also exhibited the saturation magnetization potential that proved the remarkable standard of these polymers for ultra-advanced technological applications [53].

**Scheme 28:**
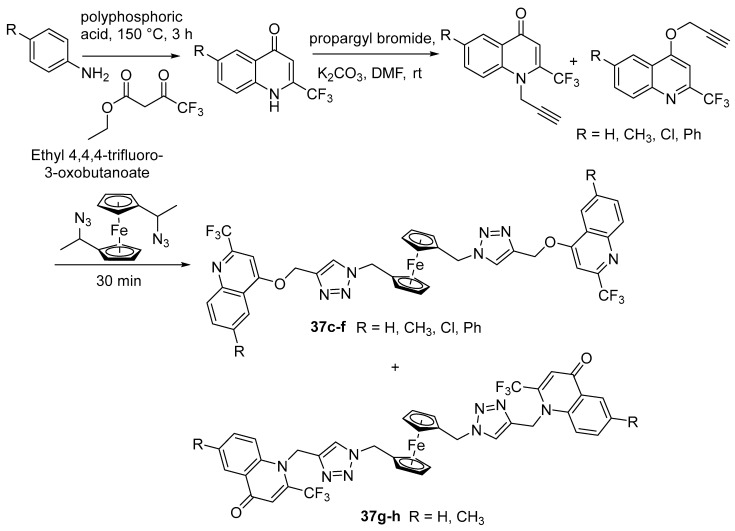
Mechanochemical synthesis and antiproliferative activity of novel ferrocene quinoline/quinolone hybrids containing bis-triazoles.

Maracic et al. conducted the synthesis of a series of novel bis-triazole containing 1,1′-disubstituted ferrocene conjugates (**37c**–**h**) attached with N-1 and O-4 alkylated quinolone and quinoline by the liquid-assisted grinding (LAG) mechanochemical method in copper-catalyzed azide–alkyne cycloaddition (CuAAC) in higher yields and with a shorter reaction time compared to a conventional method, thus proving the superiority of mechanochemistry versus conventional synthesis (Scheme 28). The antiproliferative effects of bis-quinoline and bis-quinolone ferrocene derivatives on five tumor cell lines and two non-tumor cell lines were investigated [54].

**Scheme 29:**
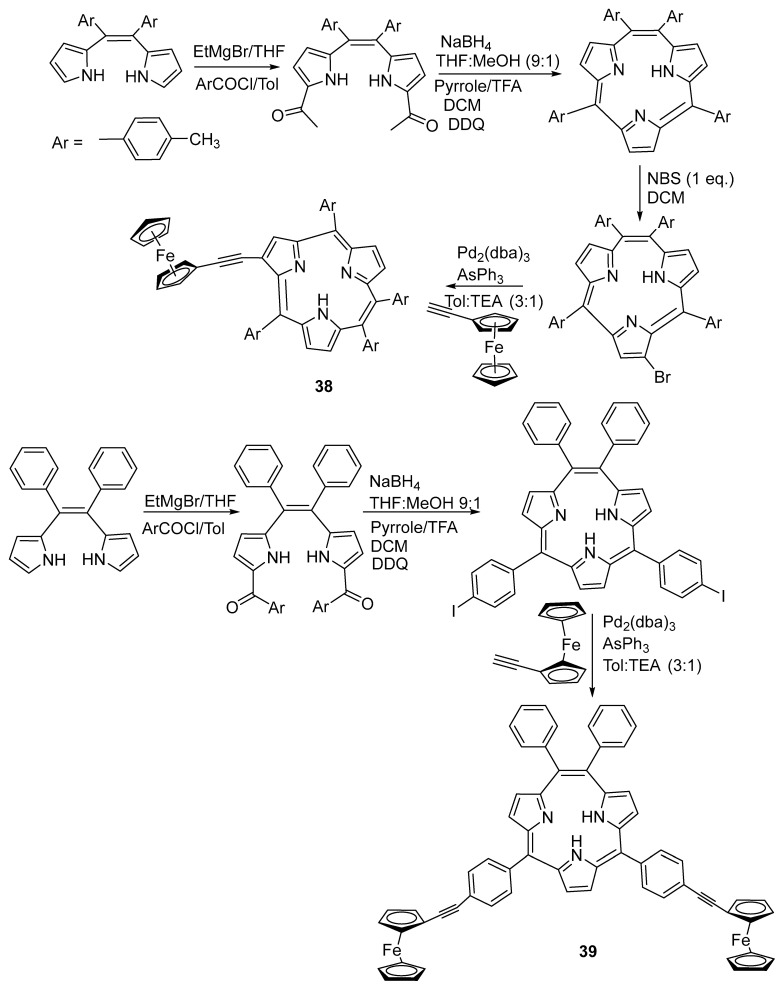
Synthesis of ferrocenyl-substituted triphyrin derivatives.

In addition, the synthesis of ferrocene-functionalized triphyrin analogs has been attempted using the Pd-promoted coupling reaction between substituted triphyrin and ethynyl ferrocene subunits under lenient conditions [55]. Further mechanistic study revealed that there was a strong affinity between triphyrins and ferrocenyl units in the diad (**38**) when compared with the triad (**39**). Both conjugates were thought to be promising candidates for evolutionary development in molecular device applications (Scheme 29).

**Scheme 30:**
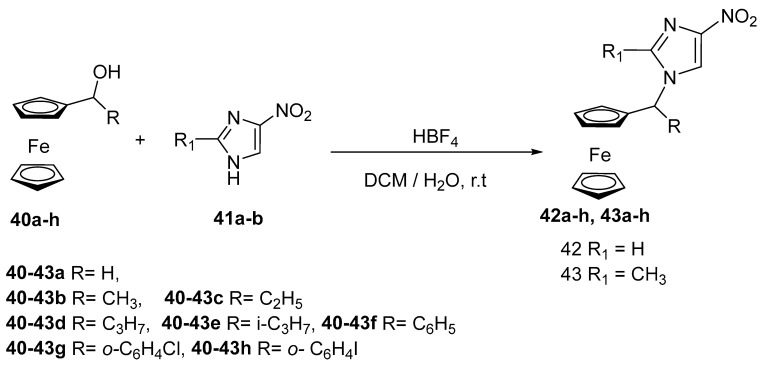
Regiospecific synthesis of ferrocene-functionalized nitroimidazoles.

Another interesting example regarding ferrocenyl heterocycles was the synthesis of ferrocene-grafted nitroimidazoles **42** in which a regiospecific reaction occurred between α-hydroxy ferrocene **40** and nitroimidazole precursor 41 in the presence of HBF_4_ (Scheme 30). The resulting racemic mixture was resolved by HPLC containing amylose or cellulose-based chiral stationary phase. Cytotoxicity assay of these derivatives revealed the anticancer activity of only compound **42b** against murine solid tumor system Ca755 cancer cells [56].

**Scheme 31:**
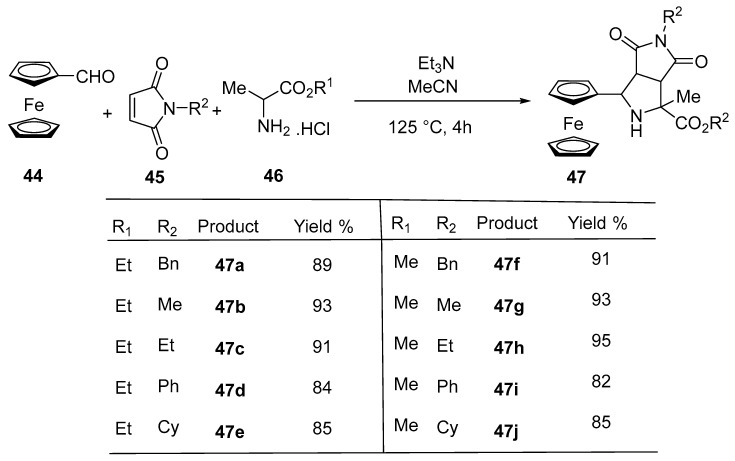
Synthesis of ferrocene-substituted pyrrolidine via dipolar cycloaddition reaction.

Zhang and co-workers introduced an innovative 1,3 dipolar cycloaddition protocol to synthesize a ferrocene-based substituted pyrrolidine system. In this novel approach, azomethine yields were generated through the in situ reaction of α-amino acid ester and ferrocene-functionalized carbaldehyde that further reacted with N-substituted maleimide to give a pyrrolidine product (Scheme 31). Moreover, the structures of these compounds were elucidated by ^1^H NMR and high-resolution mass spectral data [57].

**Scheme 32:**
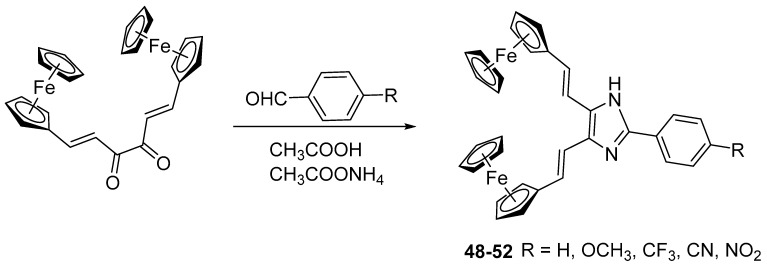
Synthesis of ferrocenyl imidazole derivatives.

The synthesis of ferrocenyl imidazole derivatives occurs through the Debus–Radziszewski condensation reaction in which 1,6-bis-ferrocene-hexa-1,5-diene-3,5-dione is condensed with *p*-substituted benzaldehyde, demonstrated by Selvam and co-workers (Scheme 32). These imidazole derivatives displayed second-order NLO properties confirmed by SHG analysis [58].

**Scheme 33:**
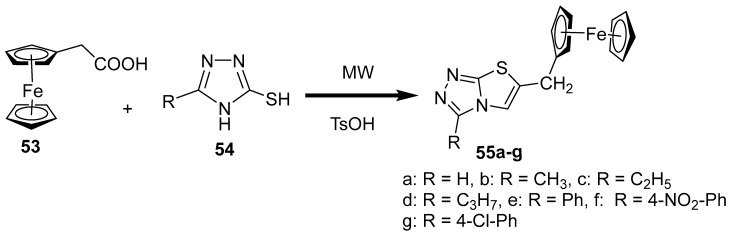
Microwave-assisted synthesis of ferrocenyltriazoles.

A microwave-assisted procedure for the synthesis of various ferrocene-functionalized triazole derivatives has been reported by Liu and co-authors [59]. The reaction between 2-amino-5-substituted-1,3,4-thiadiazole and α-bromoacetylferrocene as substrates in the presence of *p*-toluenesulfonic acid under microwave irradiation offered high yields of ferrocenylimidazolo [2,1-*b*]-1,3,4-thiadiazoles (Scheme 33). The reported synthetic method seems to be useful in terms of reduced time for synthesis, higher rate of reaction, and greater % yields.

**Scheme 34:**
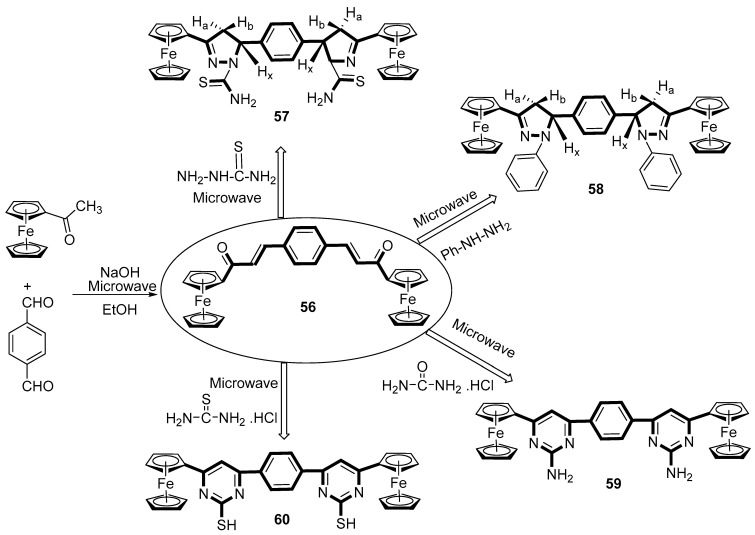
Microwave-supported synthesis of ferrocenyl bis-pyrazolines and bis-pyrimidine derivatives.

Khan et al. reported microwave-assisted synthesis of macromolecules containing ferrocenyl bis-pyrazoline (**57**, **58**) and bis-pyrimidine (**59**, **60**) derivatives by treating ferrocenyl bis-chalcone **56** with thiosemicarbazide, phenylhydrazine, guanidine hydrochloride, and thiourea (Scheme 34). Targeted compounds were further characterized by IR, ^1^H NMR, ^13^C NMR, mass spectrometry, and combustion analysis [60].

**Scheme 35:**
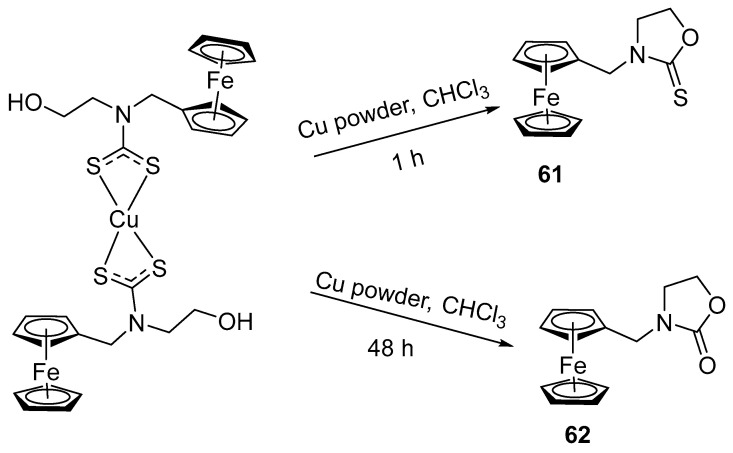
Synthesis of ferrocene-based oxazolidine-2-thione and ferrocene-based oxazolidine.

Different reaction conditions afforded the formation of two different products with different yields, characterized by X-ray crystallography and spectroscopic analysis (Scheme 35).

### 2.9. Synthesis of Ferrocene-Fused Cyclic Derivatives

**Scheme 36:**
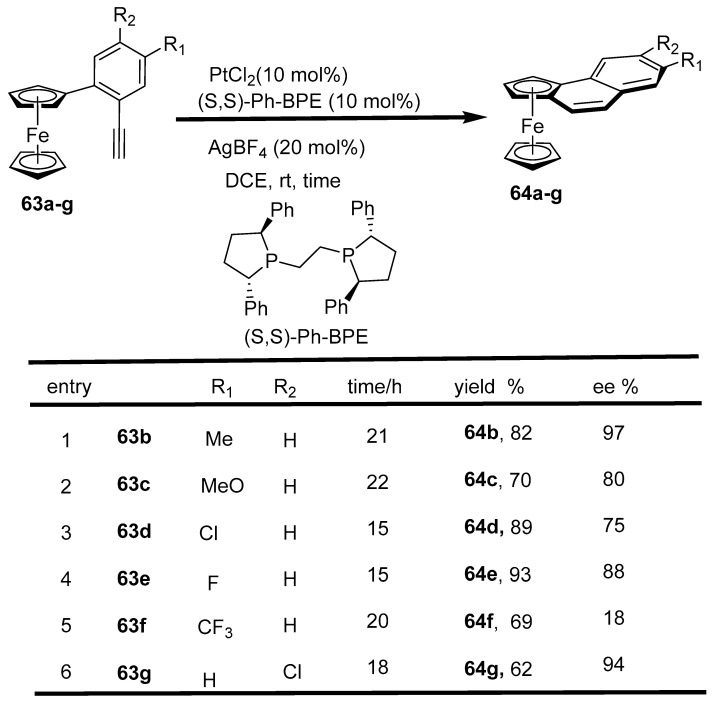
Asymmetric synthesis of ferrocene-fused aromatic compounds.

Shibata and co-workers explored platinum chloride as a useful catalyst for enantioselective synthesis of a ferrocene-fused aromatic system through cycloisomerization of 2-ethynyl-1 ferrocenyl benzene precursor (Scheme 36). The mechanistic study revealed that C–H activation did not occur in the rate-determining step and the reaction proceeded through the activation of the alkyne nucleus via protonation of π cloud of chiral Pt complex followed by a 6-endo-dig cyclization reaction [61].

**Scheme 37:**
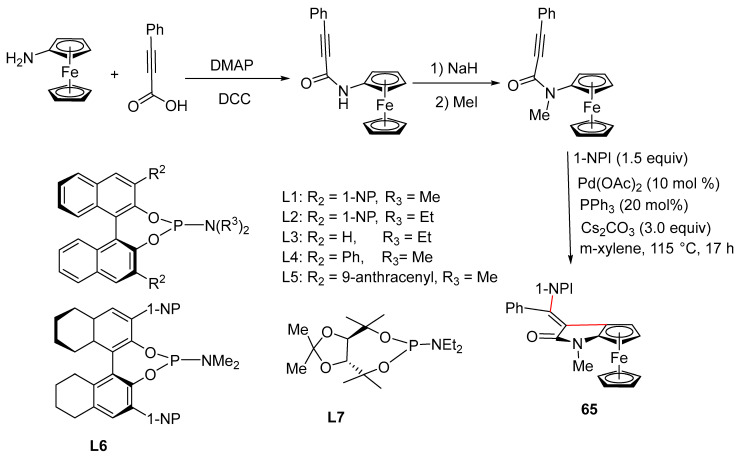
Synthesis of ferrocene [1,2-*d*] pyrrolinones.

Recently, a new protocol has been safely scaled up based on Pd-catalyzed intramolecular tandem cyclization of ferrocene analogs with substituted alkyne, to form novel asymmetric planer ferrocene [1,2-*d*] pyrrolidines [62]. This Pd (0)-catalyzed tandem intermolecular syn-carbopalladation of N-ferrocenyl propionamide with aryl iodides had been appreciated, fabricating planar asymmetric ferrocene [1,2-*d*] pyrrolinones in virtuous yields. Moreover, using BINOL-based phosphoramidite ligands, up to 95% ee was attained (Scheme 37).

**Scheme 38:**
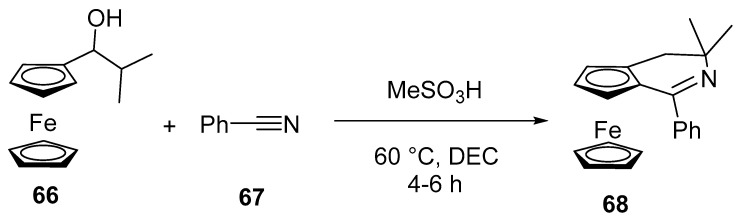
Synthesis of dihydro ferrocenyl pyridine derivatives.

The Ritter reaction was used for the preparation of 3,4-dihydroferrocenyl[c]pyridine racemates by Rozhkova et al. [63]. In this newly planned strategy, 2-methyl-1-ferrocenyl propane-1-ol was treated with substituted nitriles in the existence of a methyl sulfonic acid catalyst (Scheme 38). The targeted racemates were successfully resolved by preparatory HPLC on a chiral cell OD-H column and the absolute configuration of each enantiomer was determined by X-ray crystallographic studies.

## 3. Applications of Ferrocene Derivatives

The application of metallocene-functionalized compounds has become a research topic of growing interest in recent years. Ferrocenes have been used as chemical intermediates, antiknock additives to gasoline, lubricants, and for other uses, but a primary application is related to catalysis [64]. Ferrocene is also currently being investigated as an active pharmacological agent against many notorious diseases like cancer, malaria, and other bacterial infections. Here, we tried to review applications of ferrocene derivatives embracing several fields like materials science, industry, agriculture, biology, medicine, electrochemistry, etc.

### 3.1. Materials Science Applications

Ferrocene retains an iron synthetic chemistry which includes a 3D structure that permits the synthesis of many derivatives, outstanding thermal stabilities [65,66], and rapid dissolution in common organic solvents [67]. Thanks to these imperative features, ferrocene derivatives have been used in a wide number of materials science applications; so diverse are they that it is problematic to classify these into a coherent manuscript. Thus, this is a general outline of some specific applications of ferrocene-functionalized compounds, including NLO devices, functional materials in electrochemical sensors, electron beam lithography, and electrochromic applications.

#### 3.1.1. Biosensing Potential of Saccharides

**Scheme 39:**
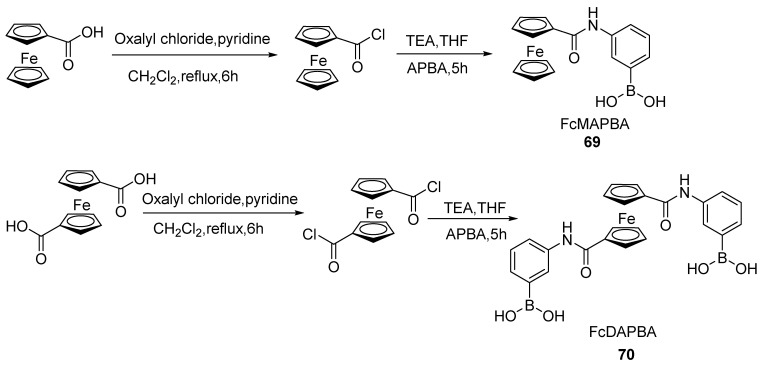
Total synthesis of ferrocene-based aminophenyl and diaminophenyl boronic acid.

By considering the binding affinities, biocompatibility, and host–guest molecule recognition capacity of amides, Saleem et al. synthesized ferrocene-appended phenylboronic amino acid and diaminophenyl boronic acid (APBA) derivatives and evaluated their saccharide sensing activity using spectroscopic analysis (Scheme 39). The neighboring environment of the boronic acid binding site and the linking ferrocene unit enhanced the binding abilities of tetrahedral boron. Further, ferrocene’s electrochemical response signified a substantial step towards effective and robust artificial bioelectronic affinity receptors [68].

**Figure 1:**
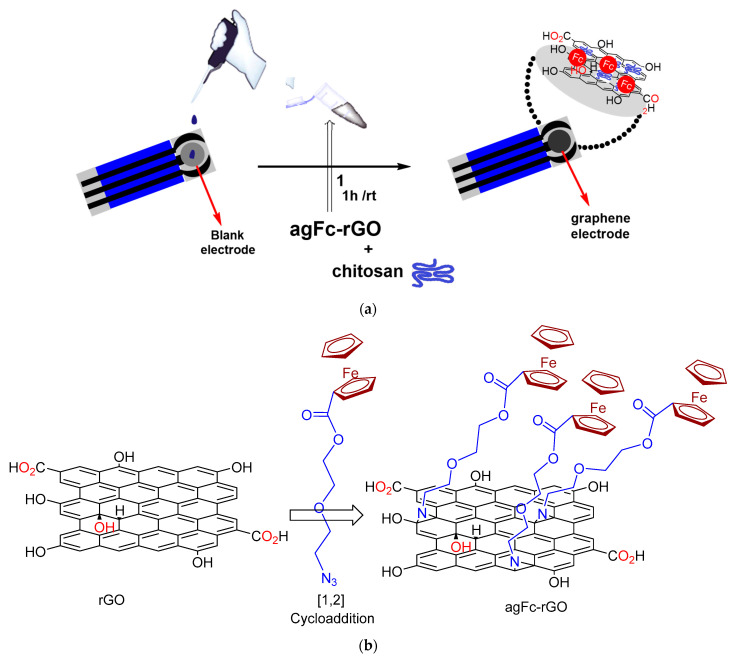
(**a**) Ferrocene-grafted (rGO) electrode preparation by drop-casting ferrocene-based graphene (**b**) Synthesis of ω–azidodiethylene-glycol–α–ferrocenyl esters (agFc) and rGO nanosheets from agFc–rGO nanosheets through cycloaddition reaction.

In a different work, Rabti and co-authors designed an innovative ferrocene-grafted (rGO) electrode, prepared by drop-casting ferrocene-based graphene against a polyester surface (Figure 1a). The freshly synthesized ω–azidodiethylene-glycol–α–ferrocenyl esters (agFc) and rGO nanosheets generated extremely aqueous soluble agFc–rGO nanosheets through cycloaddition (Figure 1b). Further, biological interactions with glucose oxidase for glucose recognition were accomplished using bioelectrode, offering evidence for latent biosensing use [69].

#### 3.1.2. Anion Biosensors

**Figure 2:**
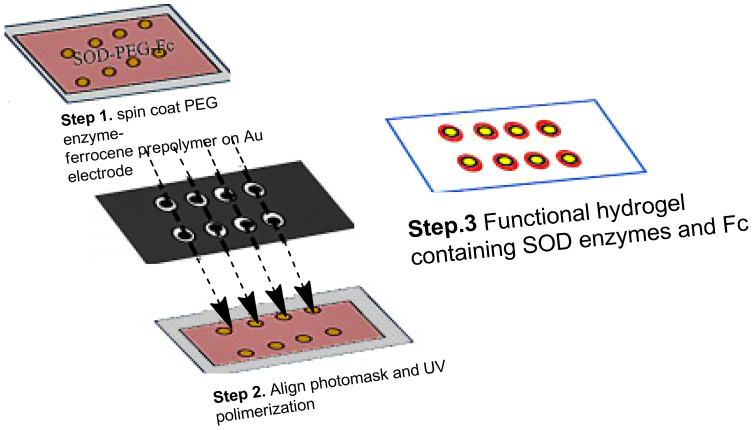
Biosensing chip comprising eight micropatterned gold electrodes.

Various analytical methods were reported in the literature for the detection of superoxide anion, but all fail to provide in vivo and in vitro monitoring. These issues were resolved by fabricating highly selective hydrogel structures in which ferrocene units were associated with superoxide dismutase into a poly diacrylate hydrogen matrix for the synthesis of SOD-Fc-PEG-based electrodes [68]. The polymeric solution was emulated by optical lithography in Au microelectrodes assembled onto the surface of glass slides (Figure 2). Such reproducible biosensing materials were used for the diagnosis of carcinoma anion cells that release superoxide anions, with LOD of 0.001 μm and sensitivity of 1.41 Na 4 M/mm^2^.

#### 3.1.3. Hydrogen Peroxide Biosensor

**Figure 3:**
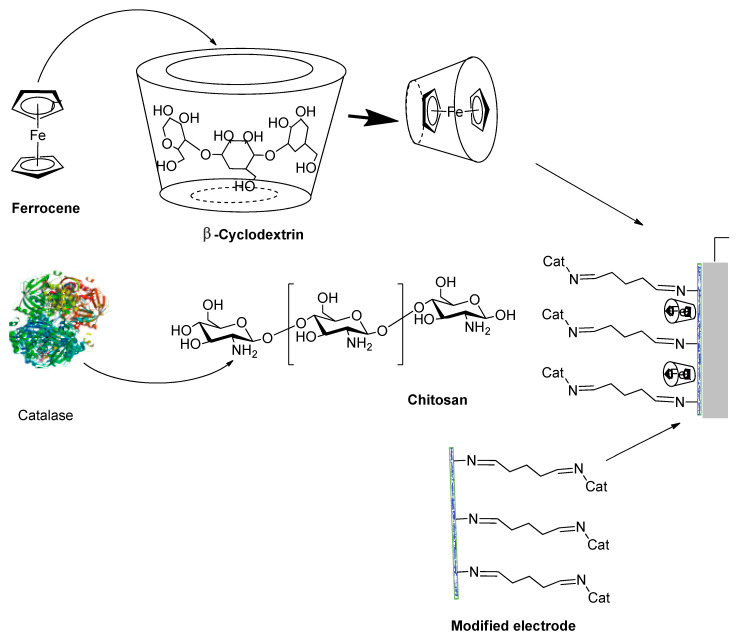
Schematic illustration of the CTS-CAT/β-CD-FE amalgamated electrode.

In a providential work, Dong’s group developed an electrochemical biosensor based on chitosan-immobilized enzymes and a β-cyclodextrin-containing ferrocene complex for the biological detection of H_2_O_2_ [70]. A CTS-CAT/β-CD-FE-grafted electrode was constructed by conglomerating CTS-CAT with β-CD-Fe complex onto the surface of a glassy carbon electrode and used for the electrochemical evaluation of hydrogen peroxide (Figure 3). Further, the inclusion of ferrocene units offered great durability to the β-cyclodextrin complex.

#### 3.1.4. Peptide Biosensors

**Figure 4:**
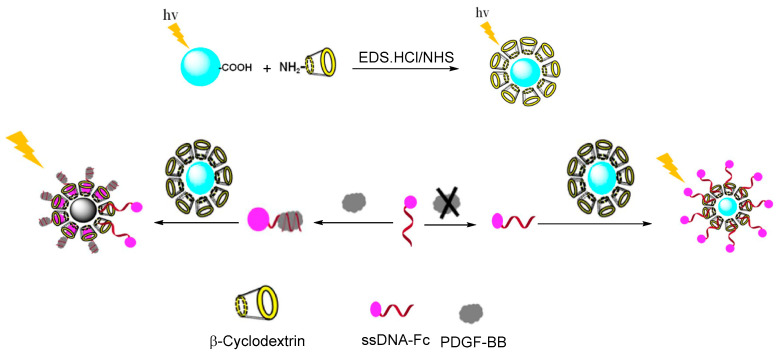
Sensing procedure of the proposed β-CD-CD fluorescent nanoprobe for PDGF-BB detection.

Xia Lu and co-workers invented a new fluorescent nano-bioaptamer sensor assimilating a β-cyclodextrin-modified quantum dot luminescent probe and applied it to find the (PDGF)-BB factor that showed significant fluorescence quenching (Figure 4). The reason for this quenching phenomenon was initiated by the Fc unit that recognized the host–guest interaction, followed by the blockage of photoexcited electron transport between Fc and CDs supported by DNA that reduced fluorescence. This ferrocene-marked adapter served as a sensitive and selective biological receptor for detecting platelet-derived growth factor BB [71].

#### 3.1.5. Chemosensing Applications

**Figure 5:**
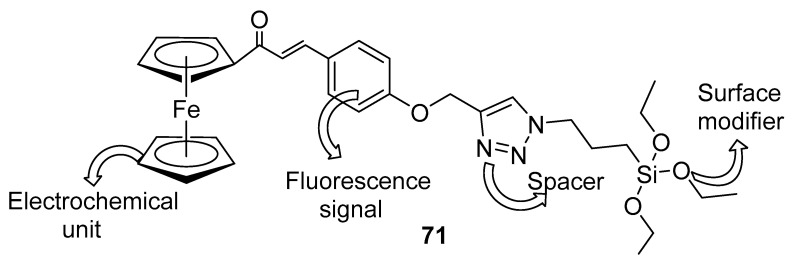
Molecular design of the chemosensor.

The astonishing stability, easy functionalization, and distinctive redox behavior of the ferrocenyl nucleus have led to expeditious research in molecular recognition studies. Recently, Preet and Arora with their colleagues combined the electrochemical activity of ferrocene with the chemical ligation potential of organosilanes via the preparation of ferrocene-tailed, chalcone-based organosilanes from ethynylferrocene precursor (**71**) using click chemistry [72]. The resultant ligands were examined for their ion sensing potential and exhibited their sensing ability for Cu^+2^ ion on the addition of EDTA with the reversal of the sensing phenomenon. Moreover, the presence of the ferrocene nucleus offered a diverse signaling sensor that was helpful in the naked-eye revelation of Cu^+1^ ion within a limit of detection of 10 µm (Figure 5).

**Figure 6:**
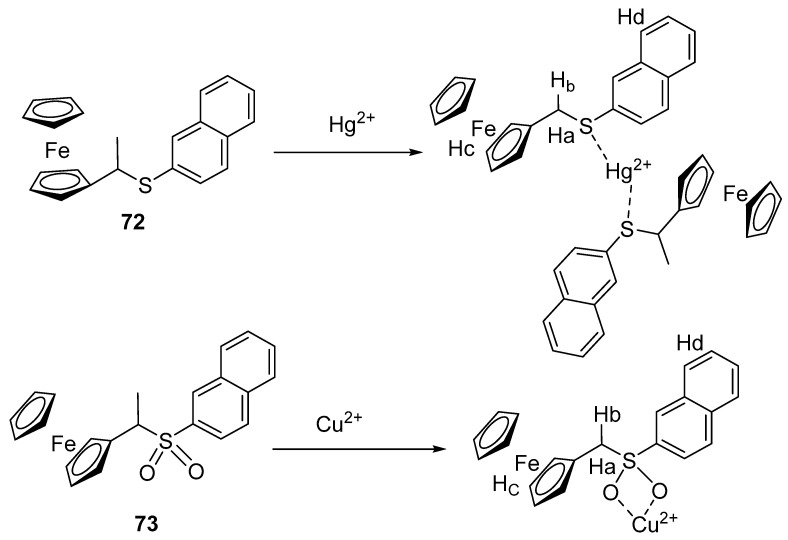
Possible binding mode of Hg^2+^ and Cu^1+^ ions with the receptors.

Another research group constructed two highly selective ferrocene-based chemosensing materials without the inclusion of nitrogen atoms for the chemoselective detection of Cu^2+^ and Hg^2+^ ions [73]. Compound **72** showed remarkable detection aptitude for Hg^+^ via optical and electrochemical signals with a lower value of detection limit that further confirmed its sensor potential. Further, ferrocene-functionalized sulfone **73** also demonstrated highly selective detection toward Cu^2+^ with the detection limit approaching 5.22 × 10^−7^ M (Figure 6).

**Figure 7:**
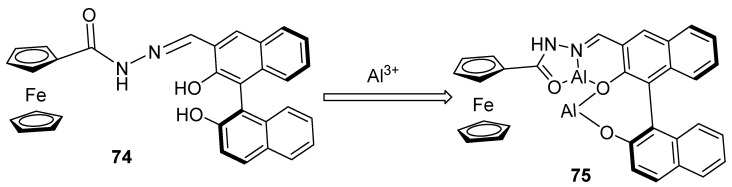
Proposed metal complex and its molecular model.

In a more recent work, a ferrocene-grafted chemosensor was proposed and assessed for its selectivity in the detection of Al^3+^ cations [74]. Mechanistic study revealed that the probe compound captured the Al^+3^ and enhanced the fluorescence intensity 200 times. Various characterization techniques confirmed the formation of a complex between compound **74** and Al^3+^, and were successfully used to find out the Al^3+^ content in environmental samples (Figure 7).

**Figure 8:**
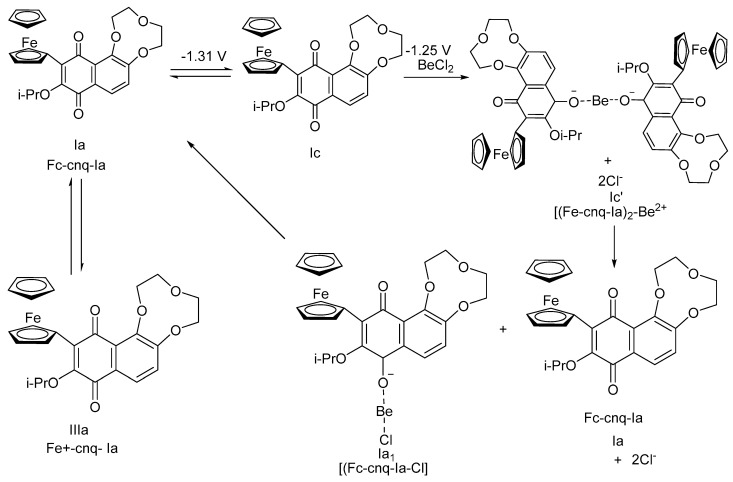
Proposed mechanism for ion pair formation between Be^2+^ ion and Fc−cnq−1a.

Finally, the method employed by Atasen explained the sensing potential of Be^2+^ by ion pair formation with ferrocenyl naphthoquinones radicals and dianions [75]. Cyclo-voltammetric analysis revealed that the sensitivity effect was more pronounced in the case of Be^2+^ among all alkaline earth metals that formed an ion pair with Fc–cnq–Ia and could selectively sense the ultra-trace amount of Be^2+^ (LOD 3.6 ppb). The proposed mechanism of the ion pair formation reaction between Be^2+^ ion and Fc–cnq–1a is depicted in Figure 8.

**Figure 9:**
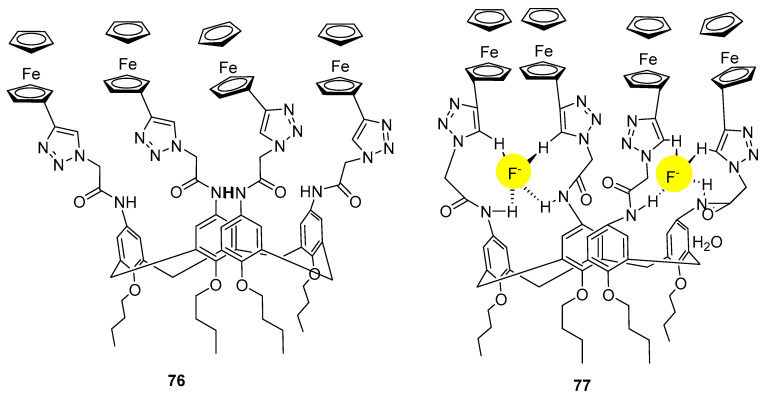
Structure of the synthesized receptor and its proposed binding mode with F^−^.

Anion recognition and anion sensing are of special interest not only because of their significant roles in biological systems but also because they can work in both organic and aqueous media. The group of Hosseinzadeh and Maliji recently reported the synthesis of an innovative tetra ferrocene-triazole-amide-based Calix [4] arene and described it as an efficient optical and electrochemical anion sensor [76]. CV and proton nuclear magnetic resonance titration tests disclosed the selective and ultrasensitive behavior of compound **79** for F^−^ with a detection limit of 2.98 µM. The above-reported outcomes could be open-and-shut proof of the efficiency of a newly synthesized compound as a sensitive moiety for F^−^ anion (Figure 9).

**Figure 10:**
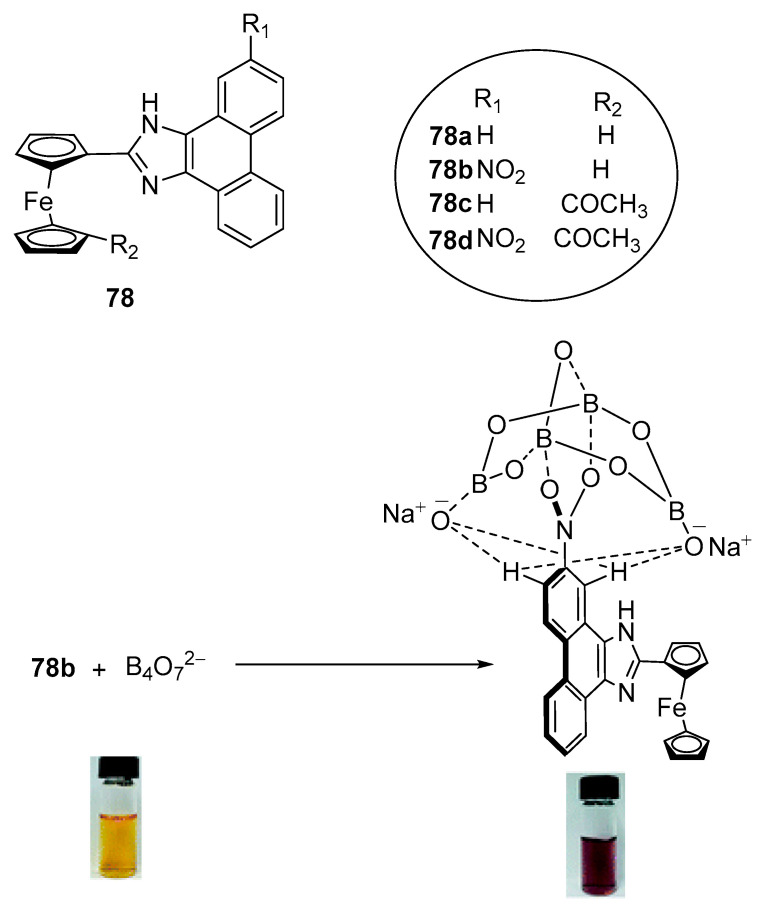
Structure and mode of complexation of synthesized receptor **78b**.

Wu and co-workers designed four phenanthroimidazole derivatives with ferrocene scaffolds and examined their anion recognition properties towards twelve common anions such as F^−^, Cl^−^, Br^−^, SO_4_^2−^, OAc^−^, etc. [77]. Investigation of these common anions via spectroscope studies indicated that compounds 7**8b** and 7**8d** sensed B_4_O_7_^−2^ specifically and the sensing potential was realized through the naked eye, ^1^H NMR, and various titration experiments, concluding that the binding force between B_4_O_7_^−2^ and 7**8b** was hydrogen bonding (Figure 10).

**Figure 11:**
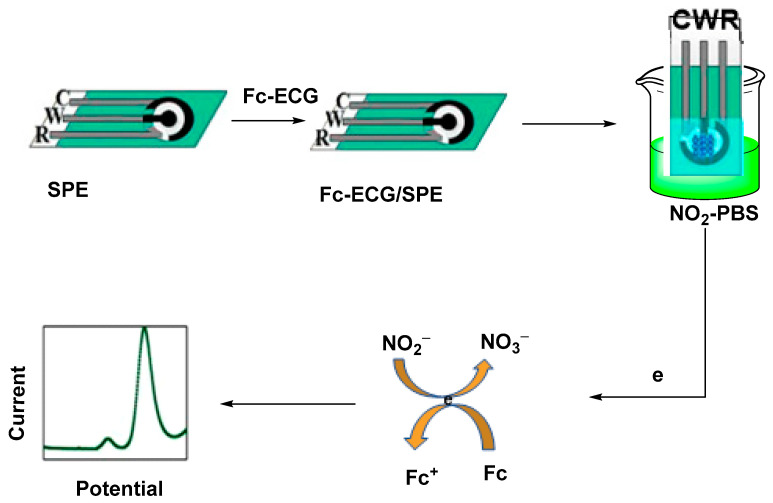
Assembly of Fc-ECG electrochemical sensor for nitrite recognition by DPV.

Another interesting example was reported in Feng’s publication where the author designed a degradable electrochemical sensing system for the determination of nitrite moiety in pickle juice [78]. Nitrite is a commonly used food preservative, but a side effect was also reported as it reacted with amino acids and produced a carcinogenic effect [79]. A carbon electrode was developed by installing ferrocenyl derivatives on the surface of screen-printed electrodes; characterization techniques realized that the sensitivity and stability of the reported sensor were brilliant, with R.S.D lower than 2.1% (Figure 11).

**Figure 12:**
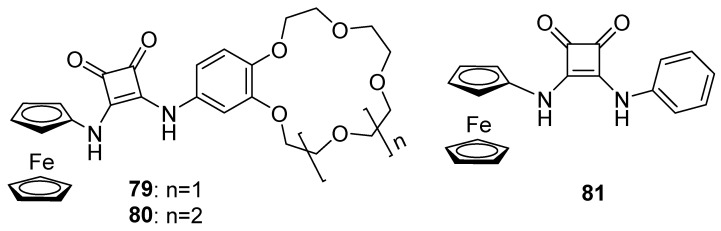
Structures of new ion pair sensors based on squaramide scaffold.

In an advanced work, Zaleskaya and co-authors fabricated ion pair sensing materials, having benzo crown ether and ferrocene-functionalized squaramide as the cation accepting center and anion welcoming center [80]. Receptors **79** and **80** were observed to be stronger candidates that could sense negative ions more efficiently in the company of positively charged species. This research offered a huge platform for the measurement of salt content in a real-life situation in which an appropriate amount of salt is required (Figure 12).

#### 3.1.6. Electron Beam Lithographic Application

**Figure 13:**
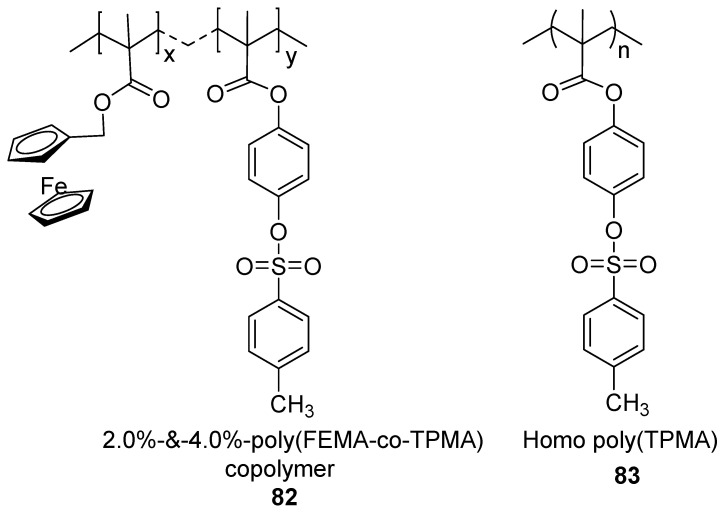
Chemical structures of ferrocene-bearing non-ionic poly-aryl tosylates.

Ferrocene-grafted organic–inorganic hybrids are an innovation category of homo polymeric resists, and the recently synthesized poly-(TPMA) and poly-(FEMA-CO-TPMA) were studied for their large-scale micro/nanolithographic functions [81]. These copolymers also behaved like light resists when exposed to an electron beam lithography device for arranging nano-marked characters. The ultra-sensitivity and contrast of 2% poly (FEMA)-CO-TPMA **82** resulted from ferrocene’s inclusion, which elevated the sensitivity and resolution scale of the polymer in comparison to **83** (Figure 13).

#### 3.1.7. Nonlinear Optics Applications of Ferrocene-Based Conjugates

**Scheme 40:**
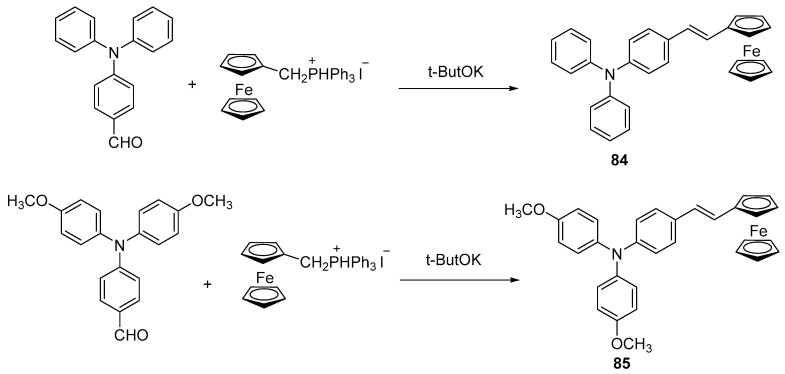
Synthesis of ferrocene-based conjugates with third-order nonlinear optical (NLO) properties.

Relying on the merits of triphenylamine and ferrocenes as building blocks, novel compounds based on N, N-diphenyl-4-E-[(1-ferrocenyl)ethenyl] aniline (**84**) and N, N-diethoxyphenyl-4-E-[(1-ferrocenyl)ethenyl] aniline (**85**) were designed and synthesized, and a detailed study of these organometallic analogs revealed their NLO potential (Scheme 40). The cyclic voltammogram and ON/OFF aperture Z-scan measurements confirmed the third-order nonlinear optical (NLO) properties of these reported compounds, considered to be the result of their extended conjugation system [82]. Further, optically switching properties of these derivatives were also examined, which further confirmed their novelty.

**Figure 14:**
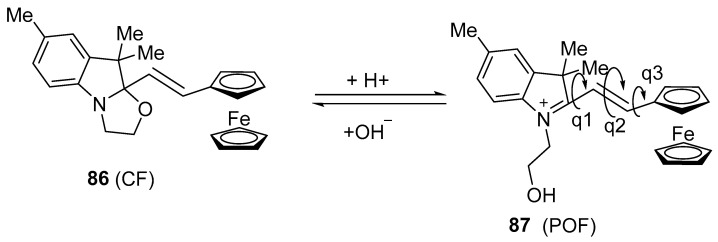
Structure of the closed and protonated open forms of the IND-Fc derivative.

In the same vein, Pielak’s group reported the first- and second-order nonlinear optical properties of ferrocene-linked indolino-oxazolidine derivatives through scattering techniques, which showed that the β factor for NLO properties and β-POF/β-CF value was large enough for devices used daily [83]. Quantum study also revealed that the β value of the protonated “ON” form emerged from two lower-energy excited states, which partially diminished their effect due to the counter-direction of light-stimulated charge transfer (Figure 14).

#### 3.1.8. Solar Cell Applications

**Figure 15:**
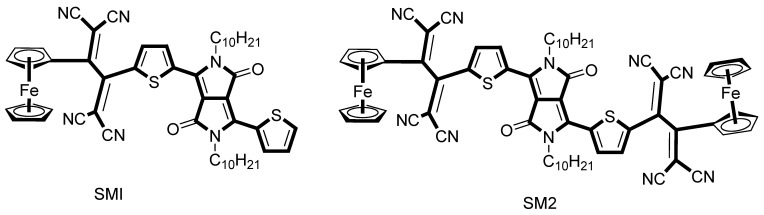
Chemical structures of **SM_1_** and **SM_2_**.

Ferrocene-linked tetracyano butadiene derivatives of diketopyrrolopyrroles **SM_1_** and **SM_2_** were extensively studied for their photovoltaic features and reported with maximum power conversion efficiency for solar cell devices (Figure 15). This energy conversion potential of **SM_2_** was remarkable when compared with **SM_1_** because of the higher value of short-circuit current (Jsc) and fill factor (FF). Therefore, the ferrocenethiophene donor unit offered a remarkable non-fullerene acceptor and presented chief proficiency in the advancement of non-fullerene organic solar cells [83].

**Scheme 41:**
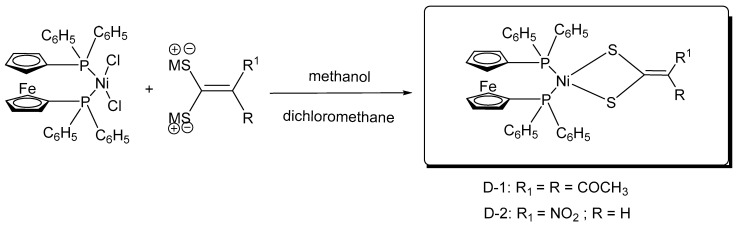
Synthetic route for the preparation of sensitizers.

The newly synthesized heterometallic compounds, designed for solar cell applications, were two ferrocene-containing Ni-bithiolate complexes, D-1 and D-2, functionalized with 2,2-diacetyl and 2-NO_2_ linker, respectively, reported in a more advanced work of Amit and co-workers (Scheme 41). These synthesized ligands exhibited excellent donor sensitization potential, examined by light-driven spectral analysis. The photovoltaic index of the synthesized materials was not so absurd but the inclusion of TiO_2_ nanoparticles enhanced the level of charge-reversing ability that increased the sensitization to the compromising stage [84].

**Figure 16:**
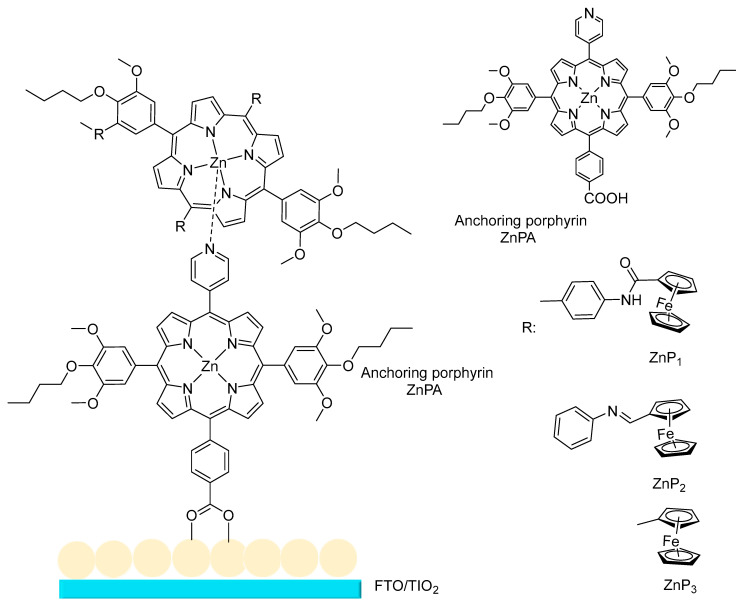
The complete aggregation method of accumulation on TiO_2_ electrode surface.

Incorporating ferrocenyl groups into meso-substituted porphyrins with various donor substituents affords three metallo ligands: ZnP_1_, ZnP_2_, and ZnP_3_ [85]. Self-aggregated zinc porphyrins and a linker, ZnPA, were utilized as sensitizers in dye-sensitized solar cells. It was observed that the photocurrent transformation potential of self-aggregated units was high; particularly, an amide bridge and ferrocene-based zinc porphyrin (ZnP1) solar cell exhibited maximum photocurrent turning capacity compared with its derivatives (Figure 16).

#### 3.1.9. Supercapacitor Applications

**Figure 17:**
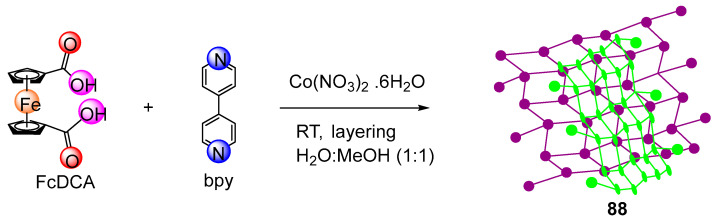
Synthesis of cobalt-based inclined polycatenated MOF.

An advanced cobalt-graded poly-catenated metal–organic framework was constructed by Rajak’s group and its electrochemical properties were extensively studied [86]. The synthesized framework (**88**) was applied with a binder to modify a glassy carbon electrode and evaluated its supercapacitor performance with or without a binder group. Detailed analysis was performed with a binder containing a modified glassy carbon electrode, which exhibited the superb value of capacitance and stability of that metal–organic framework onto the surface of the electrodes (Figure 17).

**Figure 18:**
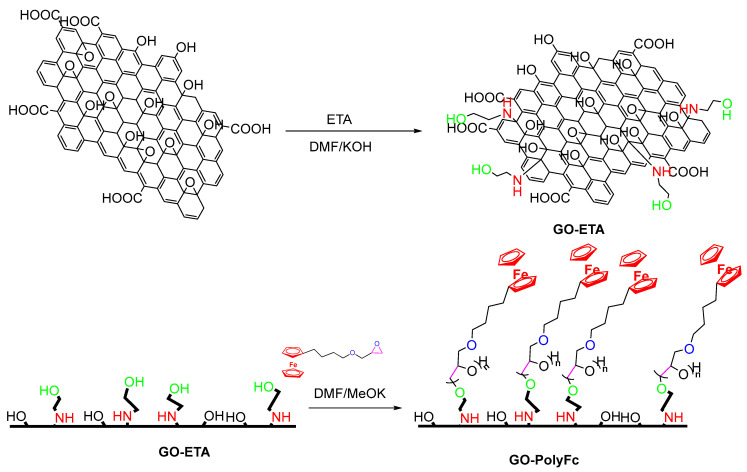
Synthesis of graphene oxide surface-modified ferrocene nanosheets (GO-polyFc).

Teimuri et al. developed a typical supercapacitor hinged on a glassy carbon electrode, mutated with graphene-oxide-loaded ferrocene through ring-opening polymerization (Figure 18). The amalgamated polyFc was impaired on glassy carbon, and its electrochemical potential was noticed through CV, GCD, and EIS techniques. All these characterization techniques revealed that the modified material showed excellent electrochemical features as a supercapacitor electrode with a high capacitance value of 200 Fg^−1^ [87].

#### 3.1.10. Uses of Ferrocene in Modern Fuel Cells or Batteries

**Figure 19:**
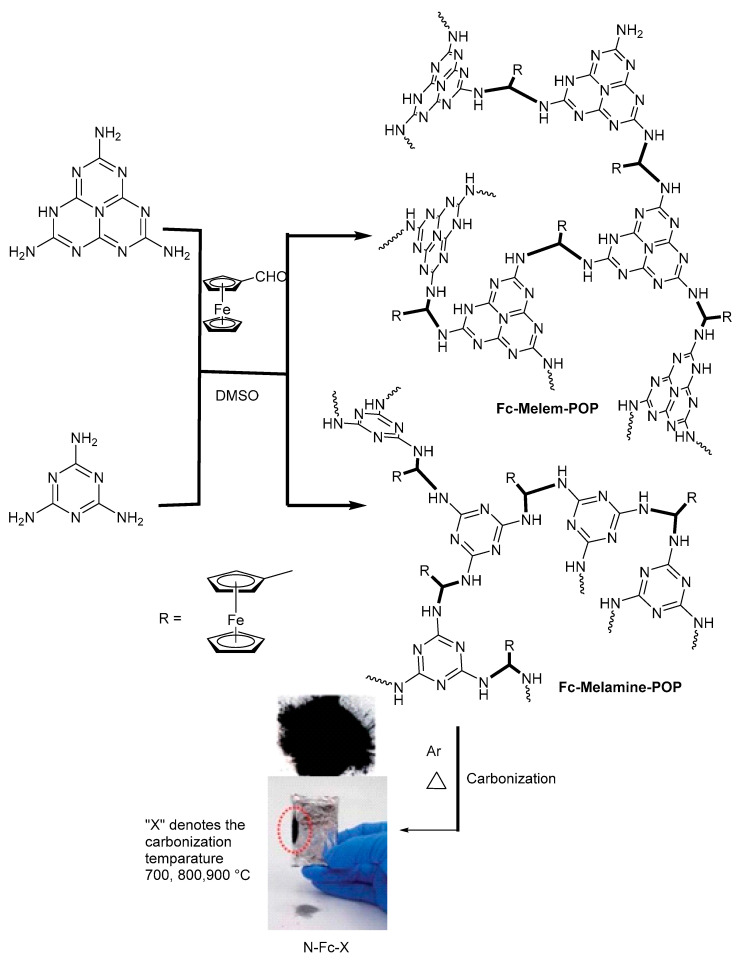
Synthesis of ferrocene-functionalized POPs and nitrogen-doped carbon catalysts.

Ferrocene as an organometallic compound has been investigated as a standard electrode in redox potential measurements because of its excellent redox activity. In this study, the redox activity of ferrocene was utilized to design two ferrocene-functionalized polymers through cheap Schiff base interaction with melamine as the backbone [88]. Destructive distillation of synthesized material produced nano hybrids along with the Fe_3_C/Fe moiety. Further, a rechargeable zinc–air battery was constructed by utilizing Fe_3_C/Fe as a cathode catalyst which exhibited high power density, a small charge–discharge gap, and remarkable stability with less activity decay, in comparison to Pt/C containing commercial batteries (Figure 19).

Liu et al. introduced an efficient ferrocene-based electrode for Li^1+^ batteries with excellent electrochemical features such as significant reversible power, high charge retention, and remarkable charge–discharge rate capability [89]. XRD and cyclic voltammetry analysis showed that the electrochemical reaction of ferrocene was partially reversible and some metallic iron (Fe) clung to the anode even after de-lithiation. The metallic iron and solid electrolyte interface were amenable to the superb electrochemical properties of the designed electrode.

#### 3.1.11. Redox-Mediating Potential of Ferrocene Derivatives

**Figure 20:**
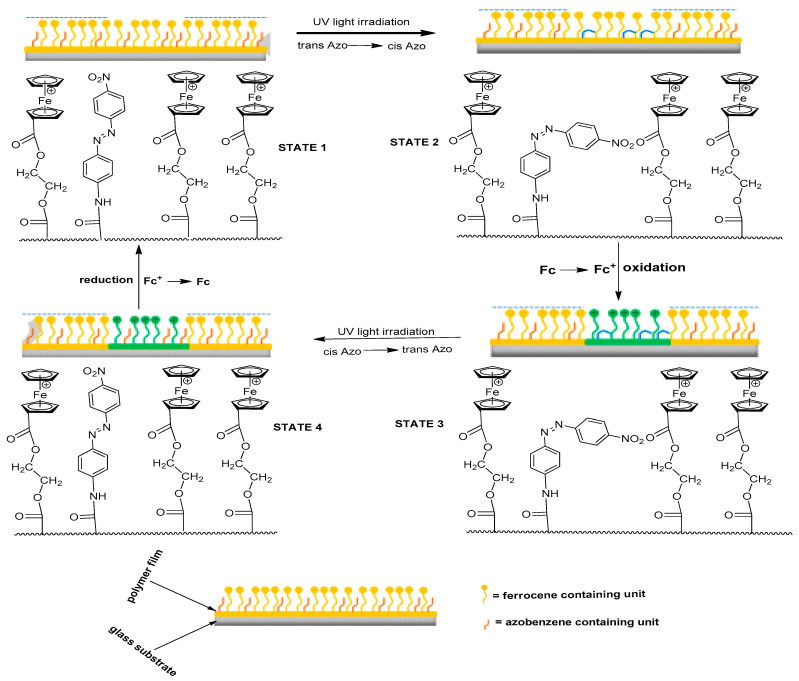
Mechanism of multi-state information storage on (P (FCEMA-MAAZoo)s)-based polymers.

Among redox-active materials, ferrocene and ferrocene-based polymeric materials are of special interest due to their high stability in redox reactions and facile derivative syntheses. For example, an array of redox poly-(2-methaarcryloyloxy) and light-sensitive polymers based on ferrocenyl carboxidate-Co-4-methylacryloamino-4-nitroazobenzene (P (F_C_EMA-MAAZoo)s) were synthesized through free radical oligomerization protocol [90]. Light-responsive potential and redox properties of these polymers were evaluated via spectroscopy and cyclovoltammetric analysis. These polymeric slides were thought to have vital applications in a potentially high-information storehouse, and their mechanism of information storage is described in Figure 20.

#### 3.1.12. Application in Photo-Induced Electron Transfer: Cascaded Molecular Logic

**Figure 21:**
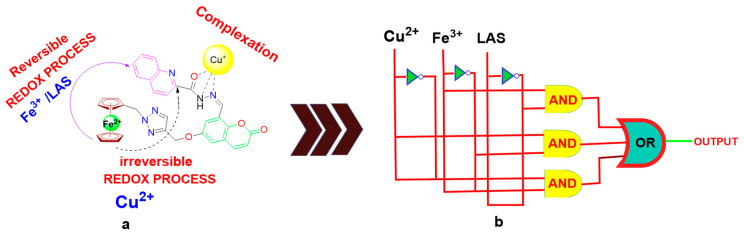
(**a**) Ferrocene-appended coumarin–quinoline probe. (**b**) Combinational logic constructed with Cu^2+^, Fe^3+^, and LAS as inputs.

Karmakar and co-workers combined the redox activity of ferrocene with the photophysical properties of coumarin and quinoline through the development of a molecular tool that turned on luminescence through the photo-induced electron transfer (PET) phenomenon with d^10^ metal ions [91]. Further, this research group also fabricated a prob (a), which behaved differently with various metal ions through fluorescent expression. Taking the edge of the differential selectiveness of this probe, binary basic logic circuits “INHIBII” and “OR” were designed, which were used in biosensing applications with slight modifications (Figure 21).

#### 3.1.13. Role of Ferrocene in Catalytic Graphitization

**Figure 22:**
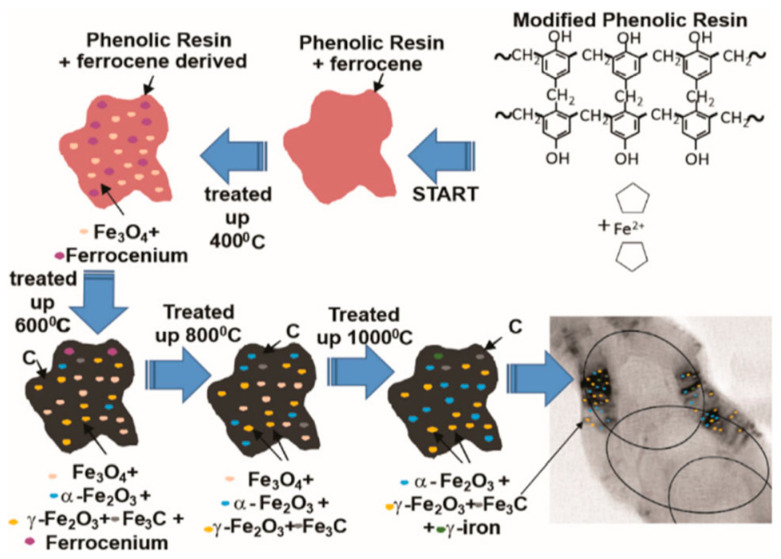
Magnetic nanoparticle formation during phenolic resin catalytic graphitization/ferrocene thermal decomposition process.

In laboratory-prepared, modified novolak phenolic resins, graphitization was induced using ferrocene (PR). A recent study described the morphological and structural differences in the intermediate carbon structures containing iron throughout the graphitization process, obtained after several steps of heat treatment from 200 to 1000 °C. The presence of ferrocene (a catalyst and carbon source) and the reducing environment are both responsible for the induction of phenolic resin graphitization in onion-like hollow carbon. The hollow onion-shaped material facilitated 96% elimination in 5 min in the photocatalytic research of the pesticide atrazine (ATZ) in an aqueous medium and affected the mechanism of ATZ degradation and the synthesis of atrazine-2-hydroxy (HAT) in various reactors (Figure 22) [92].

#### 3.1.14. Ferrocene-Based Polymer as a Scavenger for Radioiodine

**Scheme 42:**
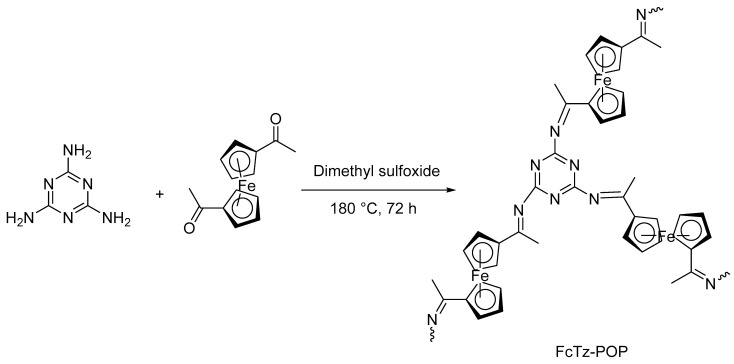
Synthesis of ferrocene-linked organic polymer (FcTz-POP) for iodine removal.

By using the Schiff base reaction, the porous ferrocene-linked organic polymer (FcTz-POP) was synthesized and exhibited a high iodine vapor capacity, 1.8 times that of a reference material without ferrocene (BpTz-POP). FcTz-POP exhibits a rapid rate of I_2_ adsorption and a relatively high affinity due to the abundance of ferrocene blocks with a high electron density (Scheme 42). The iodine capturing for FcTzPOP, which is substantially higher than that of ferrocene-free BpTz-POP, can reach up to 396 wt% at 348 K and atmospheric pressure by integrating ferrocene building blocks. This is mostly due to the cyclopentadienyl group, which interacts with the iodine molecule more strongly than the benzene ring, and the Fe^2+^ on ferrocene, which may electrostatically bind negatively charged polyiodides [93].

#### 3.1.15. Ferrocene-Based Hyperbranched Polymers as Electroactive Materials

**Scheme 43:**
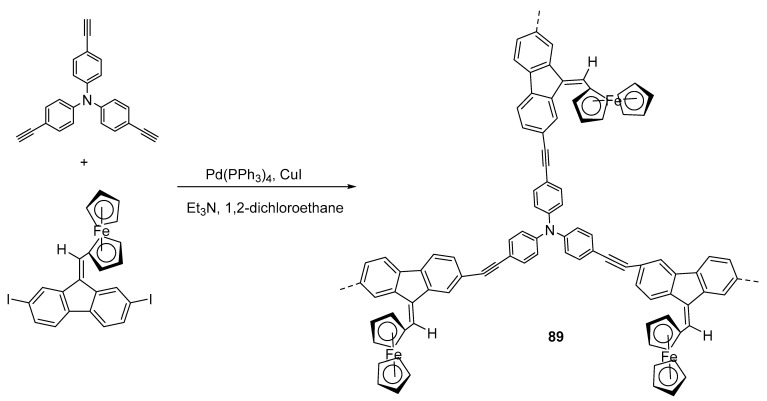
Ferrocene–fluorene-appended branched polymer.

Using the Sonogashira coupling reaction, Wei et al. developed a ferrocene-based hyperbranched metallopolymer (**89**), incorporating the alternate units of TPA and fluorene as the main skeleton. An efficient method of controlling their morphology was successfully demonstrated to produce uniform nanostructures as spheres and hollow polyhedrons for specific applications (Scheme 43). When built into laser-induced breakdown spectroscopy (LIBs), the spherical polymer (SP) with a microporous structure had remarkable redox performances and displayed high capacity, large cycle stability, and better rate capability. The analogous HP provided rising BET surface areas of 1195 m^2^ g^−1^ in the precursor-derived ceramics by merging the intrinsic micropore of the organic frameworks and the macropore from the hollow polyhedron, and the as-made carbon materials were further applicable to remove water [94].

### 3.2. Applications in Catalysis

Because of their accessibility, novel stereochemical characteristics, the extensive variability of co-ordination approaches and options for the modification of the steric hindrance, thermal stability and tolerance to oxygen and water, serene derivatization, and electronic properties, ferrocene-grafted ligands created a large number of multipurpose ligand architectures in the recent trend of asymmetric catalysis.

#### 3.2.1. Ferrocene-Functionalized Phosphine Ligand

**Figure 23:**
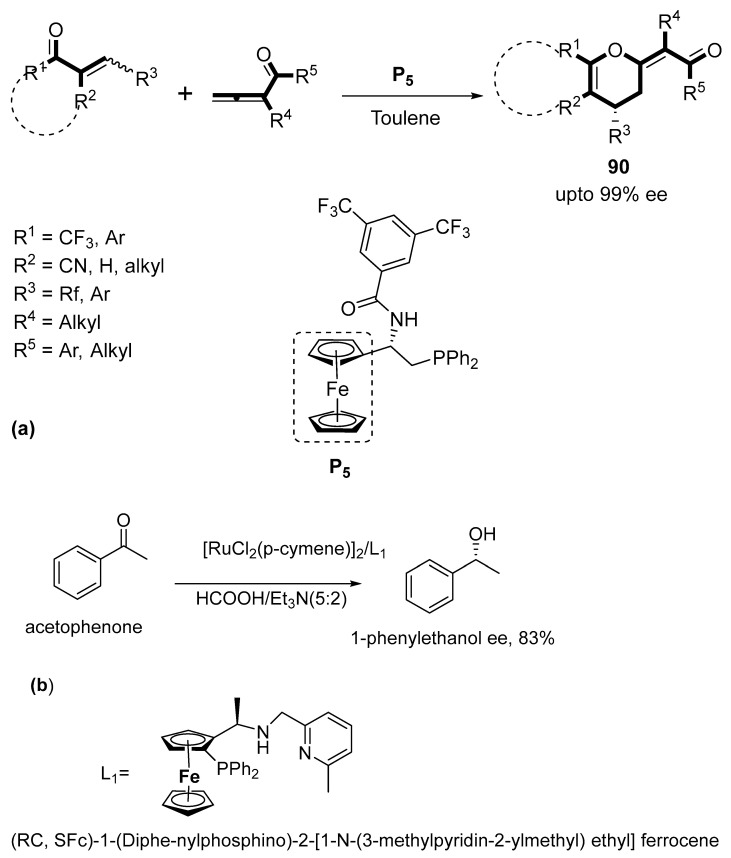
(**a**) Phosphine-catalyzed cycloaddition reactions of α-substituted allenes, (**b**) asymmetric synthesis of 1-phenylethanol (ee) from acetophenone using ferrocenyl chiral ligand.

The chemistry of the di- and polyphosphine ferrocene-based ligands in catalytic cross-coupling is detailed in carbon–carbon, carbon–nitrogen, and carbon–oxygen bond-forming reactions. Recently, an efficient ferrocene-grafted bi-functional phosphine-catalyzed enantioselective cycloaddition reaction of oxo-allenes with a large number of ketones was reported in order to prepare substituted dihydropyran derivative (**90**) [95]. Mechanistic study revealed that multifunctional phosphine catalyst containing ferrocene moiety (**P_5_**), had excellent stereoselectivity in Rauhut Currier reaction, allylation, and chiral cycloaddition reaction, among all other ferrocene-functionalized phosphine ligands (Figure 23a). Because of ferrocene’s strong electron-donating nature as well as its distinctive structure, chiral compounds based on ferrocene played important roles in asymmetric processes. Today, a wide range of chiral ferrocenyl ligands are known. Ferrocenyl chiral ligands have been exercised for ruthenium (II)-catalyzed asymmetric transfer hydrogenation of acetophenone using HCOOH/Et_3_N azeotrope as the hydrogen source. When (RC, SFc)-1-(Diphenylphosphino)-2-[1-N-(3-methylpyridin-2-ylmethyl) ethyl] ferrocene (L1) was employed, a moderate chemical yield of 1-phenylethanol with 83% ee was produced (Figure 23b) [96].

#### 3.2.2. Ferrocene-Appended Thiazolidine Ligand Framework

**Figure 24:**
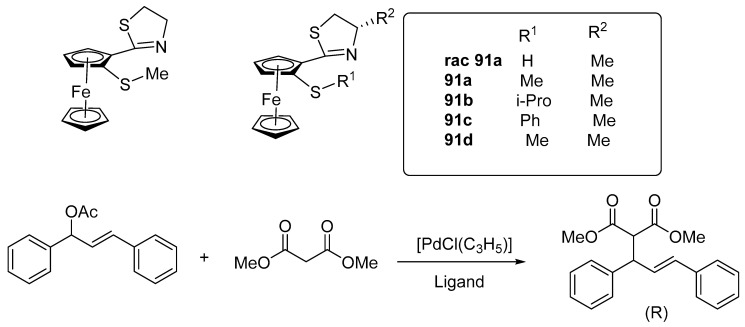
Chiral bidentate [N, S]–ferrocene ligands based on thiazoline used in palladium-catalyzed asymmetric allylic alkylation.

Ferrocene derivatives bearing donating groups exhibit a large variety of applications, especially in metal-catalyzed modern organic reactions. Based on the impetus provided using thiazoline–oxazoline ligands (**91a**–**d**), the investigations directed towards the synthesis of asymmetric disubstituted ferrocenyl ligands functionalized on the thiazoline framework were carried out. The ability of these new bidentate [N, S]-ferrocene ligands to act in Pd-assisted asymmetric allylation had also been described and comes with their oxazoline counterparts (Figure 24). Further, the ortho-directing property of thiazoline moiety for stereoselective ortho-lithiation reaction was also observed to always give an endoselective product [97].

#### 3.2.3. Chiral Oxazolinyl Hydroxyl-Clubbed Ferrocene in Asymmetric Reactions

**Figure 25:**
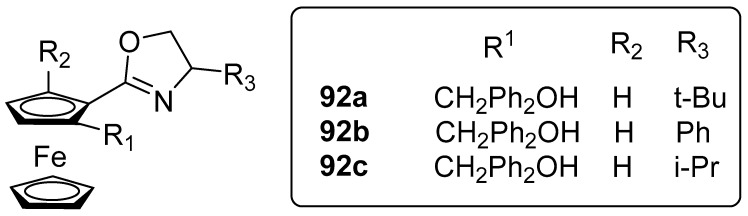
Chiral oxazolinyl hydroxyl ferrocene ligands.

Chiral oxazolinyl hydroxyl ferrocenes have been reported in the literature, particularly the most recent developments in enantioselective applications of ferrocene derivatives and diastereoselective production. Due to the successful functionalization of the aromatic C_p_ ring over the past few decades, chiral ferrocene structural variety has increased. Accordingly, multiple research groups have created sophisticated examples of synthetic procedures for N,O bidentate ferrocene moieties (**92a**–**c**) that have mostly been used as chiral catalysts in zinc-mediated nucleophilic addition reactions to carbonyl compounds. The mechanism subsumes freshly designed competitive catalysts in alkyl and aryl transfers to aldehydes (Figure 25) [98].

#### 3.2.4. Ferrocene Ligand in sp^2^–sp^3^ Coupling Reactions

**Figure 26:**
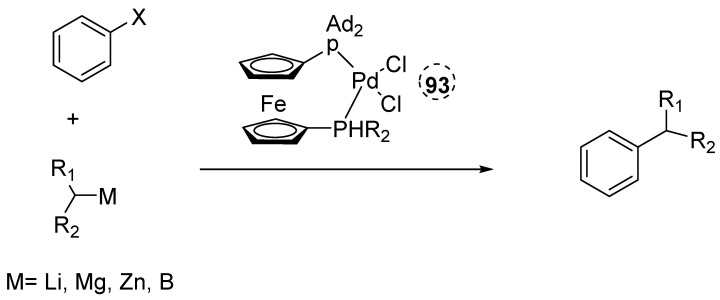
Catalysis by ferrocene ligands bearing a bulky di-1-adamantylphosphino motif.

In an advanced work, Xu and coworkers designed a series of novel ferrocene-based bidentate ligands (**93**) with di(1-adamantyl)phosphine as an anchoring group. After a complete characterization of the ligand structure through XRD, the group applied synthesized chelating catalysts in many C_sp2_–C_sp3_ coupling reactions such as Murahashi–Feringa, Kumada–Corriu, Negishi, and Suzuki–Miyaura, with good to excellent yields. The devised method was employed for the synthesis of nine drug-like molecules in active pharmaceutical ingredient (API) synthesis (Figure 26) [99].

#### 3.2.5. Applications of Ferrocene-Based Diols in Hetero Diels Alder Reactions

**Figure 27:**
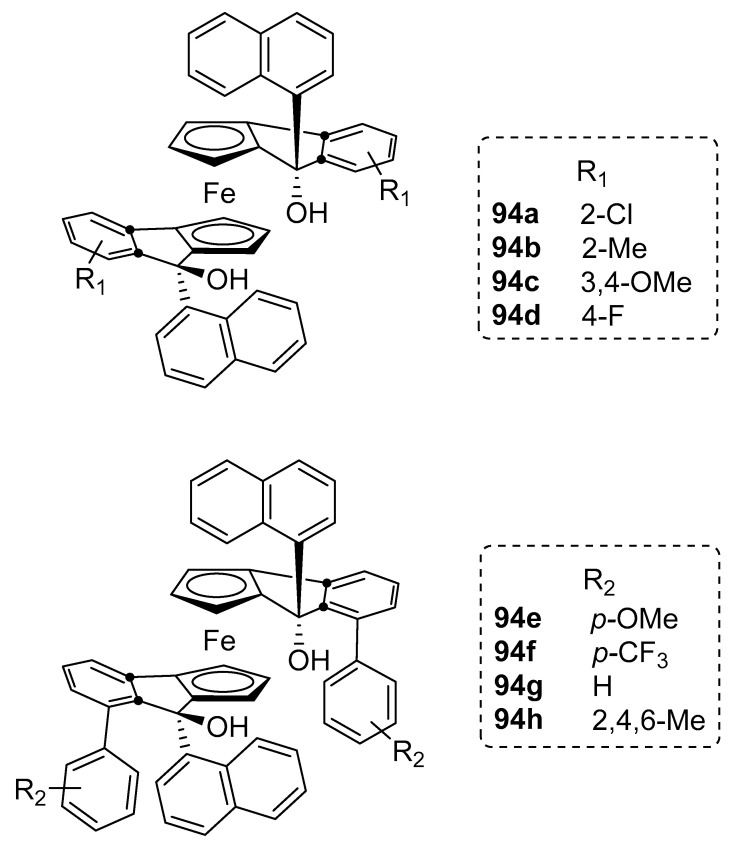
Ferrocene-fused nitrogen heterocycles.

In a completely different work, Cunningham and Guiry reported a synthetic procedure for novel library synthesis of various ferrocene-functionalized diols (**94a**–**h**) with applications in hetero Diels Alder reactions. The stereoselectivity of the prepared ligands was maintained carefully and crystallographic data of such components were reported to confirm their structure. XRD study also revealed that these ligands exhibited both intricate inter- and intramolecular hydrogen bonding, along with uncommon, bifurcated hydrogen bonding interactions (Figure 27) [100].

### 3.3. Industrial Applications

Ferrocene chemistry crisscrossed discipline lines and had a significant part in organic, inorganic, biochemistry, materials science, chemical engineering, etc. These ferrocenyl compounds have recently been developed for industrial use, including in agrochemicals, additives for protective coatings to reduce rusting, fire retardants, rocket propellants, burning rate modifiers, water purification, etc.

#### 3.3.1. Chromatographic Applications

**Figure 28:**
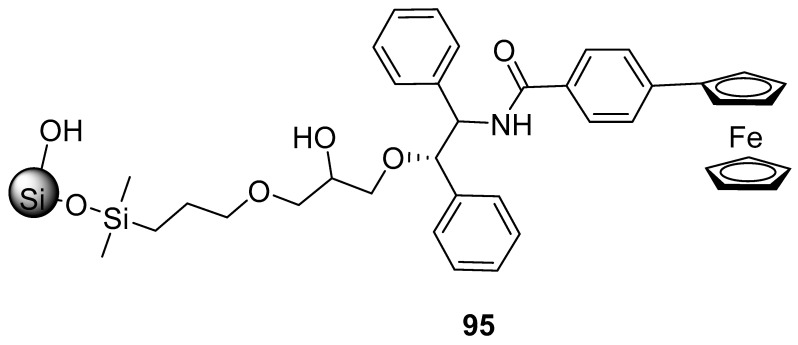
Structure of ferrocene-functionalized stationary phase.

A recent study reported the novel brush-like chiral immobilized stationary phase based on benzoyl-substituted N-ferrocenyl diphenyl ethyl alcohol installed on the surface of silica xerogel [101]. The newly synthesized compound (NF_C_BF_S_) **95** was used in HPLC and the synthesis employed ϒ-glycidoxypropyl trimethoxysilane as a binding agent. Amazing separation among the mixture of aromatic amines, hydrocarbon, substituted phenols, isoquinolines, pyrimidines, imidazole, and other aromatic systems was observed (Figure 28).

In the same way, Wang and partners developed a non-conventional ferrocene-modified monolith that had a complex structure and was prepared through free radical elongation of vinyl ferrocene inside a stainless-steel column [102]. Proteins and other biological composites were separated by applying monolithic column chromatography. The fabricated porous structure displayed high mechanical strength, high adsorption, and low pressure. The different fractions of proteins were separated, and a highly efficient severance of small molecules was attained.

#### 3.3.2. Corrosion Inhibition Potential

**Figure 29:**
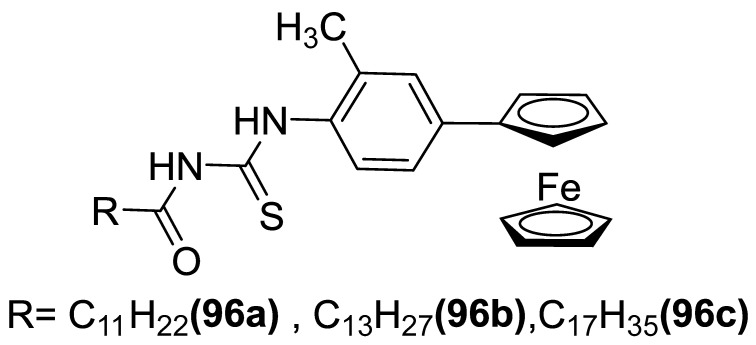
Ferrocene-based corrosion inhibitor motifs.

Novel ferrocene-graded thiourea derivatives with a smaller value of carboxymethyl starch (CMS) inhibition were reported as side-chain dependent [103]. These synthesized compounds (**96a–c**) were efficient corrosion inhibitors for mild carbon steel (MCS) in 1M aggressive hydrochloric acid solution, and their masking ability increased with temperature, concentration, and side-chain length (Figure 29). Results of the degraded product of two sample solutions with or without an inhibitor in HCl solution further confirmed the corrosion inhibition potential of the synthesized compounds.

**Figure 30:**
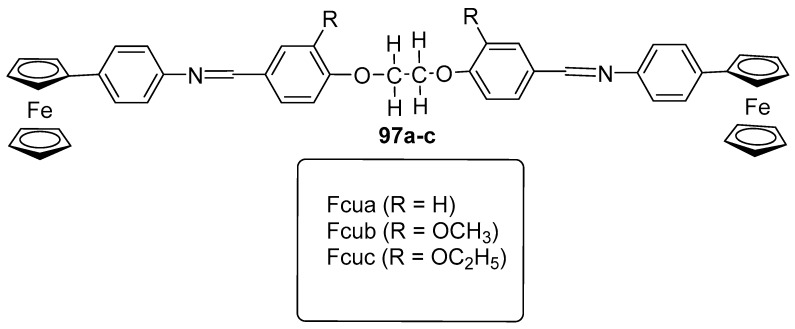
Structure of ferrocenyl Schiff bases (Fcua, Fcub, Fcuc).

Ferrocene-based Schiff bases (**97a–c**) with an extended aromatic system and a large superficial zone have been recently reported (Figure 30). These manufactured bis-ferrocenyl Schiff bases were considered virtuous anticorrosive ingredients for aluminum AA2219-T6 alloy in 0.1 M HCl solution [104]. Further, electron-donating groups OCH_3_ and OC_2_H_5_ bonded to Fcub and Fcuc, consequential in advanced inhibition competence.

#### 3.3.3. Water Treatment

**Figure 31:**
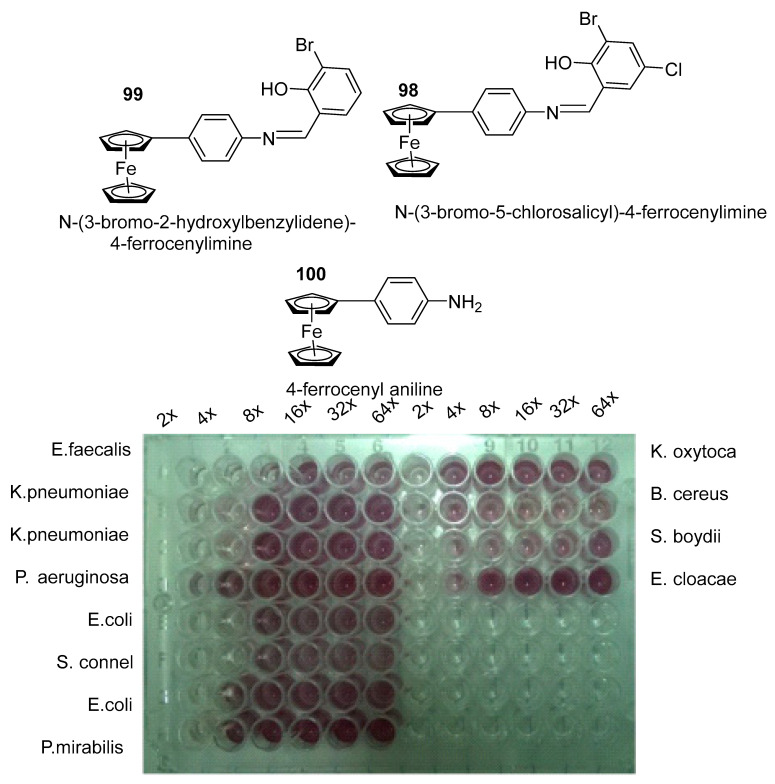
The 96-well microtiter plate layout for the testing of ferrocenylimine compounds.

Traditional methods of water purification by chlorine are commonly used but have serious side effects [105]. One method to resolve these problems was the development of novel eco-friendly water disinfectants based on ferrocenylamine compounds, namely, 4-ferrocenylaniline (98), N-(3-bromo-2-hydroxylbenzylidene)-4-ferrocenylimine (99), and N-(3-bromo-5-chlorosalicyl)-4-ferrocenylimine (100) [104]. The antibacterial activity of these ferrocene derivatives was evaluated against twelve diverse bacterial strains by applying the micro-dilution approach (Figure 31). In vitro bacterial analysis affirmed that the ferrocenylamine species had more remarkable antibacterial activity than its precursor ferrocene and could be used to eradicate bacteria from the aqueous media.

**Figure 32:**
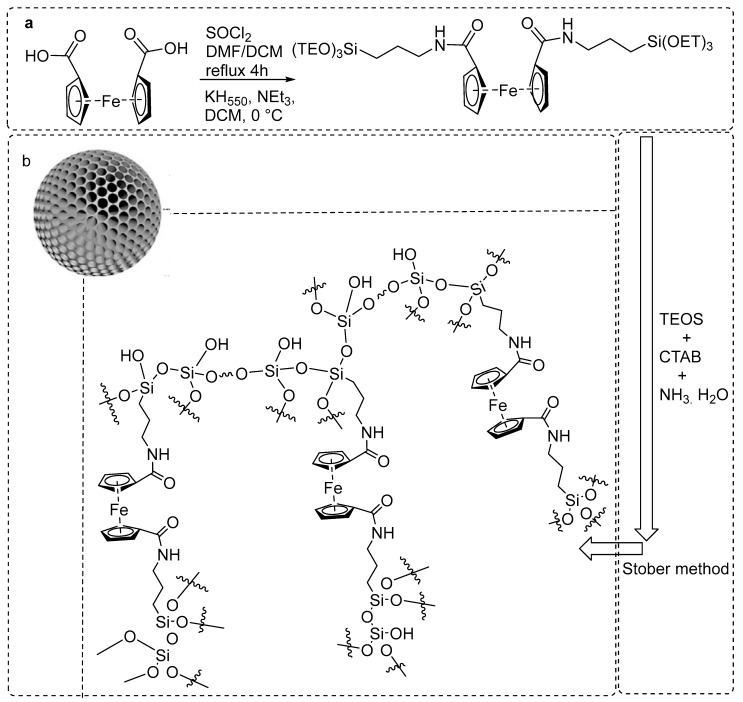
(**a**) Synthetic routes of ferrocene-containing organo-alkoxysilanes and (**b**) schematic illustration of MONs.

Metallocene, especially ferrocene, can encapsulate organic contaminants efficiently as it has a cavity between two cyclopentadienyl rings and the Fe^2+^ center, but its solubility in aqueous media is a notorious drawback. An advanced article reported the solution to this issue via the preparation of novel ferrocene-functionalized organosilica nanoparticles and investigated them for sewage water treatment (Figure 32). The fabricated MONs manifested great absorption power for PO_4_^3−^ within the range of 729, 1299, and 835 mg^−1^/g, and the adsorption capacity for Congo red and Pb^2+^ was also measured and compared with reference SiO_2_-Fe. This comparison confirmed the strong adsorption potential of MONs, and with further modifications, these materials could be used to resolve the issue of water treatment [106].

**Figure 33:**
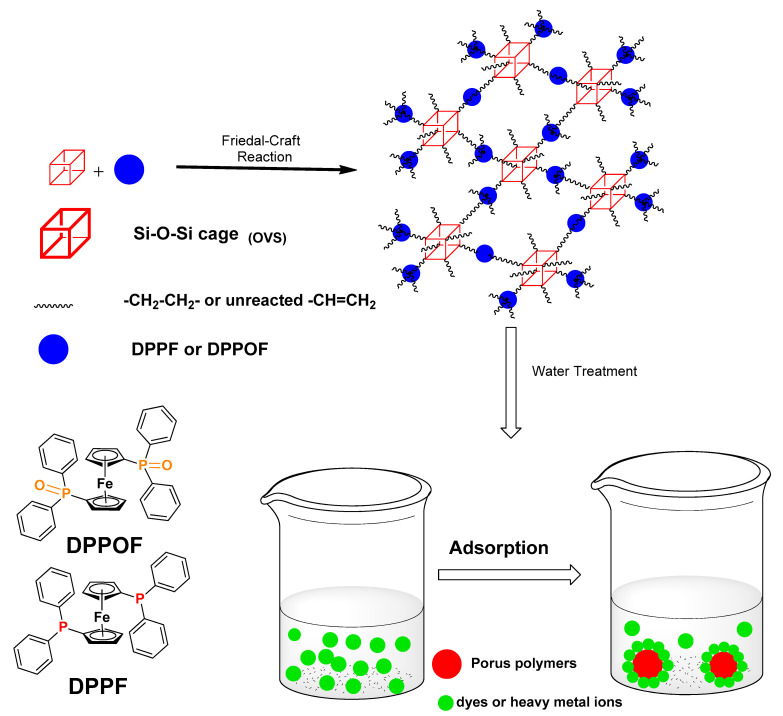
Schematic representation of water treatment through synthesized porous materials.

Yang and co-authors reported a synthesis of two permeable cross-polymers denoted as DPPF-HPP and DPPOF-HPP with excellent surface areas and comparable stoma structures of the concomitant micropores and mesopores [107]. They exhibited excellent adsorption of various colors with reported adsorption dimensions of 2280 mg g^−1^ and 1440 mg g^−1^ for Congo Red and Crystal Violet, respectively. Also, such materials exhibited no mark of degradation under repeated cycles and offered a remarkable potential for wastewater treatment (Figure 33).

#### 3.3.4. Use in Rocket Propellants

**Scheme 44:**
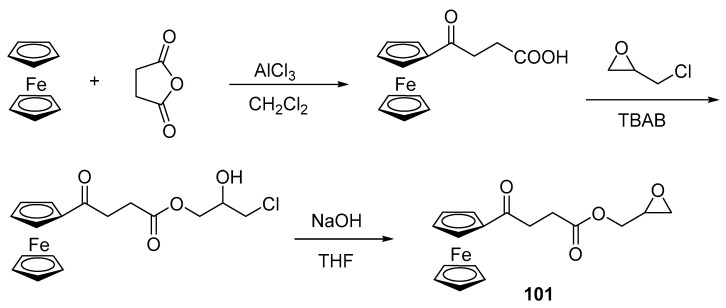
Synthesis of 3-(ferrocenylcarbonyl) propionic acid diglycidyl ester.

The addition of Fc-based BRCs to the propellant system enhances the combustion process and lowers the pressure exponents because of their better ignitability, excellent compatibility, reversible redox behavior, and thermal stability. In this regard, new ferrocene-grafted BRCs, named 3-(ferrocenylcarbonyl) propionic acid diglycidyl ester, were synthesized and their electrochemical properties were extensively studied (Scheme 44). Fc-PADE exhibited remarkable performance in the catalytic decomposition of inorganic oxidizer ammonium perchlorate (AP) and compromising anti-locomotory potential [108]. The novel ferrocene-based epoxy compound (**101**) also had compatible properties for burning rate catalysis in ammonium-perchlorate-based propellant [109].

**Figure 34:**
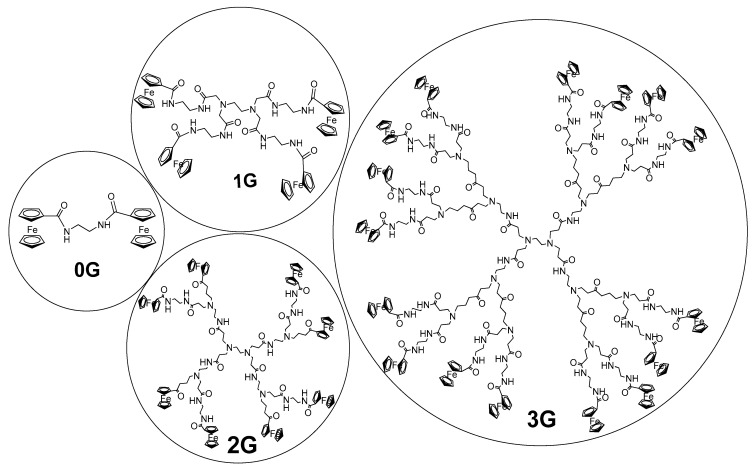
The 0–3G ethylene-diamine-based ferrocene-terminated dendrimers.

The inclusion of ferrocene in highly branched structures enhanced the catalytic behavior of BRCs and can mitigate controlled migratory problems. Using the same concept, Wang’s group reported the synthesis of ethylene-diamine-based ferrocene-terminated dendrimers (0 G, 1 G, 2 G, and 3 G) and evaluated their electrochemical properties (Figure 34). The burning rate catalytic action of all synthesized dendrimers on the thermal breakdown of AP was inspected by TGA and differential thermogravimetry (DTG) techniques. Further anti-locomotory analysis revealed that first, second, and third generations of synthesized materials expressed upgraded anti-migration behavior in the ammonium-perchlorate-based propellant [110].

**Figure 35:**
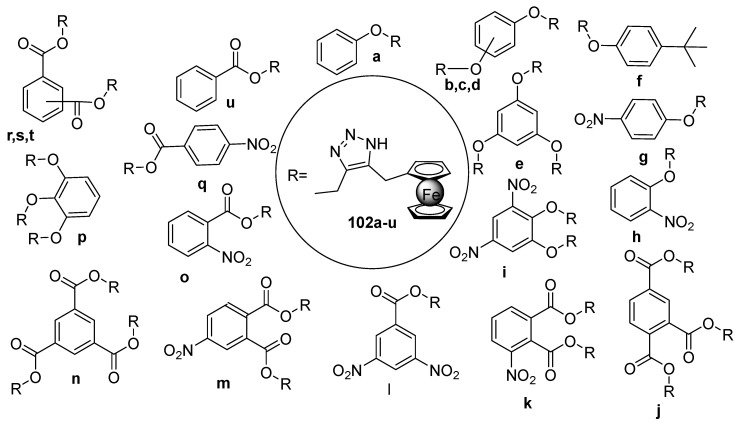
Structures of all ferrocene-grafted triazolyl compounds (F_c−_TAZ_S_).

Cheng and co-workers blended ferrocene-grafted triazolyl compounds (F_c−_TAZ_S_) and characterized their structure through spectroscopic studies (Figure 35). Anti-locomotion and stability aspects of all F_c−_TAZ_S_ were investigated, compared with catocene, and evaluated for their ignition capacity towards the thermal degradation of oxidizing agents [111]. Further, it was observed that the shear degradation potential of ammonium perchlorate could be upgraded by utilizing the triazolyl derivatives **102e**, **102g**, and **102k**.

#### 3.3.5. Applications in BR Catalysis

**Scheme 45:**
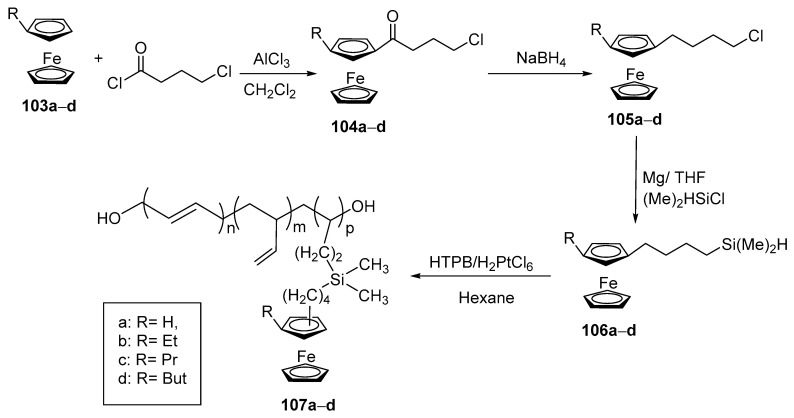
Synthesis of [4-(Dimethylsilyl)butyl] alkylferrocene-appended HTPBs.

In a recent article, Keshver reported a synthesis of ferrocene-based hydroxyl-terminated polybutadiene conjugates **107**(**a**–**d**) through Friedel Crafts acylation reaction and their thermal-stability-related features were studied via thermo-gravimetric experiments (Scheme 45). Various other features like viscosity iron percentage and glass transition temperature confirmed HTPB as a superb burning rate accelerator catalyst for the construction of a composite gas generator [112].

#### 3.3.6. Use in Electrochromic Appliances

**Scheme 46:**
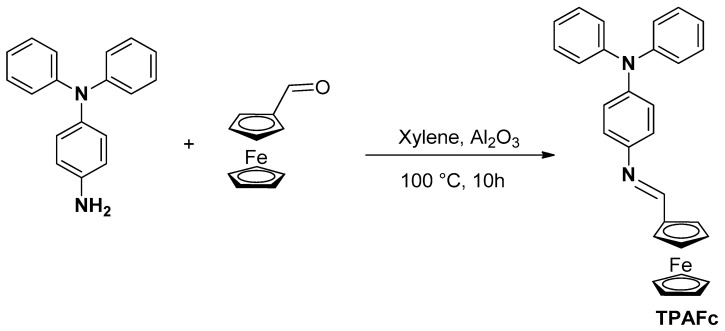
Chemical structure and synthesis route of TPAFc.

Ferrocene (Fc) is widely used as an organometallic functional group owing to its easy modification, excellent electron-donating ability, and single-electron reversible redox reaction potential. A research group performed a synthesis of ferrocene-based triphenylamine derivative (*E*)-*N*-(4-(Diphenylamino) phenyl)-formimidoyl ferrocene) (**TPAFc**) and carried out CV analysis that confirmed a coupling reaction between TPA units and the formation of an electroactive polymer film during successive scans (Scheme 46). These PTPAFc films displayed reversible electrochromic potential, excellent display, and quick switching response, which qualified these materials as an efficient contributor to the operation of electrochromic appliances [113].

#### 3.3.7. Modified Voltammetric Sensors with Ferrocene

**Figure 36:**
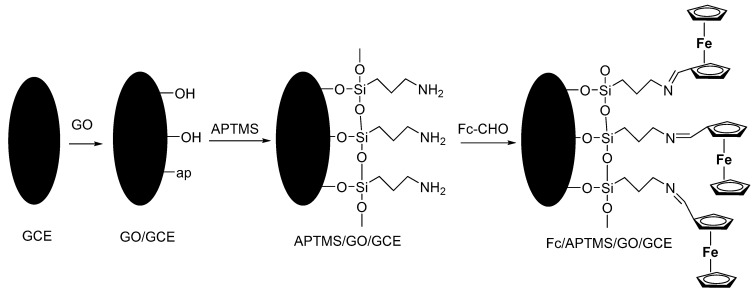
Fabrication of a glassy carbon electrode modified with graphene oxide which was then silanized with (3-aminopropyl) trimethoxysilane and ferrocene carboxaldehyde.

Beitollahi et al. developed modified sensor and biosensor designs. Due to ferrocene’s benefits, numerous electrochemical sensors and biosensors with favorable analytical properties have been developed (Figure 36). The enhanced electrochemical sensitivity is connected to surface fouling resistance, which calls for excellent stability [114].

#### 3.3.8. Application of Ferrocene in Aqueous Redox Flow Battery Suitable for High Temperatures

**Figure 37:**
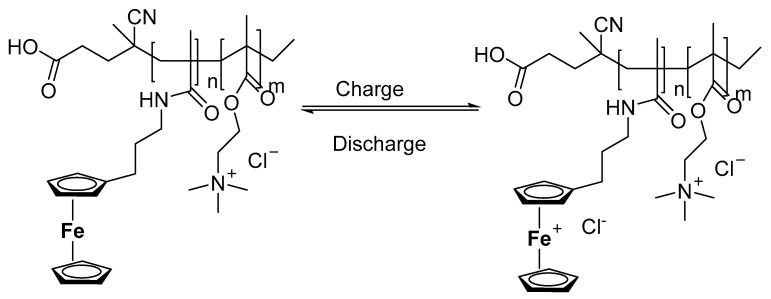
Ferrocene copolymer charging and discharging in redox flow battery.

Using free radicals, the ferrocene monomer was successfully copolymerized with a water-soluble co-monomer. The redox potential of the ferrocene polymer (E1/2 = 0.52 V) in an aqueous environment is marginally higher than that of the low-molar-mass ferrocene derivatives previously disclosed, according to electrochemical characterization (Figure 37). Testing on batteries showed that the polymer exhibited consistent cycling behavior through 100 cycles of charging and discharging at room temperature and 60 °C, with an average Coulombic efficiency of over 99.8% throughout all cycling trials. Investigations into the active materials’ temperature stability showed that they can not only endure high temperatures with ease, but that they actually facilitate the electrochemical reactions. As a result, costly cooling solutions are no longer essential, and the environmental effects can be carefully considered [115].

#### 3.3.9. Ferrocene-Based Non-Enzymatic Hydrogen Peroxide Sensors

**Figure 38:**
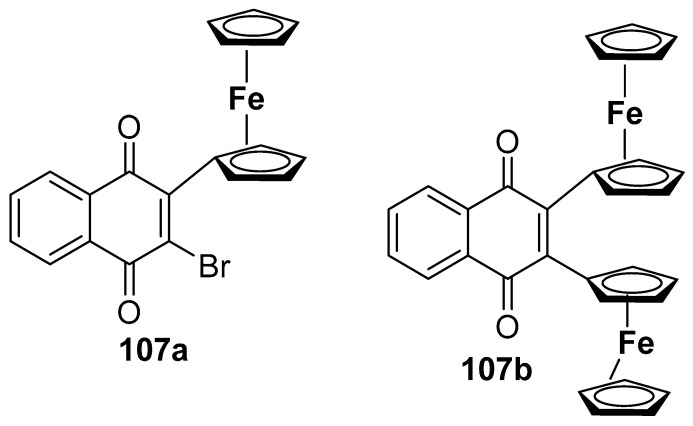
Ferrocene-based naphthoquinone as H_2_O_2_ sensor.

In a recent study, novel ferrocene-based naphthaquinone structures 2,3-diferrocenyl-1,4-naphthoquinone (**107a**) and 2-bromo-3-ferrocenyl-1,4-naphthoquinone (**107b**) were synthesized through the combination of 2,3-Dibromo-1,4-naphthoquinone with ferrocene boronic acid [115]. Additionally, electrochemical characterization methods such as CV, CA, DPV, and EIS were used to examine the H_2_O_2_ detection capabilities of ferrocene-based naphthaquinone derivatives. The voltammetric behavior of H_2_O_2_ at 2-bromo-3-ferrocene-1,4-naphthoquinone in PBS has been studied under ideal conditions (Figure 38) [116].

### 3.4. Agricultural Applications

#### 3.4.1. Herbicide and Pesticide Safeners

**Scheme 47:**
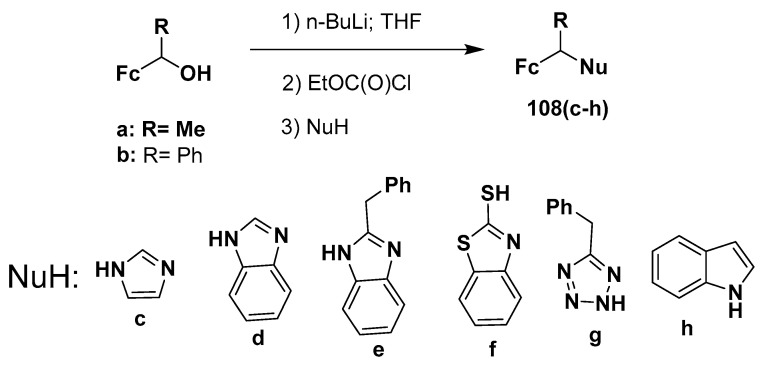
One-pot α-ferrocenylalkylations with α-ferrocenols.

In an auspicious work, ferrocene-functionalized heterocycles **108**(**c–h**) were prepared by treating α-ferrocene alcohol with azole or sulfur nucleophiles in acid-free conditions and investigated for their plant growth regulation effect (Scheme 47). These prepared azole derivatives showed plant vegetation potential and herbicidal findings of growth modulation, and the herbicide antitoxin properties of synthesized azoles were controlled by taking maize seeds and a reference herbicide (60% metasulfuron-methyl). After a comprehensive study, these ferrocene derivatives were considered efficient plant growth activators with herbicide safeness [117].

**Figure 39:**
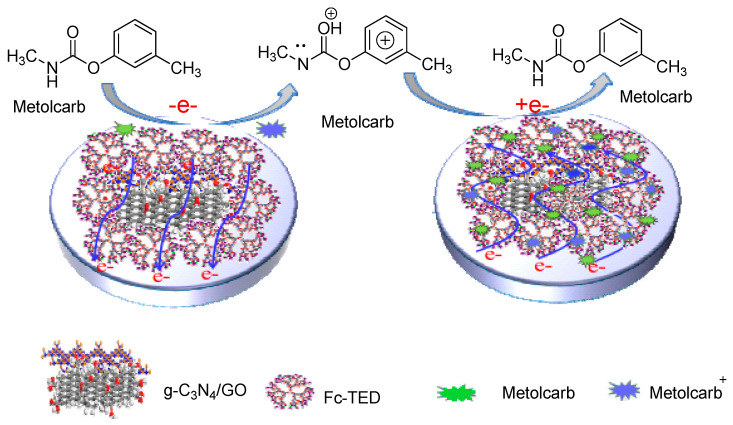
Mechanistic model of the sensor response to Metolcarb.

It is worth mentioning that ferrocenes proved to be useful in evidencing the redox properties of Fe(II) adsorbed onto mineral surfaces. To give an example, an exceptional electrochemical sensor was developed based on graphitic carbon–nitride and graphene oxide nanocomposites covalently linked with a ferrocene scaffold (Figure 39). The synthesized chemosensor exhibited a very low value of the limit of detection for Metolcarb which proved its superiority. The practical use of this sensitive material was found in many fruit and vegetable samples and attained satisfactory results with high precision [118].

#### 3.4.2. Plant Growth Activators

**Scheme 48:**
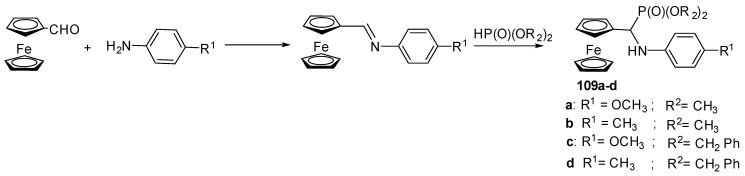
Synthesis of N-aryl amino(ferrocenyl)methylphosphonates.

Lewkoweski and co-workers synthesized ferrocene-appended α-amino-phosphonates separated in the form of crystals with a remarkable yield of 50–70% (Scheme 48). Toxicity assay of compound **109c** was evaluated and described as less harmful for radish and oat plants while **109a** exhibited activity against *Vibrio fischeri.* Among all derivatives, **109d** displayed exceptional behavior, totally harmless to oat seedlings and thought to be the best contender for further investigation with respect to its expected application as a herbicide [119].

#### 3.4.3. Solid-Phase Microextraction for the Determination of Iron Organic Compounds in Seawater and Soil

**Figure 40:**
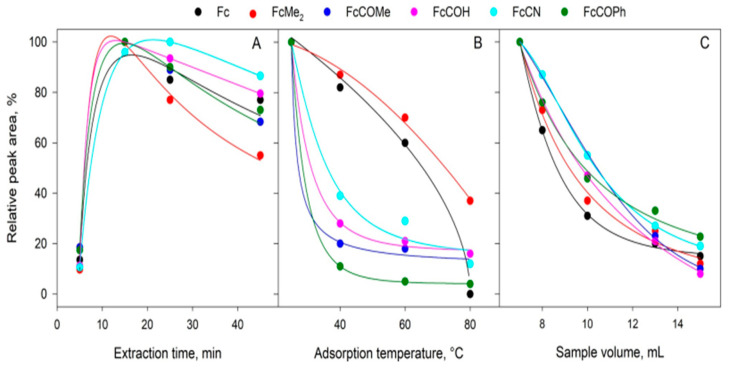
Influence of (**A**) extraction time, (**B**) extraction temperature, and (**C**) sample solution volume on the sensitivity.

In an article, direct immersion of solid-phase microextraction (DI-SPME) and gas chromatography coupled to microwave-induced plasma with atomic emission detection (GC-MIP-AED) were used for the determination of five ferrocene derivatives (1,1′-dimethylferrocene, ferrocene carboxaldehyde, acetylferrocene, ferroceneacetonitrile, and benzoylferrocene) in seawater and soil. For this purpose, commercial fibers made of attached divinylbenzene and polydimethylsiloxane with good extraction efficiency were used in the DI mode. The reported detection limits (DLs) for soil and seawater were 0.9–4 ng g^−1^ and 3–110 pg mL^−1^, respectively (Figure 40) [120].

#### 3.4.4. A Probe-Free Electrochemical Immunosensor for Methyl Jasmonate

**Figure 41:**
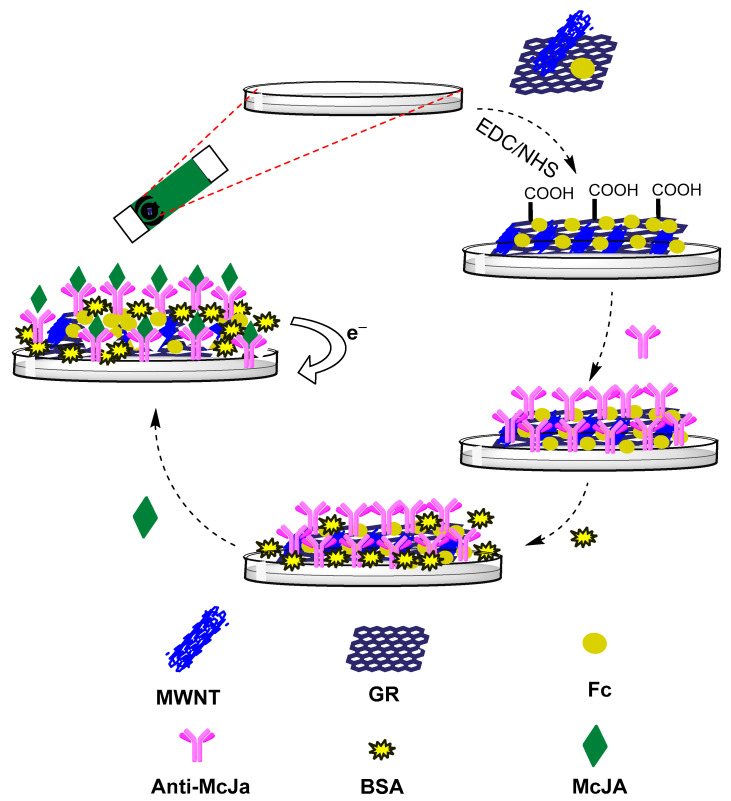
Schematic illustration of the construction process of the electrochemical immunosensor of MeJA.

Methyl jasmonate, an endogenous plant hormone that is crucial to the creation of food, was identified by a unique probeless electrochemical immunosensor accomplished by Xing and coworkers. The procedure includes the attachment of ferrocene with carboxylated graphene (COOH-GR) and multi-walled carbon nanotubes (COOH-MWNT) to produce a composite that was further processed to afford screen-printed electrodes (SPE). The conductivity and catalytic activity of the sensor were enhanced by the application of COOH-GR and COOH-MWNT while keeping the MeJA antibody fixed. As a result, MeJA can be detected using the immunosensor without the use of an external redox probe solution with a very low detection limit, such as 31.26 fM (Figure 41) [121].

#### 3.4.5. Ferrocene-Containing Nitrogen Fertilizers

**Scheme 49:**
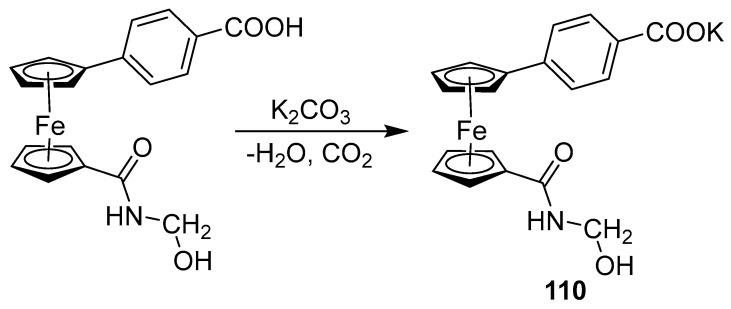
Synthesis of Ferben–potassium fertilizer.

A recent survey reported liquid nitrogen fertilizers containing some derivatives of ferrocene, detailing their use, importance in agriculture, and spectral analysis. Experimental studies revealed that cotton and wheat have better retention of yield elements than control when applying a new Ferben–potassium nitrogen liquid fertilizer during the application period. The final result reported a surge in the yield of cotton by 5–6 ts/ha and wheat by 10–16 ts/ha, indicating high economic efficiency (Scheme 49) [122].

### 3.5. Biological Applications

**Figure 42:**
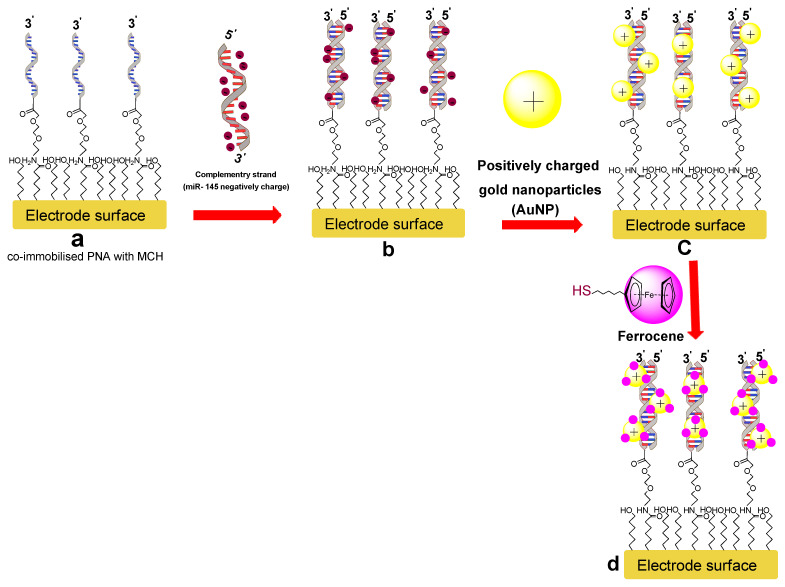
Schematic representation of microRNA detection strategy.

Biochemical uses of ferrocene derivatives are typified by a wealth of contributions. In advanced research, Jolly and group members reported the progress of a vastly penetrating double-mode electrochemical podium for the recognition of microRNAs, which was a significant tool in gene expression activity. The platform was industrialized by consuming peptide nucleic acids as probes on gold electrode surfaces to capture target miRNAs, and thiolated ferrocene offered a harmonizing detection approach by elevating miRNA concentration (Figure 42) [123]. The electrochemical platform could also be readily extended to other miRNA/DNA detection along with the preparation of microarray platforms.

**Figure 43:**
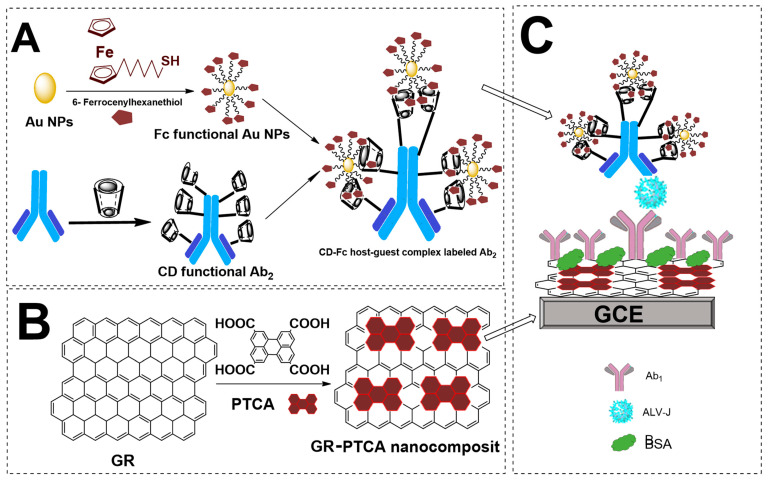
Schematic illustration of the preparation of Fc-AuNP-b-CD-Ab2 (**A**), GR-PTCA (**B**), and the assembly of the electrochemical immunosensor (**C**).

Another handy piece of work described the fabrication protocol of a trifle electrochemical immunosensor for the hypersensitive identification of the Avian Leukosis virus subgroup (Alv-J) using β-cyclodextrin, gold nanoparticles, and ferrocene group host–guest composite as marked and GR-PTCA provide the sensor surface (Figure 43). Impairment of composite material directly onto the surface of the secondary antibody (ab_2_) confirmed its powerful binding affinity to the host. Moreover, it was reported that the immunosensor had higher receptiveness, remarkable reliability, and reusability, recommended for the computable diagnosis of ALv-5 during clinical trials [124].

**Figure 44:**
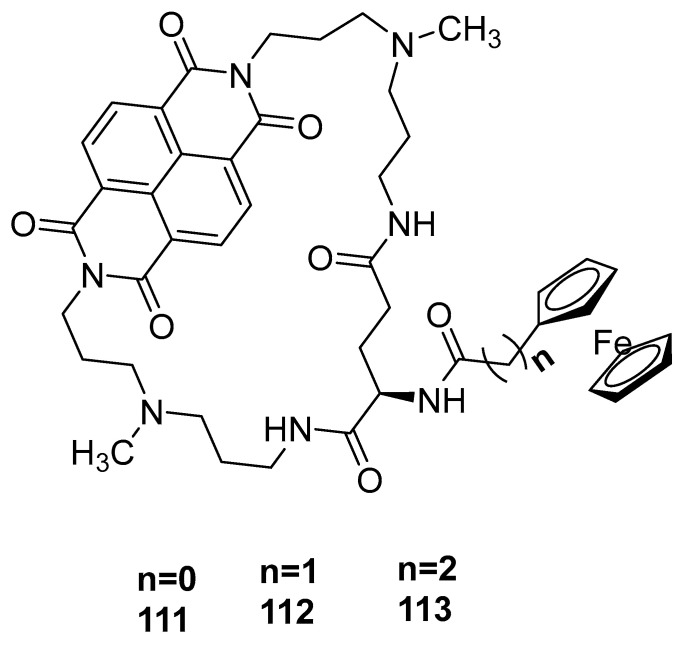
Ferrocene-based naphthalene diimide as DNA-binding ligands.

The DNA–drug complex has attracted great attention recently and the linkage of ferrocene-functionalized drugs with nucleic acids seems to be a “cherry on the top” in the field of medicinal chemistry. In a comprehensive study, a synthetic protocol for the synthesis of a new guanine-rich DNA ligand based on naphthalene diimide (NDI) linked with ferrocene was reported, and the redox potential was analyzed using guanine-rich DNA-immobilized gold electrodes [125]. The redox activity of these ligands was evaluated, and the electrode surface was modified for DNA binding. Moreover, CV analysis described greater selectivity of **111** and **113** for G-quadruplex and realized the anticancer activity of these compounds with a low value of cytotoxicity for normal cells (Figure 44).

**Figure 45:**
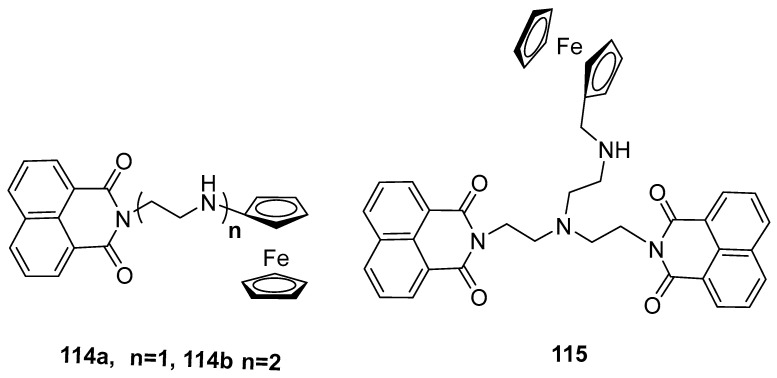
Structure of ferrocene-based ligands.

In a different work, Jia’s group synthesized three ferrocene-based naphthalimides and evaluated their attaching abilities with DNA using ethidium bromide displacement techniques [126]. Among all derivatives, **115** exhibited the highest binding potential for DNA strands with remarkable activity against four different human cancerous cells, evaluated by cytotoxicity tests. The synchronizing potential of the ferrocene nucleus was responsible for the upgradation of cytotoxicity that was linked to the damaged DNA of the cancer cell (Figure 45).

**Figure 46:**
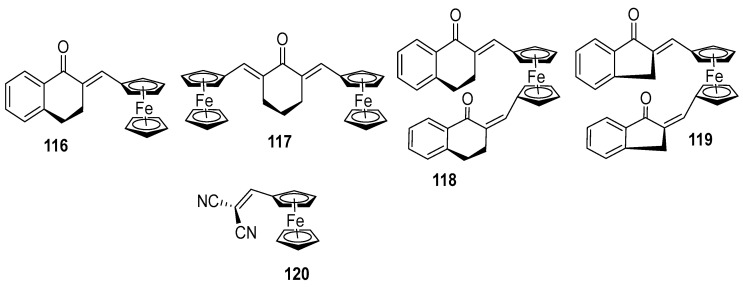
Ferrocene-appended DNA topoisomerase I and II inhibitors.

An array of ferrocenyl chalcones was investigated for their binding affinities with calf thymus DNA and bovine serum albumin, inspected by UV–visible, circular dichroism, and phosphorescence spectroscopy [127]. Likewise, topoisomerase retardation analysis was applied to suppress the action of topoisomerases I and II in humans, and a positive response was observed in a concentration-dependent mode. Further, the interaction between BSA and the five selected ligands (**116–120**) was analyzed by fluorescence quenching spectroscopy (Figure 46).

**Figure 47:**
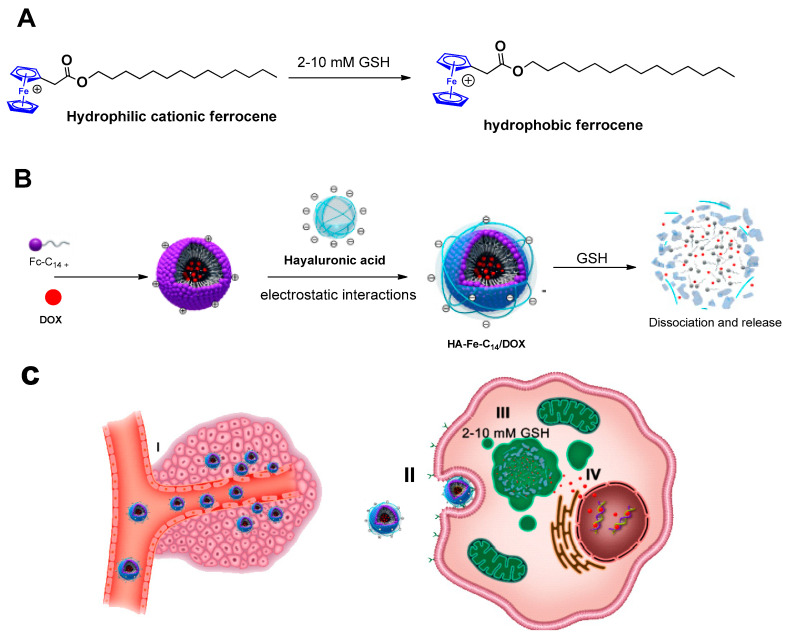
The detailed mechanism of Adriamycin delivery.

Ferrocene derivatives have quickly received significant interest in drug delivery research due to their unique structure, redox character, and hydrophobicity, as they could be transformed into a hydrophilic ferrocenium cation in the presence of a mild oxidizing agent [128]. In this regard, a significant effort was made to design hydrophilic ferrocene tetradecyl units to build up cationic micelles through self-assembly, and the detailed mechanism is described in Figure 47. Micelles were then converted with hyaluronic acid to obtain the HA-Fe-C_14_ complex. These micelles were used for the delivery of Adriamycin drugs, released under a higher GHS environment. The synthesized micelles were concentrated inside tumor cells and revealed highly efficient antitumor potential in both primary and secondary biological screening tests [129].

**Scheme 50:**
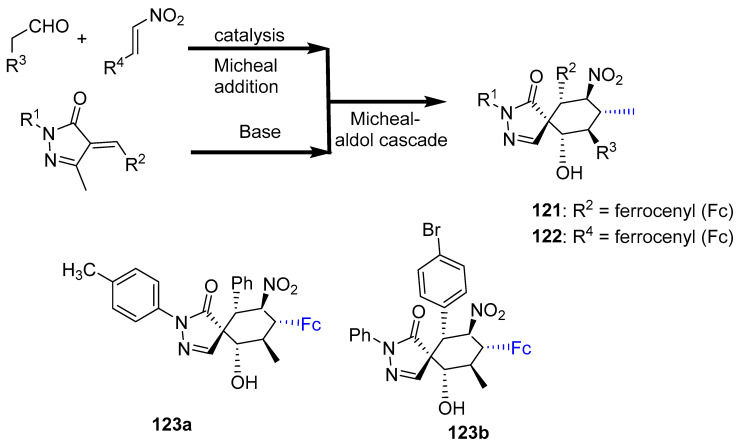
Asymmetric synthesis of chiral spirocyclic pyrazolone ferrocene hybrids.

Recently, poly-functionalized chiral spiropyrazoline–ferrocene hybrids were synthesized through asymmetric organic catalysis and screened for their RalA inhibition potential (Scheme 50) [130]. Among all synthesized compounds, **123a** exhibited powerful RalA inhibition and led to the accretion of RSO and passive spread of pancreatic cancer cells via tempted mitochondrial damage and apoptosis in pancreatic tumor cells. This was a competent application of asymmetric organic catalysis in organometallic bio-organometallic chemistry.

### 3.6. Medicinal Applications

Organometallic compounds like ferrocene have unique properties like low toxicity, low oxidizing potential, water and air stability, and electrochemical aspects that allow them to have wide applications in medicinal chemistry. Nowadays, ferrocene derivatives have found their way into pharmacology as potent HIV, anti-cancer, anti-trypanosomal, antimalarial, anti-diabetic, and antiviral agents, among others. These potent ferrocene-based drugs were either prepared through organic synthesis or developed from already-recommended drugs via drug reprofiling or repurposing strategies in which the structural variation of established drugs with ferrocene scaffolds was reported [131,132].

#### 3.6.1. Anticancer Activity

**Figure 48:**
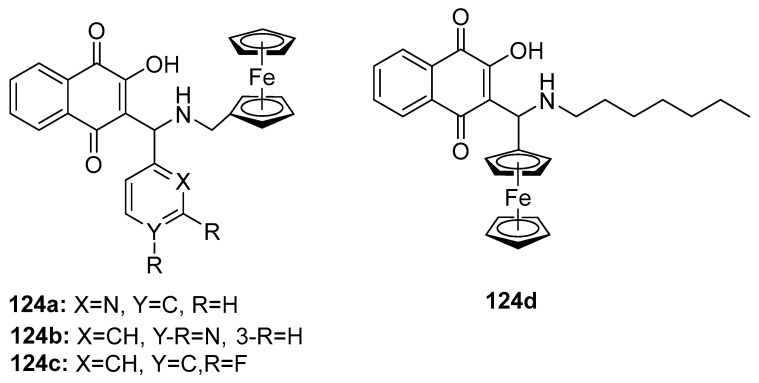
Ferrocene-based Mannich bases derived from Lawsone.

Ferrocene derivatives have been reported to display anti-proliferative activity against several tumor cell lines with less toxic effects in comparison to known anticancer drugs. Since 1970, several types of ferrocenyl compounds were synthesized and subjected to anticancer studies because of their remarkable electrochemical properties and lower toxicity. Ahmad et al. carried out the synthesis of four newly modified Mannich bases of Lawsone that accommodated the Ferrocene units. Lawsone contains a naphthoquinone nucleus whose drug properties were already reported [133]. The biological study showed that compound **124a** is the most active member among a series of four compounds that had maximum antiproliferative activity and it could be a promising lead for the development of ligands that could be used for the treatment of hormone-refractory prostate carcinomas and MDR tumors (Figure 48).

**Figure 49:**
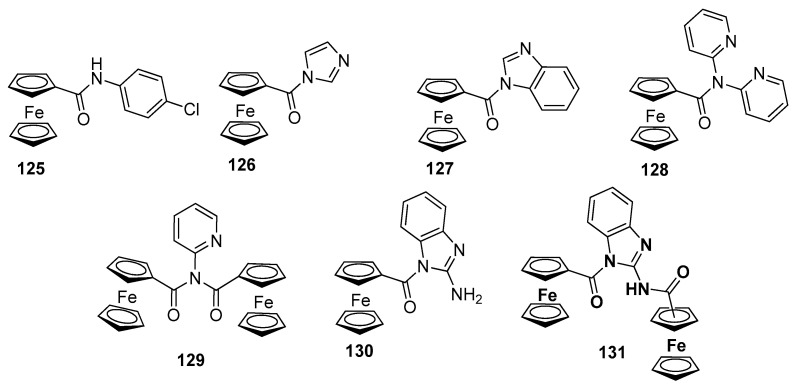
Structures of ferrocene-appended anticancer ligands.

Numerous ferrocene conjugates, including the new N-(*p*-chlorophenyl)-carboxamido ferrocene (**125**–**131**), were synthesized and their antitumor activity was explored in detail [134]. Among these ligands, **126** and **128** with benzimidazole mainstay were potent against HeLa cells, attaining IC_50_ values of ~5 μM and ~6 μM, respectively. Complex **130**, also bearing a benzimidazole support, presented somewhat higher values (~11 μM). These complex mechanisms were thought to be related to a more operative cytotoxic potential (Figure 49).

**Figure 50:**
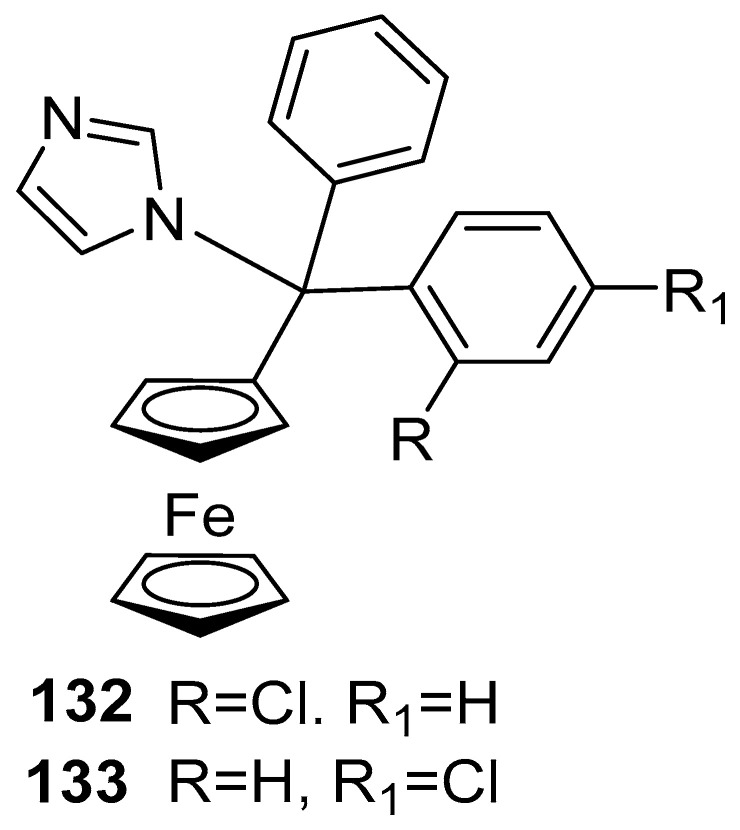
Clotrimazole ferrocene conjugates.

Sonia and Martina synthesized ferrocenyl conjugates of clotrimazole by replacing one of the aryl rings with ferrocene in the triphenylmethane system (Figure 50). Biological screening tests of these derivatives on two different human prostate cells were carried out and compared with the parent drug that exhibited a twofold increment in the cytotoxicity of these ferrocenyl conjugates on HT29 colorectal carcinoma cells. Moreover, commensurable activity was shown against MCF-7 breast cancer cells [135].

**Scheme 51:**
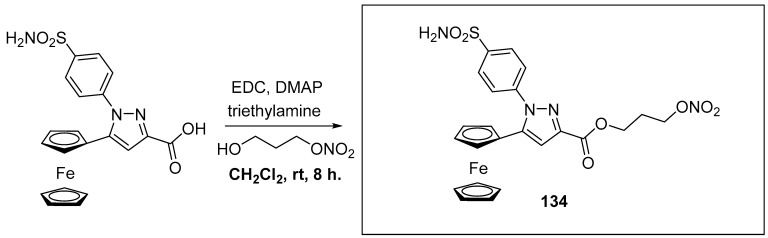
Synthesis of nitric-oxide-bearing pyrazole–ferrocene conjugate.

In an advanced work, unique nitric oxide accommodating pyrazole–ferrocene conjugates were synthesized and reported as COX-2 suppressors [136]. Bioassay analysis of the synthesized compound confirmed the potency of **134** which was the highest among all the synthesized compounds (Scheme 51) and had the maximum anti-propagative capacity and could be destroyed by depolarization of the cell’s powerhouse, and discharge took place not inside HeLa cells. This phenomenon was harmonized with the antiproliferative potential for **134** which is thought to be a suitable drug for cancer therapy.

**Scheme 52:**
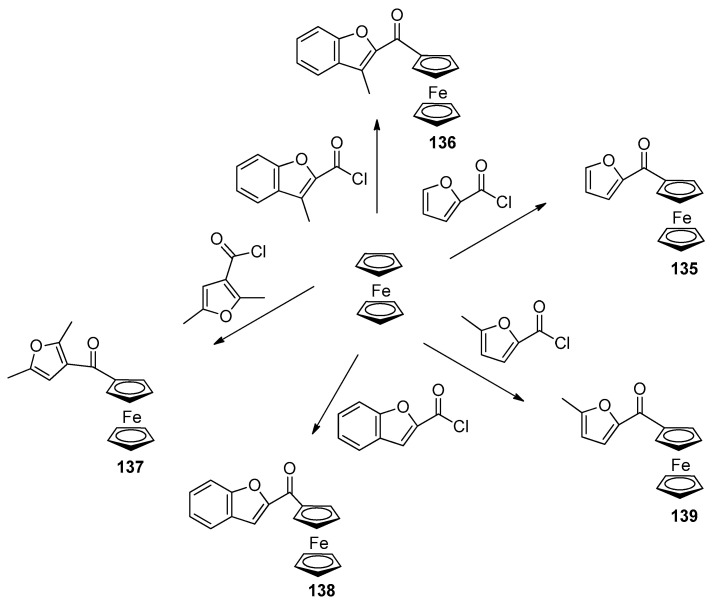
AlCl_3_–EtAlCl_2_-catalyzed ferrocenyl furan derivative synthesis.

Ferrocene and furan derivatives have long been known to be some of the most biologically active compounds [137,138]. Herein, five furoylferrocene analogs were prepared through EAS reaction using AlCl_3_-EtAlCl_2_ Lewis acids (Scheme 52). Though toxicity is directly linked to concentration, at a 100 mg/mL concentration, the protection of cell viability was observed to be 70%. For compounds **135–139**, the necrotic effects were found to be between 21% and 39% at a 50 mg/mL concentration [139].

**Scheme 53:**
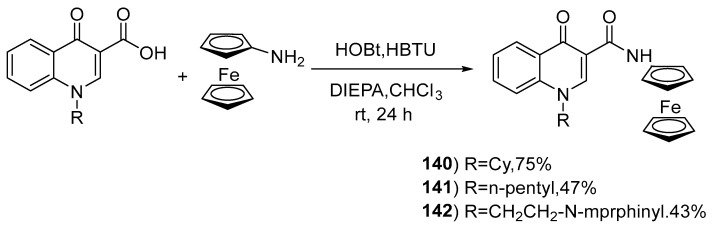
Synthesis of ferrocene-functionalized amide derivatives.

Combinatorial synthesis of ferrocene-linked amides was performed by Sansook and co-workers through an amide coupling reaction (Scheme 53). Three final ferrocene-grafted conjugates were characterized in the solid form by X-ray crystallography and exhibited hydrogen bonding as found in NH---C=O N-methylation of the amide, which was used to carry out a pharmacophore identification study. These synthesized ligands are realized as effective-cannabinoid receptor (CB_1_, and CB_2_) agonists, representing single-digit nanomolar potency [140].

**Figure 51:**
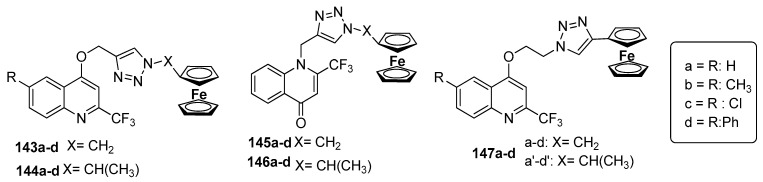
Structures of ferrocene–quinoline conjugates with different O and N substitutions.

Realizing the anticancer potential of quinoline scaffolds, Maracic and colleagues synthesized novel O-substituted and N-substituted 4-quinoline conjugates having ferrocene scaffolds [141]. In vitro analysis exhibited that the N-substituted compound displayed cytostatic activity on selective cancerous cells. Among all N-alkylated derivatives, **145** showed remarkable activity against acute leukemia in blast crisis, whereas **146c** realized a selective inhibitory effect on Raji cells without harming the normal cell (Figure 51).

**Scheme 54:**
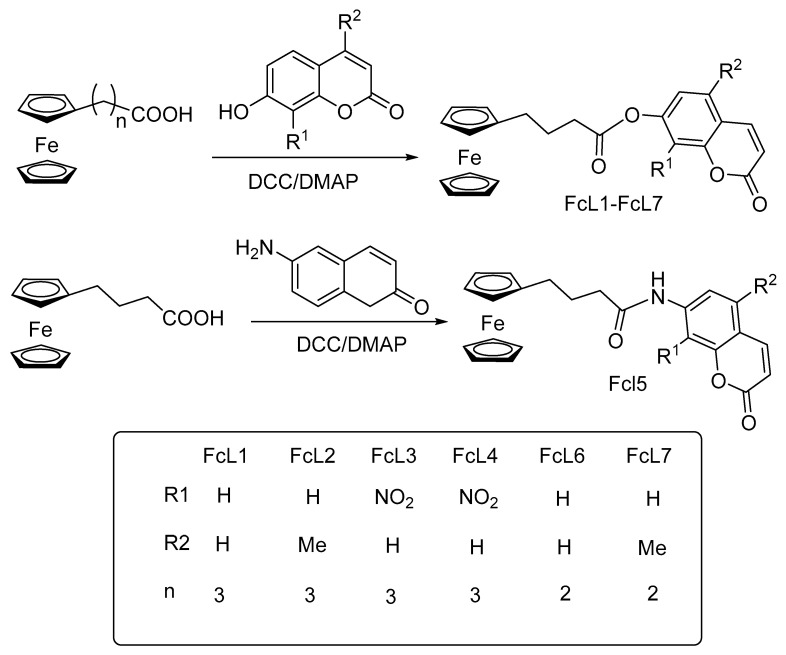
Synthesis of ferrocenyl-substituted coumarin derivatives.

In a different work, a group of Wei and coworkers synthesized a series of seven Ferrocene-based coumarin conjugates and performed their biological screening, with the conclusion that **FcL_1_** and **FcL_5_** showed excellent anticancer potential due to the small value of formal potential E°, and the latter exhibited promising activity against SGC line cells [142]. Exceptionally, **FcL_4_** displayed well-marked cytotoxicity against cancer cells with an IC_50_ value of 1.09 µmol·L^−1^, comparable to Adriamycin (Scheme 54).

#### 3.6.2. Antimalarial Agents

**Figure 52:**
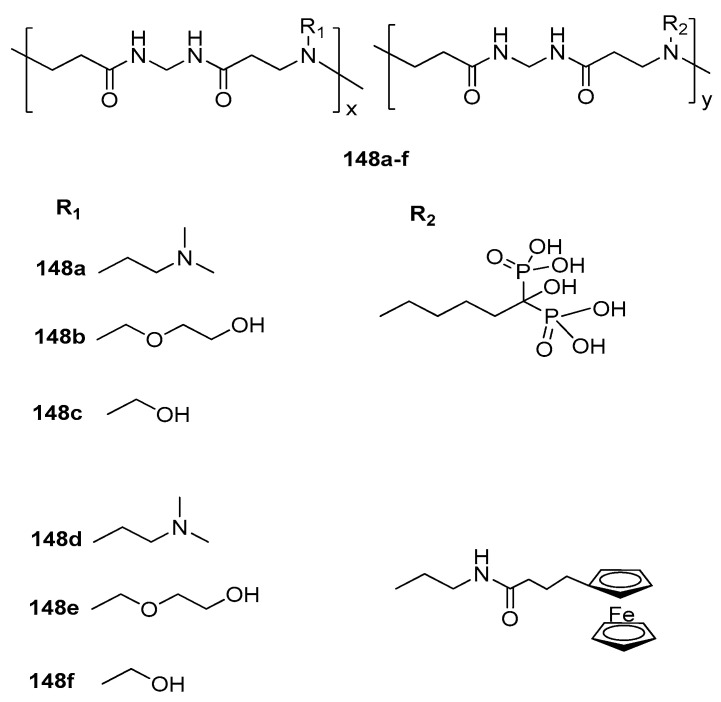
Structure of ferrocene- and neridronate-based conjugates.

Polymeric conjugates, introduced by Ringsdorf, have several benefits over non-polymeric agents due to their high molecular mass and drug release can be delayed, therefore improving drug bioavailability and thus allowing the administration of smaller doses over larger intervals of time. Using the same concept, Mukaya synthesized two groups of polymeric co-conjugates, one bearing ferrocene and the other containing a neridrote moiety, and evaluated their antimalarial activity by secondary bioassay analysis [143]. Among the two groups of drugs, ferrocene-grafted derivatives proved active against a chloroquine-resistant strain of malarial agent, and especially **148e,** along with its co-conjugate, expressed outstanding antimalarial activity. Blood component analysis revealed the selectiveness of these compounds for erythrocytic parasites without harming the host’s red blood cells (Figure 52).

**Figure 53:**
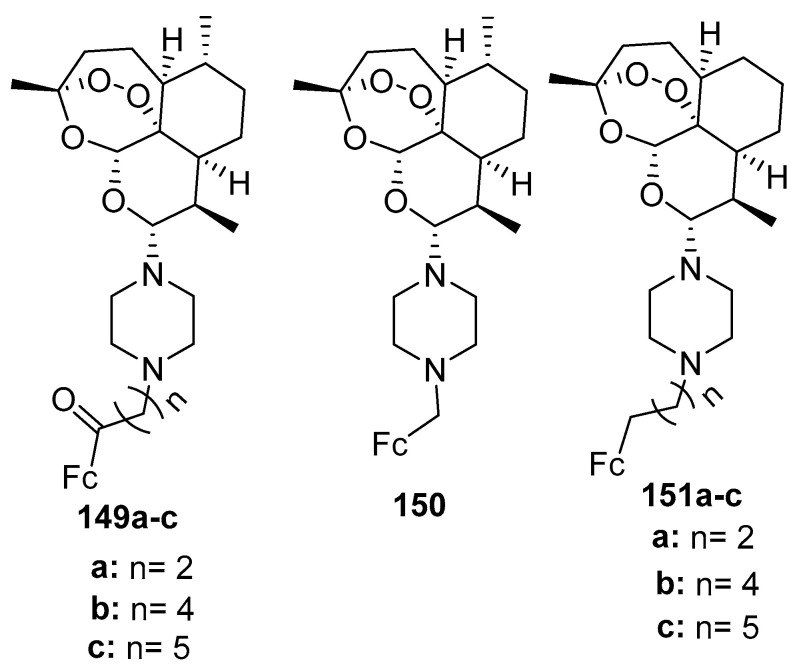
Structure of ferrocene-appended artemisinin conjugates.

Several drugs have been repurposed through ferrocene inclusion, and the most outstanding compound reported so far is ferro-chloroquine, with notable in vitro antimalarial activity against CQ-resistant plasmodium falciparum strains. Recently, a research group reported the synthesis of an unusual ferrocene-functionalized artemisinin ligand through a piperazine bridge and subjected it to biological screening tests on chloroquine cognizant (NF54) and chloroquine refractory strains of the malarial causative agents [144]. Cytotoxicity tests were carried out on fetus kidney cells and they concluded that amino artemisinin derivatives with a ferrocene scaffold exhibited differential activity towards malarial parasites in the company of normal cells (Figure 53).

**Figure 54:**
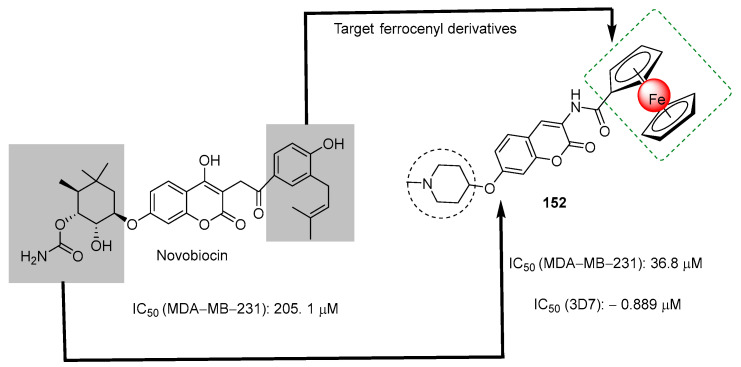
Chemical structures of novobiocin and its ferrocenyl derivative.

An array of advanced cathomycin conjugates was contrived with compromising product amounts by Mbaba and co-authors [145]. Primary biological screening analysis confirmed the antimalarial activity of the target compound **152** towards the falciparum pathogen. It was observed that the ferrocene scaffold elevated the potency of novobiocin derivatives against plasmodium strains. Moreover, a secondary bioassay proved the inhibition face behavior of these conjugates for heat shock proteins Hsp90 (Figure 54).

**Figure 55:**
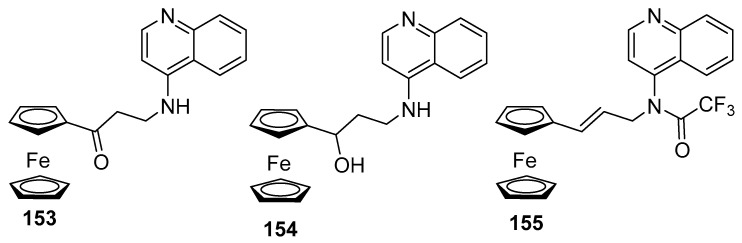
Structures of ferrocene-functionalized quinoline derivatives.

Synthesis of three groups of new quinoline-based ferrocenyl conjugates through an easy and effective study was performed by Minic’s group, and they evaluated their antimalarial activity through a secondary bioassay [146]. It was realized that all three derivatives showed little micro-molar potential towards the chloroquine-effective strain of *P. falciparum*, whereas **154** and **155** also expressed their sub-molecular activity towards the chloroquine-ineffective strain of *P. falciparum*, making them leads for future malarial investigation studies (Figure 55).

#### 3.6.3. Antioxidant Activity

**Figure 56:**
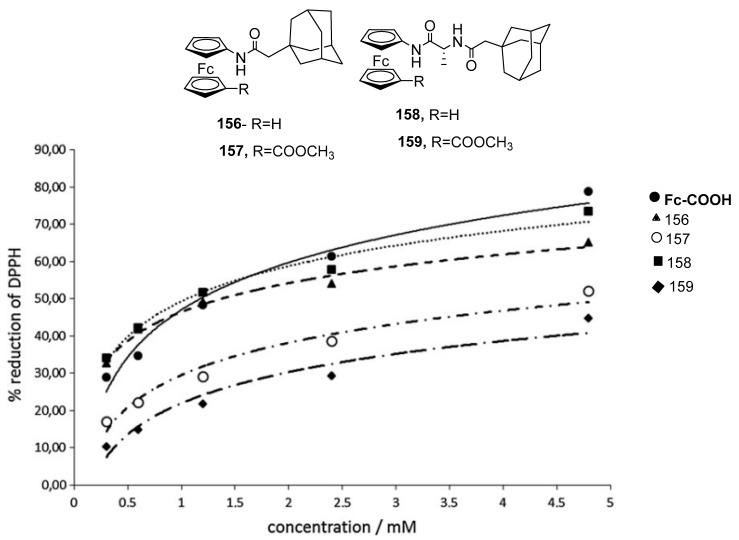
Structure of adamant ferrocene derivatives.

Ferrocene and adamantane are chemically very different but are quite similar in size and hydrophobicity, and the results obtained in these studies open wide possibilities for applications of adamantly ferrocene derivatives. In an article, Stimac and colleagues synthesized and reported the antioxidant potential of ferrocene derivatives through the DPPH free radical scavenging method [147]. The percentage of DPPH quenching in the presence of Fc-COOH and all prepared compounds was measured and shown in a graph exposing that monosubstituted compounds **156** and **158** exhibited better antioxidant potential than disubstituted compounds **157** and **159** (Figure 56). Further, an IC_50_ value of **159** was the minimum among all synthesized ligands, which further confirmed its antioxidant potential.

#### 3.6.4. Antidiabetic Agents

**Figure 57:**
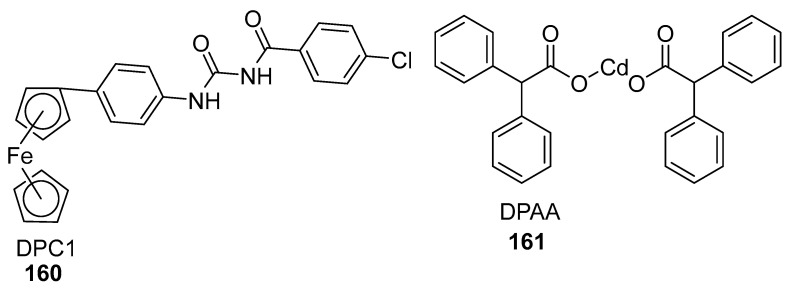
Structure of ferrocene-appended antidiabetic scaffolds.

The group of Bano and Khan recently conducted a detailed study of ferrocene-grafted acyl urea derivatives and homoleptic carboxylates through in silico and in vivo analysis and evaluated their antidiabetic potential [148]. All examined scaffolds showed large binding interactions for target sites, including receptors and enzymes, i.e., aldose reductase, glucokinase, Cα glycosides, etc. In vivo analysis of these compounds on alloxan-coaxed mice confirmed that DPC1 (**160**) and DPAA (**161**) decreased the blood glucose level of the host body mass and dose-dependently (mg/kg) reduced the level of glycosylated hemoglobin in the diabetic host (Figure 57).

#### 3.6.5. Ferrocene-Grafted Anti-Trypanosomal Agents

**Figure 58:**
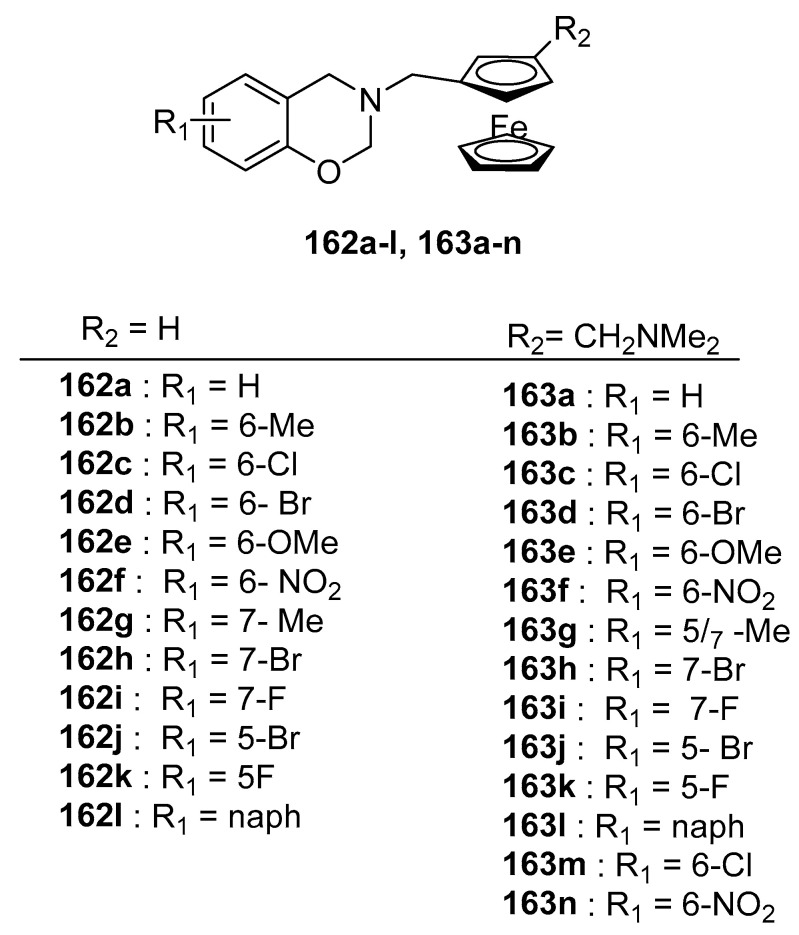
Structure of reprofiled ferrocene-based benzoxazines.

In another interesting example of drug repurposing, Mbaba and assistants developed novel ferrocene 1,3-benzoxazine conjugates by applying a drug reprofiling strategy and carried out their biology screening tests to confirm their anti-trypanosomal activity [149]. The remarkable medicinal potential of these compounds was observed against *T. Brucei*-infected strains with an IC_50_ value of 0.15–38.6 µM. Moreover, compound **163c** showed maximum efficacy due to the side-chain CH_2_NMC_2_O_4_, and a 20-fold rise in the potency of the reprofiled drug was observed (Figure 58).

**Figure 59:**
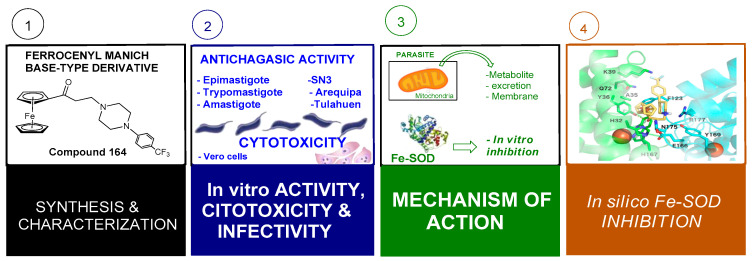
Synthesis, cytotoxicity assay, mode of action, and computational analysis of compound **164**.

Using a different protocol, Paucar and co-researchers developed new ferrocenyl Mannich bases and carried out artificial insemination tests to check out their inhibitory potential toward sleep sickness parasites [150]. Compound **164** showed superior suppressing capacity among all conjugates and the calculated IC_50_ value was also high, reaching the maximum for the Arequipa strain. Further, this compound also minimized many damaged cells through a genotoxic phenomenon. In the end, molecular docking was also performed to find the binding capacities of the examined compound with Fe-SoD enzymes (Figure 59).

#### 3.6.6. Antimicrobial Agent

Antimicrobial agents have been effectively applied to treat patients with micro-organism diseases, but the major problem is that their causative agents have become immune to these drugs. Many drugs that are based on metallocene, especially ferrocene, possess modified pharmacological and toxicological characteristics compared to their original characteristics when administered as metal-based compounds.

**Figure 60:**
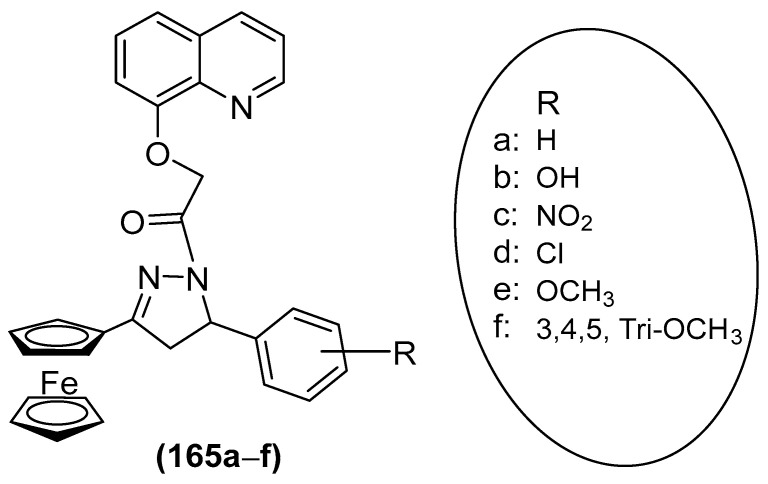
Chemical structure of ferrocene-grafted pyrazoline conjugated.

Parveen et al. explored the synthesis of new pyrazoline-appended ferrocenyl quinoline compounds **165a–f** and checked out their antimicrobial potential [151]. Fifteen bacterial and fungal strains were exposed to these derivatives via the Broth micro-dilution method, and the compounds exhibited extraordinary MIC values against the examined stains. Among all samples, ligands **165d** exhibited remarkable antimicrobial activity by measuring MIC value within the range of 8–32 Hg/mol. These organometallic compounds could be used as part of a two-in-one strategy to synthesize potential antimicrobials (Figure 60).

#### 3.6.7. Ferrocene Hybrids as BET Bromodomain Inhibitors

**Figure 61:**
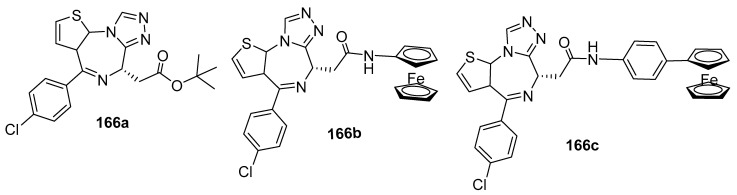
Ferrocene-based BET bromodomain inhibitors.

Hassell’s group synthesized a wisely intended bromodomain inhibitor **166b** that was a rationally designed ferrocene conjugate of the BET bromodomain (BRD) probe molecule **166a** [152]. The newly developed probe **166b** displayed excellent BRD affinity in biochemical assays as well as in pharmacokinetic studies, and a slight rise in cell potency was observed when the same molecule was converted to a purported Fe (III) species in cells after exposure to sodium nitroprusside. The metabolism of the synthesized structure calls for an extensive study of its metabolism in metal-comprising compounds, which may improve their application in clinical settings (Figure 61).

#### 3.6.8. Antiviral Agent

**Figure 62:**
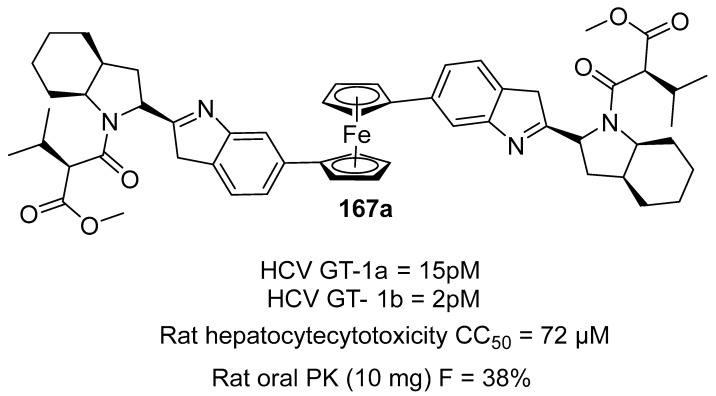
Structures of ferrocene-based HCV NS5A inhibitors.

The synthesis of 1,1-ferrocenediyl conjugates and their HCV GT-1a and GT-1b replicons has been extensively studied by Gadhachanda’s group by exploiting the versatility of ferrocene chemistry [153]. Among all reported compounds, **167a** offered a fragile rotary component that bargains extra conformational flexibility to provide promising affinities with the marked NS5A protein. The QSAR derived after these organometallic materials opened an influential door for the groundbreaking design of the geometrically associated, and favorably pursued, biplanar organic analog Odalasvir (Figure 62).

### 3.7. Electrochemical Insulin Detection

**Figure 63:**
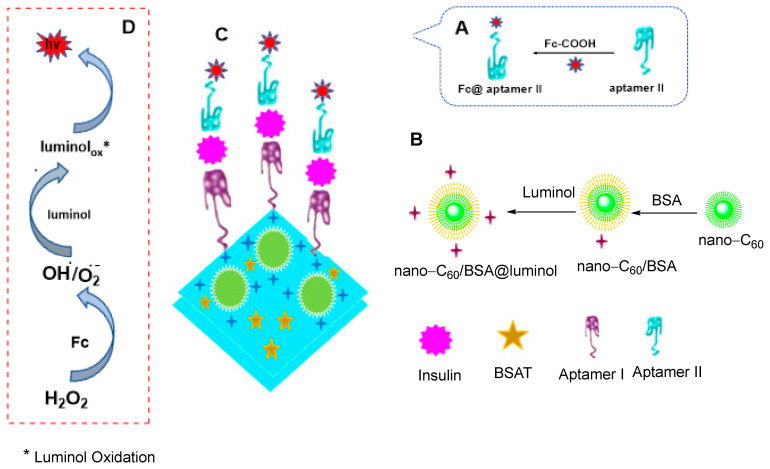
Representative illustration of design, strategy, and fabrication process of the sandwiched ECL sensing platform. (**A**) Formation of Aptamer II from ferrocene-grafted aptamer **II**, (**B**) Formation of BSA-luminol nanocomposite, (**C**) Fabrication of the insulin targeted ECL aptasensor, (**D**) The possible reaction mechanism of ECL aptasensor.

Using an advanced methodology, a stepwise fabrication process of the electrochemical sensor based on a polyfunctional fullerene/BSA-luminol nanocomposite, Fc-aptamer **II**, and a sandwich-like ECL detector was reported and subjected to insulin detection (Figure 63). Ferrocene, with remarkable conductivity and promising catalytic accomplishment for H_2_O_2_, was employed to inactivate aptamer **II** with the maximum capability, and the corresponding ferrocene-grafted aptamer **II** acted as a seizure probe to characterize insulin with greater specificity. Additionally, the constructed ECL aptasensor represented a sensitivity range of 0.0001–1000 ng/mL and a low limit of detection of 0.04 pg./mL (S/N = 3) for insulin [154].

An electrochemical immunosensor was selected as an efficient analytical technique to assess the ultra-sensitive detection of neuron-specific enolase (NSE) in human serum. During the experiment, it was discovered that DPV significantly altered the peak value signals of Cu-MOFs-Au and Fc-L-Cys at potentials of 0.20 V and 0.20 V, respectively. As a result, a ratiometric electrochemical immunosensor for the quantitative determination of NSE was created using Fc-L-Cys as the label for Ab2 and Cu-MOFs-Au as the electrode-sensing surface. DPV tested and examined the immunosensor’s data and performance results. Cu-MOFs can deposit Au nanoparticles on a greater number of sites because they have a large specific surface area in addition to offering the immunosensor the signal it needs. L-cysteine (L-Cys) can significantly reduce Fc-COOH leakage, allowing Fc^+^ to steadily deliver the additional signal that is needed. Cu-MOFs-redox Au’s peak fell while Fc-L-Cys’ redox peak rose as the NSE concentration was increased. At a specific range of values, the ratio (I = ICu/IFc) of two distinct signals was linear with the logarithm of NSE concentration. In short, the immunosensor demonstrated good performance in the concentration range of 1 pg/mL to 1 g/mL, and the detection limit was 0.011 pg/mL, under the optimal experimental conditions. It demonstrated an unexpectedly high sensitivity when compared to other immunosensors. Also, this technique offered a fresh concept for the highly precise quantitative detection of other biomarkers [155].

## 4. Conclusions

This review provides insight into a wide range of aspects regarding the synthesis of organic conjugates functionalized with ferrocene motifs and their multidisciplinary applications. Ferrocene derivatives constitute an important class of organometallic compounds with an extensive range of applications due to the inspiring chemistry of the iron (II) center and the adamancy in aqueous and aerobic media integrated with aromaticity. This recapitulation not only gives access to a great heterogeneity of ferrocene derivatives but also highlights the ability of these derivatives to endure simplistic iron oxidation, which makes them mesmerizing targets in numerous disciplines like materials science, agriculture, industry, electrochemistry, bio-organometallic chemistry, catalysis, and pharmacological applications. The content of this review article is confined to the literature published from 2016 to the start of 2023.

## Data Availability

Not applicable.
